# Evaluating AI-Generated Geriatric Case Studies for Interprofessional Education: Systematic Analysis Across 5 Platforms

**DOI:** 10.2196/83085

**Published:** 2026-01-30

**Authors:** Nicole Ruggiano, Sudikshya Sahoo, Ava Brashear, Uche Nwatu, Amie Brunson, Hyunjin Noh, Heather Cole, Robert McKinney, C Victoria Framil Suarez, Ellen L Brown, Suzanne Prevost

**Affiliations:** 1 School of Social Work University of Alabama Tuscaloosa, AL United States; 2 Department of Kinesiology College of Education University of Alabama Tuscaloosa, AL United States; 3 Capstone College of Nursing University of Alabama Tuscaloosa, AL United States; 4 College of Community Health Sciences University of Alabama Tuscaloosa, AL United States; 5 Nicole Wertheim College of Nursing and Health Sciences Florida International University Miami, AL United States

**Keywords:** chatbots, generative artificial intelligence, geriatric nursing, interprofessional education, patient simulations

## Abstract

**Background:**

Simulation-based learning (SBL) has become standard practice in educating health care professionals to apply their knowledge and skills in patient care. While SBL has demonstrated its value in education, many educators find the process of developing new, unique scenarios to be time-intensive, creating limits to the variety of issues students may experience within educational settings. Generative artificial intelligence (AI) platforms, such as ChatGPT (OpenAI), have emerged as a potential tool for developing simulation case studies more efficiently, though little is known about the performance of AI in generating high-quality case studies for interprofessional education.

**Objective:**

This study aimed to generate geriatric case scenarios across 5 AI platforms by a transdisciplinary team and systematically evaluate them for quality, accuracy, and bias.

**Methods:**

Ten geriatric case studies were generated using the same prompt from 5 different generative AI platforms (N=50): ChatGPT, Claude (Anthropic AI), Copilot (Microsoft), Gemini (Google), and Grok (xAI). An evaluation tool was developed to collect evaluative data to assess the content and quality of each case, sociodemographic data of the featured patient, the appropriateness of each case for interprofessional education, and potential bias. Case quality was evaluated using the Simulation Scenario Evaluation Tool (SSET)*.* Each case was evaluated by 3 team members who had experience in SBL education. Assessment scores were averaged, and qualitative responses were extracted to triangulate patterns found in the quantitative data.

**Results:**

While each AI platform was able to generate 10 unique case studies, the quality of studies varied within and across platforms. Generally, evaluators felt that the content in the cases was accurate, though some cases were not realistic. Some patient populations and common conditions among older adults were underrepresented or absent across the cases. All cases were set within traditional health care settings (eg, hospitals and routine medical visits). No cases featured home-based care. Based on the average SSET scores, reviewers assessed ChatGPT to be the highest overall performer (mean 3.27, SD 0.45, 95% CI 2.95-3.59) while Grok received the lowest scores (mean 1.61, SD 1.26, 95% CI 0.71-2.51). Platforms performed best at generating learning objectives (mean 3.35, SD 1.08, 95% CI 3.04-3.65) and lowest on their ability to describe supplies and materials that may be available in hypothetical scenarios (mean 1.27, SD 0.84, 95% CI 1.03-1.51).

**Conclusions:**

This study is the first to systematically evaluate and compare multiple generative AI platforms for case study generation using a validated assessment tool (SSET) and provides evidence-based guidance on selecting and using AI tools effectively. The findings offer practical direction for educators navigating available generative AI tools to enhance training for health care professionals, including specific strategies for prompt engineering that can improve the quality of SBL resources in interprofessional education. These insights enable educators to leverage AI capabilities while maintaining pedagogical rigor.

## Introduction

### Simulation-Based Learning in Health Care Professional Education

Simulation-based learning (SBL) is an integral component of health care professional education, offering a safe and controlled environment for students to develop clinical skills without risking patient safety [1,2]. SBL is recognized as an effective educational tool that has grown within higher education in many disciplines and can be implemented using a number of approaches, including role-playing, games, computer-assisted virtual instruction, and specially designed skills laboratories [3]. SBL often requires educators to develop highly detailed case studies to guide student learning activities, though these are often challenging to design. Recently, there has been increasing interest in the use of artificial intelligence (AI) platforms to support health care education [4]. While generative AI tools have the potential to generate case studies for use in SBL, little is known about the potential for AI platforms to generate high-quality, effective cases suitable for interprofessional health education. To address this, an interdisciplinary team systematically generated and evaluated geriatric case studies from 5 commercially available generative AI platforms and evaluated their content. The findings have implications for how AI platforms may be appropriately and ethically integrated into SBL.

### History of Simulation-Based Learning in Interprofessional Education

The use of simulation in health care dates back decades, with early examples involving standardized patients, who were individuals trained to portray real patients [5]. This innovation allowed medical trainees to practice patient interactions and diagnostic skills in realistic scenarios. Advancements in technology have enhanced the fidelity, effectiveness, and types of simulation tools, leading to widespread adoption across various health care disciplines [6]. For example, the introduction of high-fidelity mannequins and virtual reality platforms has enabled the replication of complex clinical scenarios, thereby enhancing the realism and effectiveness of training sessions [7]. These technological innovations have expanded the scope of SBL, allowing for the practice of rare or high-risk procedures in a risk-free setting, which is crucial for developing competence in various medical specialties. Recent literature underscores the efficacy of SBL in improving clinical competencies among health care professionals. A systematic review by Sawaya et al [8] highlighted that SBL not only enhances immediate knowledge and skill acquisition but also contributes to long-term retention of clinical competencies. Furthermore, the integration of SBL into medical curricula has been associated with improved patient outcomes and a reduction in medical errors, emphasizing its critical role in contemporary health care professional education.

### The Benefits and Drawbacks of Simulation-Based Education

One of the primary advantages of SBL is its ability to create a safe learning environment where learners can engage in high-risk scenarios or practice clinical skills without the risks associated with working with actual patients [9,10]. This immersive learning experience is further enhanced using high-fidelity simulators that mimic real patient responses, thereby engaging students emotionally and cognitively and improving the transfer of skills to clinical practice [9,11]. SBL has also been shown to improve learners’ self-confidence and satisfaction. For example, previous studies have found that students who participated in SBL in emergency medicine reported higher satisfaction and performed better on assessments compared to those who received traditional instruction [12-14]. This suggests that SBL not only enhances knowledge acquisition but also fosters a positive learning experience. Interprofessional SBL may also emphasize the importance of teamwork in geriatric care, which promotes better collaboration and communication among health care professionals while providing a protected environment where trainees can practice handling complex clinical situations and learn from mistakes in a supportive setting [15-17]. Group-based SBL also promotes active engagement with equipment, simulated patients, and peers, leading to a deeper understanding and increased motivation to learn [18]. This interactive environment stimulates intellectual curiosity, encouraging critical thinking, questioning, and exploration of new knowledge, which aligns with curricula goals to foster curiosity and critical thinking in students.

Despite its numerous benefits, SBL is not without its challenges. One significant drawback is that SBL can be time- and resource-intensive (when using a lab setting) and may also pose challenges when integrated into an already crowded curriculum [9]. Lin et al [19] have asserted that ignoring the implementation and sustainability of SBL can result in higher costs, wasted resources, and the potential failure of educational interventions that are otherwise effective in achieving learning outcomes. Finally, educators may have difficulty in developing scenarios that are realistic and tailored to student learning needs. For example, certain physical findings, such as skin color variations, cannot be adequately represented in certain SBL approaches, which may hinder the comprehensive training of students [9]. Instructors may wish to tailor scenarios to the varying abilities of students, but the standardized nature of many simulation exercises can hinder this individualized approach. This limitation may result in some learners not receiving the optimal level of challenge or support needed for their development.

### Use of AI in Health Professions Education

Over the past decade, there has been increasing interest in identifying potential uses of large language model (LLM) AI tools in education for health care professionals. While the literature has identified opportunities for integrating AI into SBL activities, other potential applications have also been identified [20]. For example, Glauberman et al [20] posit that AI may enhance activities where students explore the impact of social determinants of health on patient care, support tutoring, and provide students with real-time feedback on assignments. However, they also identified potential challenges, such as AI providing hallucinations or biased content, and the risk that students may become over-reliant on AI, which could inhibit critical thinking skills [20].

Given the rising interest, there has been an increase in studies about integrating AI into health education settings. It should be noted that although there are currently multiple commercially available AI LLM platforms, much of the literature on using AI in health education has focused on ChatGPT (OpenAI), due to its rapid adoption among the public, reaching 1 million users in its first 5 days alone [21,22]. In a systematic review of the health education literature, Sallam [23] found that ChatGPT had the potential for creating tailored educational content and communication skill development for students that provides immediate feedback. However, challenges cited in the review included concerns regarding the potential bias and accuracy of information in educational material generated through LLMs.

Rogers et al [24] explored ChatGPT’s capacity for generating simulation scenarios for health care education by evaluating 2 patient simulation cases it generated. Rather than prompting the platform to generate a complete case, in their study, they used a series of prompts to create individual components of a larger case (ie, develop a goal statement, create a corresponding scenario, and add specific detail based on existing information). There were several strengths the reviewers identified in the scenarios, including providing clear learning objectives, creating real-world scenarios, highlighting medication dosing guidelines, and describing participants’ roles for each case [24]. They also identified several weaknesses in the cases, including inaccurate medication dosing, not providing treatment guidelines, missing details on equipment and supplies that would be needed for the case, objectives that would be too advanced for the learner audience, and inaccurate references [24]. Overall, reviewers reported that the main strengths were in the debriefing content, while inaccuracy posed the greatest problem, citing the need for users to fact-check the output of scenarios generated through ChatGPT.

### Rationale for the Study

Geriatric education (or geriatric care) is an increasingly important area of health care professional training, given the growing population of older adults with complex and chronic health needs. As health care systems adapt to meet these challenges, equipping students with the knowledge and skills to provide competent, person-centered care to older adults is essential across disciplines. Generative AI tools have the potential to make some educational tasks more efficient, such as developing scenarios for interprofessional health education simulation exercises. However, the appropriateness of using AI platforms for this task remains unclear. The study by Rodgers et al [24] work evaluated the performance of ChatGPT in creating scenarios; it only evaluated 2 cases and did not compare ChatGPT with other AI platforms. To better understand how generative AI may be used by health educators, this study aimed to evaluate the performance of 5 generative AI platforms in creating high-quality case studies that could be used for interprofessional health education.

## Methods

### Overview

This study involved a systematic evaluation of geriatric case studies that were generated through AI platforms. The methods and results described below reflect the reporting standards for the Generative Artificial Intelligence Tools in Medical Research (GAMER) statement [25], which can be found through the Enhancing the Quality and Transparency of Health Research Network [26].

The cases were generated in December 2024 from 5 different AI platforms: ChatGPT, Claude (Anthropic AI), Gemini (Google), Grok (xAI), and Copilot (Microsoft). Copilot was chosen because it was the platform recommended by the research team’s institution for use in education and research. The other 4 platforms were selected based on their rankings on the LMArena leaderboard, an open-access database of AI platforms developed at the University of California, Berkeley, where users rank various AI platforms on their performance [27]. Before evaluating cases, the team generated 2 cases on each platform’s free version. However, it was discovered that 1 platform (Grok) did not have a free version available, and all the platforms varied in what was offered through their free versions (eg, accessing internet data in real time). Therefore, the decision was made to evaluate cases generated through the paid subscription versions of each platform to minimize bias. Table 1 provides a description and comparison of the platforms.

**Table 1:** Details of the 5 artificial intelligence (AI) platforms selected and their distinguishing qualities.

Platform	Developer	Launch date	Description and distinguishing features
ChatGPT [28]	OpenAI	November 2022 (Public launch)	Built on a generative pretrained transformer (GPT) architecture, the LLM^a^ is trained on conversational data to generate human-like responses to queries.
Claude [29,30]	Anthropic AI	March 2023	LLM incorporating “Constitutional AI” principles based on the UN Declaration of Human Rights and AI^b^ research ethics; emphasizes safety and neutrality through self-critiquing mechanisms.
Gemini [31,32]	Google	2023 (originally as Bard)	Multimodal LLM that processes words, audio, and pictures; designed for productivity and tasks including image generation.
Grok [33].	xAI (Elon Musk)	November 2023	LLM trained using systems such as Kubernetes, JAX, and Rust, designed to create a more efficient AI platform than those previously developed; has access to information in real time through the platform X (formerly Twitter); programmed to have a witty personality.
Copilot [34]	Microsoft	March 2023	Integrates with Microsoft 365 applications; can access users’ personal data to provide tailored responses within a larger productivity suite.

^a^LLM: large language model.

^b^AI: artificial intelligence.

### Generation of Case Studies

To assess the performance of AI platforms in generating geriatric case studies, each platform was asked to generate 10 unique case studies, for a total of 50 cases. The decision was made to submit 10 individual requests for cases on each platform rather than a single request for all 10 after discovering that the platforms created more detailed and robust cases when asked to create them one at a time rather than all at once. The prompts used to generate cases were as follows:

- Initial prompt: generate a geriatric case study that can be used in simulation learning for students from various health care professional disciplines, such as medical, nursing, and social work students.
- Subsequent prompts: generate another unique geriatric case study that can be used in simulation learning for students from various health care professional disciplines, such as medical, nursing, and social work students.

Team members uploaded the cases to a secure cloud storage platform. Two team members (NR and Ava Brashear) blinded the cases so that reviewers would not be able to link any individual case to the platform that generated it.

### Case Study Evaluation Tool

The team developed an evaluation questionnaire for case study evaluators (ie, social work and nursing faculty) to use in assessing the case studies. The following describes the variables assessed.

#### Case Quality

Although there are several tools available to evaluate students’ performance on SBL activities and the quality of debriefing, little work has been done to create assessment tools to evaluate the quality of simulation case content. The research team used the Simulation Scenario Evaluation Tool (SSET), developed by Hernandez et al [35], to evaluate the quality of case studies. The SSET is a standardized assessment tool that was developed using a modified Delphi approach, a structured, iterative process designed to build consensus among experts. The final version of the SSET includes 20 items that are organized into 5 elements, described in Table 2. The SSET also allows the reviewer to skip elements that are not available in the case study description. For each item, evaluators assess cases using a 5-point Likert scale with 3 anchor points. The anchor points are individualized for each item.

**Table 2:** Description of the assessment elements included in the Simulation Scenario Evaluation Tool (SSET).

Element	Element description	Number of items
Learning objectives	If learning objectives are included, the extent to which they are: a good fit for students’ skill and knowledge levels, specific, measurable, action-oriented, relevant, and reflect different types of knowledge or skills.	7
Clinical context and scenario overview	The amount and quality of information provided about the case facilitate learning outcomes.	2
Critical actions	If the case study describes actions or decisions that the student should be able to demonstrate after reading the case study, the extent to which the actions are observable, support the learning objectives, and are attainable for the students’ skill level.	3
Patient states	The details and cues provided to learners can help them carry out the critical actions, including the appropriateness of the case study progression, the ability of learners to take multiple pathways to addressing the case, and how they may help facilitate learning objectives, and require students to take critical actions about the case.	4
Scenario materials and resources	The identification of equipment, supplies, and resources that would normally be available and needed to demonstrate outcomes in clinical skills.	2
Debriefing plan	The extent to which the case guides a postsimulation discussion where learners can receive feedback on their performance.	2

#### Sociodemographic Data

Sociodemographic information (eg, race, gender, and socioeconomic status) from each of the cases was extracted to evaluate the diversity of patient populations presented across cases.

#### Appropriate for Interprofessional Education

Reviewers were asked to rate the extent to which cases were clinically accurate using a Likert scale of 1 (Completely Inaccurate) to 4 (Highly Accurate), with the option of “Unsure.” They were also asked to rate the extent to which the cases adequately addressed ethical considerations using a Likert scale of 1 (Completely Disagree) to 5 (Completely Agree). Reviewers were asked to indicate whether they would use each case for SBL (Yes, No, Unsure). Open-ended questions allowed evaluators to provide feedback on the quality of cases.

#### Potential Bias

Open-ended questions were used to allow evaluators to identify content in each case that they perceived as biased or stereotypical of the patient populations.

### Process for Evaluating Cases

All case studies were generated in November 2024 and were evaluated between December 2024 and April 2025. Two coauthors (SS and UN) extracted the sociodemographic data from each case (eg, patient gender, race, ethnicity, and living situation). Six coauthors (Amie Brunson, ELB, CVFS, HC, RM, and HN) with expertise in interprofessional education in health care settings applied the SSET to evaluate the quality of cases generated. Each case was randomly assigned to 3 evaluators for review. The evaluation tool was uploaded to Qualtrics (Qualtrics International Inc), and each team member entered their assessments remotely and securely into a single database.

### Data Analysis

All data were maintained in a Microsoft Excel spreadsheet for analysis. Descriptive statistics were generated for all of the sociodemographic data, including means, SDs, and CIs. The 3 reviewers’ scores on the SSET for each case were averaged. Qualitative responses were extracted by 2 team members with expertise in qualitative health-related research methodologies (NR and HN) and used to triangulate patterns found in the quantitative data.

### Post Hoc Examination

After the initial evaluation of the 50 case studies generated by AI for this project, a post hoc analysis was conducted to examine how alternative prompt engineering strategies informed by the study's findings could potentially improve the quality of AI-generated case studies. A detailed prompt was developed that addressed weaknesses and gaps observed in the original analysis and was used to generate a single case study using Claude (Anthropic AI). Details about this analysis and its implications for prompt engineering are provided in the “Discussion” section.

### Ethical Considerations

This study did not involve human participants. All data were generated by the AI platforms from information drawn from the internet and other sources on which they had been trained by their developers. No data about real patients were entered into the platforms for this study.

## Results

### Overall Performance

All of the platforms demonstrated the ability to generate comprehensive case studies, though the quality of the cases varied within and across platforms. All cases were organized into content sections, often including a patient profile and demographics, primary presenting complaints of the patient, lists of current medications, past medical history, social history, and assessment results. Some cases generated learning objectives for students without additional prompting. However, for cases generated by Grok, several were incomplete, sometimes being cut off midsentence. For example, one case study generated by Grok featuring the fictitious patient, Mr Jack O’Connor, included, “Current Presentation: Mr O’Connor was referred to...” (sic). In another example featuring Mrs Elena Rodriguez, the case included, “Psychosocial Factors:-...” (sic).

### Variety of Scenario Details

The cases featured fictitious patients who varied in social and demographic backgrounds. There was some variation in the presenting complaints and medical history, though cases tended to emphasize the most common conditions impacting older adults, such as dementia, diabetes, heart disease, and hypertension. Interestingly, none of the 50 cases addressed COVID-19.

Table 3 provides the breakdown of sociodemographic information provided in the patient cases. There was about equal representation of women (n=26) and men (n=24), as well as a variety of races and ethnicities. It should be noted that none of the cases identified the patient as being a member of the lesbian, gay, bisexual, transgender, queer, intersex, asexual, and more community, having undocumented immigration status, or identifying as Native Hawaiian, Alaska Native, or American Indian. There was also a lack of information regarding social determinants of health, most notably income and insurance information. Qualitative data provided by evaluators revealed that the topics of patient finances, culture, and religion were insufficiently addressed in most cases, which did not provide students with adequate context for addressing patient concerns. For example, patients’ decisions regarding treatment or medication, as well as behaviors such as medication noncompliance, may be influenced by their income, cultural background, or religious beliefs.

**Table 3:** Sociodemographic characteristics of the fictitious patients featured in the case studies generated by artificial intelligence (AI) platforms.

Characteristic						Value, n (%)
**Sex**						
		Cisgender male			24 (48)	
		Cisgender female			26 (52)	
**Race and ethnicity**						
		Caucasian or White			9 (18)	
		African American, Black, or Afro-Caribbean			8 (16)	
		Latino or Latina			5 (12)	
		Asian or Pacific Islander			9 (18)	
		Native Hawaiian, Alaska Native, or American Indian			0 (0)	
		Other			2 (4)	
		Unknown			16 (32)	
**English proficiency**						
			Yes		21 (42)	
			No (has a family member interpreting)		3 (6)	
			Unknown		26 (52)	
**Sexual orientation**						
		Heterosexual			29 (58)	
		Unknown			21 (42)	
**Marital status**						
		Married			17 (34)	
		Divorced			5 (10)	
		Widowed			26 (52)	
		Committed partner			1 (2)	
		Unknown			1 (2)	
**Employment**						
			Never worked		1 (2)	
			Retired, not working		45 (90)	
			Working full-time		1 (2)	
			Other		3 (6)	
**Patient household income**						
				Identified as low-income	3 (6)	
				Identified as moderate-income	1 (2)	
				Identified as high-income	0 (0)	
				Unknown or not provided	46 (92)	
**Source of payment for health services**						
		Medicare			11 (22)	
		Medicare and Medicaid			5 (10)	
		Medicare and VA^a^ benefits			2 (4)	
		Medicare with supplemental private insurance			2 (4)	
		Unknown			30 (60)	
**Living situation**						
		Lives at home alone			15 (30)	
		Lives with others in their own home			17 (34)	
		Lives with others in another person’s home			3 (6)	
		Lives in an independent living facility			4 (8)	
		Lives in an assisted living facility			6 (12)	
		Lives in a nursing home or a similar setting			3 (6)	
		Unknown			2 (4)	
**Religious affiliation**						
		Christian (Catholic)			1 (2)	
		Jewish			1 (2)	
		Hindu			1 (2)	
		Other			2 (4)	
		Unknown			45 (90)	
**Immigration and citizenship**						
	Immigrant					4 (8)
	Unknown					46 (92)

^a^VA: Veterans Affairs.

### Case Quality

#### Accuracy and Best Practices

For most cases, evaluators assessed the information provided as being generally accurate and reflecting best practices. However, they reported discrepancies with some case information, most often due to insufficient detail. For example, in reference to the SBL instructions for students, “*Develop a nursing care plan for hip fracture recovery, emphasizing mobility, prevention of pressure ulcers, and patient education on safety at home post”* in the case of Agness Muller (Grok), one evaluator provided qualitative feedback:

This instruction lacks detail and the usual patient progression. For example, a patient living alone with mild cognitive impairment (MCI)—based only on the Montreal Cognitive Assessment, which is not conclusive—and with a hip fracture would not be discharged home.

However, in a few cases, the evaluators felt that the case information was not realistic. For example, in another case of George Hawkins (Claude), one evaluator commented: “*I do not feel like the patient would be coming in stating that he is forgetting things. This information would be coming from the caregiver.*” Similarly, another evaluator commented on the same case: “*I think having the patient present with complaints of confusion would be a little unrealistic. We would likely see the caregiver reporting these findings to the health care provider.*”

#### SSET Scores

For most cases, enough details were provided for reviewers to evaluate the 6 elements represented in the SSET assessment. Yet, a small number of cases lacked details on 1 or more elements. Table 4 shows the average scores assigned by reviewers along with their SDs and CIs for each element across AI platforms.

**Table 4:** Evaluators’ average scores, with SDs and 95% CIs, on the Simulation Scenario Evaluation Tool (SSET) and its 6 elements.

Platform	E^a^1, mean, SD, 95% CI	E2, mean, SD, 95% CI	E3, mean, SD, 95% CI	E4, mean, SD, 95% CI	E5, mean, SD, 95% CI	E6, mean, SD, 95% CI	Overall, platform mean, SD, 95% CI
ChatGPT	4.22, 0.29, 4.01-4.42	3.89, 0.82, 3.30-4.47	3.71, 0.63, 3.26-4.15	3.28, 0.95, 2.60-3.96	1.78, 1.05, 1.02-2.53	2.74, 0.84, 2.14-3.34	3.27, 0.45, 2.95-3.59
Claude	3.74, 0.57, 3.33-4.15	3.59, 1.12, 2.79-4.39	3.34, 0.93, 2.68-4.00	3.50, 1.19, 2.65-4.35	1.58, 0.77, 1.03-2.13	1.70, 0.98, 1.00-2.40	2.91, 0.63, 2.46-3.36
Copilot	3.32, 0.42, 3.02-3.63	2.96, 0.83, 2.36-3.55	2.75, 0.93, 2.08-3.42	2.10, 0.67, 1.62-2.58	0.99, 0.28, 0.78-1.19	1.26, 0.71, 0.75-1.77	2.23, 0.39, 1.95-2.51
Gemini	3.30, 0.49, 2.95-3.66	3.15, 0.77, 2.60-3.71	2.87, 0.66, 2.39-3.35	2.39, 0.73, 1.87-2.91	1.32, 0.84, 0.72-1.92	1.20, 0.66, 0.73-1.67	2.37, 0.49, 2.02-2.72
Grok	2.15, 1.69, 0.93-3.37	2.52, 1.97, 1.10-.93	1.90, 1.45, 0.87-2.94	1.53, 1.38, 0.53-2.52	0.70, 0.73, 0.18-1.22	0.84, 1.03, 0.11-1.58	1.61, 1.26, 0.71-2.51
Overall, E^a^,	3.35, 1.08, 3.04-3.65	3.22, 1.24, 2.87-3.57	2.91, 1.11, 2.60-3.23	2.56, 1.23, 2.21-2.91	1.27, 0.84, 1.03-1.51	1.55, 1.05, 1.25-1.85	2.48, 0.90, 2.22-2.73

^a^E: element.

Generally, the AI platforms performed best and most consistently at providing details related to element 1 (E1: learning objectives). While objectives varied in focus and scope, the platforms generated learning objectives that were detailed. Except for Grok, the mean scores for the platforms were relatively good (range of 3.30-4.22). ChatGPT particularly excelled at generating learning objectives with consistency, given the narrow CIs (mean 4.22, SD 0.29, 95% CI 4.01-4.42). While Grok received lower scores for E1, there was also variability in their quality (mean 2.15, SD 1.69; CI 0.93-3.37).

In some cases, the AI generated a set of learning objectives tailored to the various disciplines that may be involved in the simulation learning activities. Textbox 1 provides an example generated from Grok.

**Learning objectives generated by Grok for the case of Mrs Isabella Bianchi.**

**Medical students:**

- Manage complex medication regimen for multiple chronic conditions, focusing on interactions and side effects.
- Investigate causes of weight loss in older adults, considering both medical and psychological factors.

**Nursing students:**

- Implement and teach strategies for medication adherence in patients with mild cognitive impairment.
- Develop a care plan for preventing falls and managing chronic constipation.

**Social work students:**

- Address the emotional impact of transitioning to assisted living, focusing on preserving dignity and a sense of purpose.
- Explore community resources or programs that could engage Mrs Bianchi’s interest in music, potentially improving her mental health.

**Interdisciplinary goals:**

- Enhance Mrs Bianchi’s quality of life by balancing her medical needs with her personal interests and psychological well-being.
- Coordinate a care plan that includes physical activity tailored to her condition, dietary adjustments for heart health and bone density, and social engagement to combat isolation. 

The AI platforms also performed moderately well on element 2 (E2: clinical context and scenario overview), as evidenced by mean scores ranging from 2.96 to 2.52. Claude (mean 3.59, SD 1.12) and Grok (mean 2.52, SD 1.97) demonstrated the greatest variability in quality.

Evaluators generally felt that the platforms provided sufficient detail for students to understand the presenting case and address the learning objectives. However, they also noted that most cases described clinical contexts occurring within a health care facility (eg, hospital and primary care office), which may limit applicability for students in professions that often interact with patients in other settings (eg, home visits and pharmacies). There were exceptions, such as the example generated by Gemini in Textbox 2, where a home setting was used as the clinical context.

**Clinical context description for the case Mr Arthur Chen generated by Gemini.**

**Simulation activities:**

- Home visit: students can conduct a simulated home visit to assess Mr Chen’s living environment and provide education on disease management and self-care.
- Caregiver support group: students can role-play a caregiver support group to provide Mr Chen with an opportunity to share his experiences and connect with others.
- Interprofessional case conference: students from different disciplines can participate in a case conference to discuss Mr Chen’s care plan and coordinate services.
- Medication management simulation: students can practice medication reconciliation and develop strategies to improve adherence.
- Advance care planning role-play: students can role-play a conversation with Mr Chen and his family about advance care planning.

Element 3 (E3: critical actions) and element 4 (E4: patient states) were rated more modestly by evaluators, with average scores of 2.91 (SD 1.11) and 2.56 (SD 1.23), respectively. Evaluators generally felt that the cases offered enough detail for students to make decisions about patient care and carry out critical actions to support patient care needs. In many cases, a progression of the scenario was presented, as exemplified in Textbox 3.

**Scenario progression for the case of Mr Walter Freemen, generated by Claude (UTI: urinary tract infection).**

**Simulation scenario progression:**

- Initial assessment: each discipline conducts its respective assessments in the emergency department or upon admission.
- Team huddle: interdisciplinary team meets to discuss findings and develop an initial care plan.
- Acute management: simulate management of UTI and delirium, including nonpharmacological interventions.
- Medication review: team collaborates on medication reconciliation, considering potential cognitive effects and drug interactions.
- Family meeting: simulate a meeting with Mrs Evelyn Chen (when more lucid) and her husband to discuss diagnosis, treatment plan, and support needs.
- Discharge planning: team develops a comprehensive plan for transition of care, including medication management, follow-up appointments, and caregiver support.
- Follow-up: simulate a postdischarge follow-up appointment to assess resolution of acute issues and manage chronic conditions. 

Evaluators rated the cases the lowest for element 5 (E5: scenario materials) and element 6 (E6: debriefing plan). All platforms performed poorly on E5 (mean 1.27, SD 0.84, 95% CI 1.03-1.51) and E6 (mean 1.55, SD 1.05, 95% CI 1.25-1.85). For E5, ChatGPT led with an average score of 1.78 (SD 1.05), followed by Claude (mean 1.58, SD 0.77) and Gemini (mean 1.32, SD 0.84). Copilot (mean 0.99, SD 0.28) and Grok (mean 0.70, SD 0.73) had the lowest scores. Evaluators found it difficult to evaluate the supplies and materials needed for students to complete the simulation cases, as they had to make assumptions about what would typically be available in the specific setting (eg, emergency room). However, they acknowledged that greater detail should be provided by most of the platforms to facilitate learning for students who may be less familiar with the setting. For E6, Grok had the lowest score at 0.84 (SD 1.03), followed by Gemini (mean 1.20, SD 0.66), Copilot (mean 1.26, SD 0.71), and Claude (mean 1.70, SD 0.98). ChatGPT fared slightly better in this area with an average of 2.74 (SD 0.84), but still fell short of high performance. Evaluators noted that many cases lacked a debriefing plan, which would require that the educator create one to coincide with the cases.

#### Comparison of Platform Performance

When asked whether they would use the presenting case for simulation learning, evaluators most often stated that they would use the cases generated by ChatGPT and were least likely to use cases generated by Copilot. When evaluators were asked to rate the extent to which they agreed that the case addressed ethical issues that students may experience in health care settings, ChatGPT and Gemini generally received the highest scores (mean 3.50, SD 0.61 and mean 3.51, SD 0.83, respectively), corresponding to “somewhat agree.” Although evaluators thought many of the cases did not sufficiently address issues of culture, they did not report observing examples of gender or racial stereotypes.

Table 4 provides the summary scores for the SSET across platforms. Overall, ChatGPT performed the best in generating quality case studies, while Grok was rated the lowest, mostly due to the incomplete content generated in many of its cases. However, the platforms varied in their strengths and weaknesses. Among all platforms, ChatGPT performed the best in element 1: learning objectives, with the highest average score of 4.22 (SD 0.29), followed by Claude at 3.74 (SD 0.57). Copilot and Gemini had almost identical scores, with averages of 3.32 (SD 0.42) and 3.30 (SD 0.49), respectively, showing only a marginal difference. Grok, however, had the lowest performance in this element, with an average score of just 2.15 (SD 1.69). For element 2, which focused on understanding the relevance and alignment of the clinical context and scenario across case studies, the average scores of ChatGPT (mean 3.89, SD 0.82), Claude (mean 3.59, SD 1.12), and Gemini (mean 3.15, SD 0.77) were relatively close. In contrast, Copilot (mean 2.96, SD 0.83) and Grok (mean 2.52, SD 1.97) had lower averages.

## Discussion

### Principal Findings

This study aimed to evaluate the performance of 5 commercially available AI platforms in their ability to generate geriatric case studies that could be used for simulation learning within interprofessional education settings. Although there was variation in their performance, all 5 platforms evaluated for this study were able to generate comprehensive case studies that generally included accurate information and a variety of patient contexts. ChatGPT was found to be the most reliable of the AI platforms, as demonstrated by consistently high scores and narrower CIs. Grok performed unpredictably and consistently received the lowest scores. It is not surprising that the AI platforms varied in their performance, given that the content generated would be specific to the data used to train each platform and its algorithms. For example, Claude’s algorithm emphasizes a Constitutional AI framework, which may result in it integrating different content than other platforms.

### Implications

A central benefit of using AI for SBL is that educators have the ability to develop a large number of diverse case studies in a short period of time. However, there were some notable shortcomings of the platforms, including their underrepresentation of some vulnerable patient populations, limited attention to cultural sensitivity and religion in health care, and restricted patient settings. The findings have implications for educators who are interested in using AI tools for SBL within interprofessional education settings.

One of the biggest implications is that educators who generate SBL cases using generative AI must provide oversight of the content produced [36]. Research has found that content generated through AI platforms can, at times, be falsely fabricated (ie, hallucinations) or can draw on content that is outdated or biased [37]. This concern was demonstrated in this study, where evaluators felt that some content provided in cases did not adequately reflect real-world scenarios. Another example is that some vulnerable patient populations and medical conditions (such as COVID-19) were not represented in any of the 50 cases. For example, there was a complete absence of any mention of patients who identify as lesbian, gay, bisexual, transgender, queer, intersex, and asexual in any of the selected platforms. To address these shortcomings of generative AI, educators should seek training in “prompt engineering,” which refers to skills in generating prompts for AI tools that are more effective in addressing the goals of the AI user [38]. Meskó [38] indicates that prompt engineering is an important skill for health educators and health care practitioners and requires them to become knowledgeable about how individual AI platforms are designed and function so that they can develop the most effective prompts to meet their needs. In the case of health educators, prompt engineering could support them in developing AI-generated content (SBL case studies or otherwise) that better aligns with the learning objectives they have established for their students.

### The Importance of Prompt Engineering

For this study, the same general prompt was used to develop each case study. Prompt writing is recognized as a skill that can support LLM generative AI tools in tailoring responses for the user [39]. In a previous study by Rogers et al [24], the researchers evaluated the performance of ChatGPT in generating 2 case studies. However, rather than using a general prompt to generate a complete case, they used a series of prompts to guide the platform for each section of the case study (eg, presenting problem and social history). Based on findings from this study, prompt engineering skills could allow educators to more efficiently use generative AI tools that address gaps in content (eg, patient populations and conditions addressed) as well as the quality of the cases generated.

For example, in a post hoc examination, we generated an additional case study using Claude that was more specific regarding quality:

Generate a geriatric case study that can be used in simulation learning for students from various health professional disciplines, such as medical, nursing, and social work students. The content should reflect the five elements of case studies that are emphasized in the Simulation Scenario Evaluation Tool (SSET), developed by Hernandez et al [35]. For social work students, the activity should address the Educational Policy and Accreditation Standards developed by CSWE.

The resulting case study organized the content into learning objectives, clinical context, scenario overview, critical actions, patient states and progression, and scenario materials and resources. Thus, the prompt allowed Claude to build the case with established standards in mind. The resulting case study also linked content to specific Council on Social Work Education accreditation standards, such as asking social work students, “*How did you address social justice issues like financial barriers and access to care?* *(CSWE EPAS Competency 3)*” and “*What advocacy role did you take, and how did you respect Mrs. Martinez's right to self-determination?* *(CSWE EPAS Competency 2)*.”

This approach may help educators optimize the performance of AI tools in generating case studies that meet specific learning goals. Similarly, more tailored prompts could be used to generate cases featuring underrepresented patient populations or specific aspects of case studies that AI platforms may overlook (eg, ethical dilemmas, specific health conditions, or cases in less common health care settings).

### Limitations

There were numerous strengths to the methodology used for this study, including the use of paid subscriptions to the AI platforms to generate cases and having 3 evaluators assess each case using a standardized assessment tool. There were also limitations. First, the case studies were all generated in November 2024, and advancements in AI platforms since then may affect current performance. Second, the team used the paid subscriptions of each AI platform to reduce potential bias from varying access limitations across platforms. However, this may limit generalizability, as most users are more likely to access the free versions of these platforms. It is important to note that both paid and free versions of these platforms have continued to evolve since data collection, making it unclear how results would differ if the study were replicated today with either paid or free versions.

Also, some of the gaps in content that were identified by reviewers across case studies may not have been present if more specific prompts were used (eg, “*Create a case study that features a patient with COVID-19*”). It is also important to highlight that the only perspectives provided about the AI-generated case studies were those of experts in interprofessional health education. The study did not obtain perspectives from students participating in SBL activities. It also did not evaluate how the AI-generated case studies compared to case studies created by professional educators. While this analysis highlighted some of the weaknesses in the AI-generated case studies, they may still be of comparable or higher quality than those generated by humans. Future research should address these gaps.

### Conclusion

The functions and accessibility of generative AI have increased dramatically over the past few years. Health care educators and providers are increasingly using these tools to create efficiencies in their work. In the case of interprofessional health education, AI can support SBL by creating a large variety of case studies in a short period of time. However, as with other applications of AI, human oversight is needed to make sure that the output from these platforms appropriately meets the learning needs of students. Although previous studies have examined AI’s potential role in SBL, this study is the first to systematically evaluate and compare multiple generative AI platforms for case study generation using a validated assessment tool (SSET). By quantifying quality differences across platforms and identifying systematic deficiencies, this research provides evidence-based guidance for educators on selecting and using AI tools effectively. The findings offer practical direction for educators navigating available generative AI tools to enhance training for health care professionals, including specific evidence-based strategies for prompt engineering that can improve the quality of SBL resources in interprofessional education. These insights enable educators to leverage AI capabilities while maintaining pedagogical rigor, ultimately supporting more efficient development of high-quality simulation training materials.

## Appendix

Multimedia Appendix 1 All case studies generated by artificial intelligence (AI) and subsequently evaluated.

## Abbreviations

AI: artificial intelligence
GAMER: Generative Artificial Intelligence Tools in Medical Research
LLM: large language model
SBL: simulation-based learning
SSET: Simulation Scenario Evaluation Tool

## Data Availability

A copy of all 50 case studies generated by the 5 AI platforms is available as a Multimedia Appendix 1. The case studies are blinded to indicate which platform generated each case.

## References

[1] Kim S, Lee H, Connerton TP (2020). How psychological safety affects team performance: mediating role of efficacy and learning behavior. Front Psychol.

[2] Keddington AS, Moore J (2019). Simulation as a method of competency assessment among health care providers: a systematic review. Nurs Educ Perspect.

[3] Sanko JS (2017). A brief history of its use in nursing education. Q Rev Distance Educ.

[4] Zhang W, Cai M, Lee HJ, Evans R, Zhu C, Ming C (2023). AI in medical education: global situation, effects and challenges. Educ Inf Technol.

[5] Flanagan OL, Cummings KM (2023). Standardized patients in medical education: a review of the literature. Cureus.

[6] Englander R, Cameron T, Ballard AJ, Dodge J, Bull J, Aschenbrener CA (2013). Toward a common taxonomy of competency domains for the health professions and competencies for physicians. Acad Med.

[7] Aebersold M (2016). The history of simulation and its impact on the future. AACN Adv Crit Care.

[8] Sawaya RD, Mrad S, Rajha E, Saleh R, Rice J (2021). Simulation-based curriculum development: lessons learnt in global health education. BMC Med Educ.

[9] Krishnan D, Keloth AV, Ubedulla S (2017). Pros and cons of simulation in medical education: a review. Int J Med Health Res.

[10] Siew AL, Wong JW, Chan E (2021). Effectiveness of simulated patients in geriatric education: a scoping review. Nurse Educ Today.

[11] Weller JM, Nestel D, Marshall SD, Brooks PM, Conn JJ (2012). Simulation in clinical teaching and learning. Med J Aust.

[12] Alanazi A, Nicholson N, Thomas S (2017). The use of simulation training to improve knowledge, skills, and confidence among healthcare students: a systematic review. Internet J Allied Health Sci Pract.

[13] Keiser MM, Turkelson C (2019). Using simulation to evaluate clinical performance and reasoning in adult-geriatric acute care nurse practitioner students. J Nurs Educ.

[14] Mehdi Z, Ross A, Reedy G, Roots A, Ernst T, Jaye P, Birns J (2014). Simulation training for geriatric medicine. Clin Teach.

[15] Braude P, Reedy G, Dasgupta D, Dimmock V, Jaye P, Birns J (2015). Evaluation of a simulation training programme for geriatric medicine. Age Ageing.

[16] Langdalen H, Abrahamsen EB, Sollid SJM, Sørskår LIK, Abrahamsen HB (2018). A comparative study on the frequency of simulation-based training and assessment of non-technical skills in the Norwegian ground ambulance services and helicopter emergency medical services. BMC Health Serv Res.

[17] Lateef F (2010). Simulation-based learning: just like the real thing. J Emerg Trauma Shock.

[18] Bland AJ, Tobbell J (2016). Towards an understanding of the attributes of simulation that enable learning in undergraduate nurse education: a grounded theory study. Nurse Educ Today.

[19] Lin Y, Cheng A, Hecker K, Grant V, Currie GR (2018). Implementing economic evaluation in simulation-based medical education: challenges and opportunities. Med Educ.

[20] Glauberman G, Ito-Fujita A, Katz S, Callahan J (2023). Artificial intelligence in nursing education: opportunities and challenges. Hawaii J Health Soc Welf.

[21] Lee H (2024). The rise of ChatGPT: exploring its potential in medical education. Anat Sci Educ.

[22] Wu T, He S, Liu J, Sun S, Liu K, Han Q, Tang Y (2023). A brief overview of ChatGPT: the history, status quo and potential future development. IEEE/CAA J Autom Sinica.

[23] Sallam M (2023). ChatGPT utility in healthcare education, research, and practice: systematic review on the promising perspectives and valid concerns. Healthcare (Basel).

[24] Rodgers DL, Needler M, Robinson A, Barnes R, Brosche T, Hernandez J, Poore J, VandeKoppel P, Ahmed R (2023). Artificial intelligence and the simulationists. Simul Healthc.

[25] Luo X, Tham YC, Giuffrè M, Ranisch R, Daher M, Lam K, Eriksen AV, Hsu C, Ozaki A, Moraes FYD, Khanna S, Su K, Begagić E, Bian Z, Chen Y, Estill J, GAMER Working Group (2025). Reporting guideline for the use of generative artificial intelligence tools in medical research: the GAMER statement. BMJ Evid Based Med.

[26] (2025). Reporting guidelines. EQUATOR Network.

[27] Zheng L, Sheng Y, Chiang WL (2024). Leaderboard overview. LMArena.

[28] Ray PP (2023). ChatGPT: a comprehensive review on background, applications, key challenges, bias, ethics, limitations and future scope. Internet Things Cyber-Phys Syst.

[29] (2023). Introducing Claude. Anthropic.

[30] (2024). What is Claude AI?. Claude AI.

[31] (2024). An overview of the Gemini app. Gemini.

[32] Perera PL, Lankathilake M (2023). Preparing to revolutionize education with the multi-model GenAI tool google gemini? A journey towards effective policy making. J Adv Educ Philos.

[33] Glover E (2024). Grok: what we know about Elon Musk's AI chatbot. BuiltIn.

[34] Spataro J (2023). Introducing Microsoft 365 Copilot – your copilot for work. Official Microsoft Blog.

[35] Hernandez J, Frallicciardi A, Nadir N, Gothard MD, Ahmed RA (2020). Development of a Simulation Scenario Evaluation Tool (SSET): modified delphi study. BMJ Simul Technol Enhanc Learn.

[36] Mondal H, Mondal S (2024). Artificial intelligence-generated content needs a human oversight. Indian J Dermatol.

[37] Fang X, Che S, Mao M, Zhang H, Zhao M, Zhao X (2024). Bias of AI-generated content: an examination of news produced by large language models. Sci Rep.

[38] Meskó B (2023). Prompt engineering as an important emerging skill for medical professionals: tutorial. J Med Internet Res.

[39] Denny P, Leinonen J, Prather J, Luxton-Reilly A, Amarouche T, Becker BA, Reeves BN (2024). Prompt problems: a new programming exercise for the generative AI era. https://doi.org/10.1145/3626252.3630909.

