# Supplementary material for: Evaluating AI-Generated Geriatric Case Studies for Interprofessional Education: Systematic Analysis Across 5 Platforms
Source: JMIR Med Educ. 2026 Jan 30;12:e83085. doi: 10.2196/83085 (PMC12905562; doi:10.2196/83085)
Supplement: Multimedia Appendix 1 [file mededu_v12i1e83085_app1.docx]

**Reviewer Assignment for the AI in Geriatric Simulation Education**

Below are 50 case studies generated by the five generative AI platforms (e.g., ChatGPT, Claude, Co-Pilot, Gemini, and Grok). The cases are listed in alphabetical order and are blinded to which platform generated each case.

Contents

[Mrs. Ruth Adams 2](#_Toc184887033)

[Mrs. Isabella Bianchi 6](#_Toc184887034)

[Mrs. Evelyn Chen 8](#_Toc184887035)

[Mr. Arthur Chen 11](#_Toc184887036)

[Ms. Alice Dubois 13](#_Toc184887037)

[Mr. Walter Freeman 14](#_Toc184887038)

[Mr. Harold Green 18](#_Toc184887039)

[Mr. Samuel Greenberg 19](#_Toc184887040)

[Mr. George Hawkins 22](#_Toc184887041)

[Mrs. Emily Huang 25](#_Toc184887042)

[Mrs. Eleanor Jefferson 28](#_Toc184887043)

[Mr. Harold Jenkins 30](#_Toc184887044)

[Mrs. Barbara Jensen 33](#_Toc184887045)

[Mr. Henry Johnson 35](#_Toc184887046)

[Mr. Samuel Johnson 38](#_Toc184887047)

[Mr. Robert Johnson 42](#_Toc184887048)

[Mr. David Lee 43](#_Toc184887049)

[Mr. William “Bill” Martinez 45](#_Toc184887050)

[Mrs. Gloria Martinez 48](#_Toc184887051)

[Mrs. Li Mei 50](#_Toc184887052)

[Mrs. Gwendolyn Moss 52](#_Toc184887053)

[Mrs. Agnes Müller 55](#_Toc184887054)

[Mr. William "Bill" O'Connell 57](#_Toc184887055)

[Mr. Johnathan "Jack" O'Connor 58](#_Toc184887056)

[Mr. Samuel O'Reilly 61](#_Toc184887057)

[Mrs. Doris Patel 62](#_Toc184887058)

[Mrs. Lila Patel 66](#_Toc184887059)

[Mrs. Anna Patel 69](#_Toc184887060)

[Mr. Abdul Rahman 71](#_Toc184887061)

[Mr. Michael Roberts 71](#_Toc184887062)

[Mrs. Amelia Rodriguez 73](#_Toc184887063)

[Mrs. Anna Rodriguez 76](#_Toc184887064)

[Mrs. Elena Rodriguez 80](#_Toc184887065)

[Mrs. Gloria Rodriguez 81](#_Toc184887066)

[Ms. Maria Sanchez 82](#_Toc184887067)

[Mrs. Emily Smith 84](#_Toc184887068)

[Mrs. Irene Smith 86](#_Toc184887069)

[Mr. John Stevens 87](#_Toc184887070)

[Mr. Haruto Tanaka 91](#_Toc184887071)

[Mr. Leonard Thomas 93](#_Toc184887072)

[Mr. Harold Thompson 94](#_Toc184887073)

[Mrs. Eleanor Thompson 97](#_Toc184887074)

[Mr. Edward Thompson 100](#_Toc184887075)

[Mr. James "Jim" Thompson 102](#_Toc184887076)

[Mrs. Patricia “Pat” Thompson 104](#_Toc184887077)

[Mr. Samuel “Sam” Wilson 107](#_Toc184887078)

[Mrs. Clara Winston 110](#_Toc184887079)

[Mrs. Eleanor Wright 113](#_Toc184887080)

[Mrs. Dorothy Wu 114](#_Toc184887081)

[Mr. Takeshi Yamamoto 116](#_Toc184887082)

# Mrs. Ruth Adams

Patient Profile:

Name: Ruth Adams

Age: 79 years old

Gender: Female

Ethnicity: African American

Marital Status: Married for 55 years

Living Situation: Lives with her husband (85 years old) in their own home

Occupation: Retired schoolteacher

Insurance: Medicare

Primary Language: English

Advance Directive: Yes (Do Not Resuscitate - DNR)

Chief Complaint:

Mrs. Adams is brought to the outpatient clinic by her husband and adult daughter for a routine follow-up visit. Her family expresses concern over her worsening mobility, increasing dependence on her husband for daily activities, and recent episodes of incontinence. Her husband, who is also in declining health, admits to feeling overwhelmed with caregiving responsibilities.

Past Medical History:

Advanced Parkinson’s Disease (diagnosed 8 years ago)

Congestive Heart Failure (CHF, diagnosed 5 years ago)

Type 2 Diabetes Mellitus (diagnosed 15 years ago)

Osteoporosis (diagnosed 6 years ago)

Chronic Kidney Disease (Stage 3)

History of Urinary Tract Infections (UTIs)

Depression (diagnosed 2 years ago)

Current Medications:

Carbidopa-Levodopa 25/100 mg, three times daily

Furosemide 20 mg once daily

Metoprolol 50 mg twice daily

Insulin glargine 10 units at bedtime

Calcium + Vitamin D supplements daily

Sertraline 50 mg once daily

Acetaminophen 650 mg as needed for pain

Social History:

Mrs. Adams lives with her husband, who is her primary caregiver. They have two adult children, but only one daughter lives locally and helps occasionally.

Mrs. Adams requires assistance with most activities of daily living (ADLs) such as bathing, dressing, and transferring from bed to chair. She uses a walker but needs help with stairs and getting out of the car.

Mrs. Adams was active in her church community but has become more homebound over the past year.

She experiences anxiety about being a burden to her husband and often feels sad but denies suicidal thoughts.

Functional Status:

Mrs. Adams is dependent on her husband for most ADLs, including dressing, bathing, and toileting.

She has fallen twice in the last three months, although without injury.

Her Parkinson’s disease has progressed to the point where she experiences frequent tremors and difficulty initiating movement.

Her CHF is stable, but she reports increasing fatigue and occasional shortness of breath when climbing stairs or walking longer distances.

Nutrition:

Mrs. Adams has a reduced appetite and has unintentionally lost 10 pounds in the last six months. She relies on her husband for meal preparation, which he finds challenging, leading to inconsistent and unbalanced meals.

Her diabetes is managed with insulin, but her blood glucose levels fluctuate, particularly when her meals are delayed.

Review of Systems:

General: Weight loss, fatigue, generalized weakness

Cardiovascular: Occasional shortness of breath, mild edema in lower extremities

Respiratory: No cough or wheezing

Gastrointestinal: Decreased appetite, no nausea or vomiting

Genitourinary: Recent episodes of urinary incontinence, history of recurrent UTIs

Musculoskeletal: Stiffness, joint pain, reduced mobility due to Parkinson’s and osteoporosis

Neurological: Tremors, bradykinesia, postural instability, no recent falls

Psychological: Feels sad and overwhelmed, anxiety about caregiving burden on her husband

Physical Examination Findings:

General: Thin, frail appearance, appears fatigued

Vital signs:

Blood pressure: 140/90 mmHg

Pulse: 76 bpm, regular

Respiratory rate: 18 breaths/min

Oxygen saturation: 97% on room air

Temperature: 98.4°F

Cardiovascular: Mild peripheral edema in lower extremities, no jugular venous distention (JVD)

Lungs: Clear to auscultation bilaterally

Abdomen: Soft, non-tender

Neurological: Significant resting tremor in the right hand, cogwheel rigidity in both arms, reduced speed in motor activities

Musculoskeletal: Kyphosis, generalized muscle weakness, unsteady gait

Skin: Dry, thin skin with no pressure sores

Lab Results:

Complete Blood Count (CBC): Mild anemia (Hemoglobin 11.5 g/dL)

Basic Metabolic Panel (BMP): Slightly elevated creatinine (1.6 mg/dL), elevated potassium (5.2 mEq/L)

HbA1c: 7.5%

Urinalysis: Positive for white blood cells (indicative of UTI), no glucose

Echocardiogram: Left ventricular ejection fraction (LVEF) 45%

Interdisciplinary Learning Objectives:

Medical Students:

Conduct a comprehensive geriatric assessment, focusing on Mrs. Adams’ Parkinson’s disease, CHF, diabetes, and recurrent UTIs.

Develop a plan for managing her complex chronic conditions, particularly her declining mobility and incontinence.

Assess for possible medication side effects contributing to her symptoms, including polypharmacy concerns.

Evaluate her nutritional status and develop a plan to address her unintentional weight loss.

Nursing Students:

Perform a functional assessment to evaluate Mrs. Adams' current ability to perform ADLs and recommend appropriate interventions.

Provide education on fall prevention, skin care to avoid pressure ulcers, and managing incontinence.

Monitor fluid balance and discuss strategies for managing her CHF and recurrent UTIs.

Develop a care plan that addresses the caregiver burden her husband is experiencing, including respite care or home health support.

Social Work Students:

Conduct a psychosocial assessment to explore the emotional and social impact of caregiving on Mrs. Adams’ husband.

Explore community resources for caregiver support, such as respite services, adult day care, and in-home care options.

Discuss advance care planning and Mrs. Adams' end-of-life preferences with her and her family.

Address potential mental health concerns, including depression and anxiety, in both Mrs. Adams and her husband.

Discussion Questions:

What are the key challenges in managing Mrs. Adams’ Parkinson’s disease alongside her other chronic conditions? How can the interdisciplinary team address these challenges?

What strategies could be implemented to reduce the caregiver burden experienced by Mrs. Adams' husband? How can the team collaborate to support him?

How would you manage Mrs. Adams’ incontinence and prevent future UTIs while considering her physical limitations and mobility issues?

What nutrition interventions can be made to improve Mrs. Adams' dietary intake, and how would you involve her husband in these plans?

How should the team address Mrs. Adams' psychological and social needs, particularly her feelings of being a burden?

Simulation Objectives:

Develop an interdisciplinary care plan that focuses on both Mrs. Adams’ physical health and her husband’s caregiver burden.

Practice communication strategies for engaging with families of elderly patients in shared decision-making and care planning.

Address the psychosocial components of geriatric care, including depression, caregiver strain, and end-of-life planning.

This case emphasizes the challenges of managing multiple chronic illnesses in older adults, while also focusing on caregiver burden, an essential consideration for interdisciplinary health teams.

# Mrs. Isabella Bianchi

Background:

Patient: Mrs. Isabella BianchiAge: 88 yearsGender: FemaleEthnicity: Italian-AmericanMarital Status: WidowedLiving Situation: Recently transitioned from her longtime home to a senior living community due to increased need for assistance with daily activities. 
Medical History:

- Heart Failure: NYHA Class II, managed with medications but has episodes of fluid retention.
- Osteoporosis: With history of a wrist fracture from a fall 2 years ago.
- Chronic Constipation: Worsened by lack of mobility and medication for heart failure.
- Glaucoma: Stable, managed with eye drops.

Social History:

- Former opera singer, still loves listening to operas and classical music.
- Non-smoker, enjoys a small glass of red wine with dinner when allowed by her diet.
- Allergic to sulfa drugs.

Current Presentation:Mrs. Bianchi's daughter, who visits frequently, has noticed her mother seems more withdrawn and less interested in activities she once enjoyed. She has also lost weight unintentionally, about 10 pounds over the past 3 months, and has had several episodes where she forgot to take her heart medications, leading to mild exacerbations of her heart failure symptoms. 

Assessment:

- Vitals: BP: 125/70 mmHg, HR: 75 bpm, RR: 18 breaths/min with mild exertion, Temp: 97.8°F, O2 Sat: 94% on room air, weight loss noted.
- General Appearance: Thin, with signs of recent weight loss, appears somewhat disengaged but lights up when talking about music.
- Mental Status: Montreal Cognitive Assessment (MoCA) score of 23/30, showing mild cognitive impairment with deficits in executive function.
- Physical Exam:
- Cardiovascular: Slight pitting edema in ankles, normal heart sounds with occasional irregular rhythm.
- Musculoskeletal: Reduced bone density on recent DEXA scan, cautious mobility due to fear of falling.
- Gastrointestinal: Abdominal examination reveals mild distension, likely due to constipation.

Medications:

- Furosemide for heart failure
- Beta-blocker for heart rate control
- Potassium supplements (due to diuretic use)
- Bisphosphonates for osteoporosis
- Timolol eye drops for glaucoma

Psychosocial Factors:

- Struggles with the transition to senior living, feeling a loss of autonomy and identity.
- Misses performing and social interaction from her opera community.

Interdisciplinary Learning Objectives:

- Medical Students:
- Manage complex medication regimen for multiple chronic conditions, focusing on interactions and side effects.
- Investigate causes of weight loss in the elderly, considering both medical and psychological factors.
- Nursing Students:
- Implement and teach strategies for medication adherence in patients with mild cognitive impairment.
- Develop a care plan for preventing falls and managing chronic constipation.
- Social Work Students:
- Address the emotional impact of transitioning to assisted living, focusing on preserving dignity and a sense of purpose.
- Explore community resources or programs that could engage Mrs. Bianchi's interest in music, potentially improving her mental health.
- Interdisciplinary Goals:
- Enhance Mrs. Bianchi's quality of life by balancing her medical needs with her personal interests and psychological well-being.
- Coordinate a care plan that includes physical activity tailored to her condition, dietary adjustments for heart health and bone density, and social engagement to combat isolation.

Simulation Scenario:

- Setting: A home visit at the senior living community followed by a multidisciplinary team meeting at the clinic.
- Tasks:
- Assess living conditions for safety and fall risk, suggesting modifications.
- Review and possibly simplify her medication regimen for better adherence and reduced side effects.
- Engage her in a discussion about her hobbies, particularly music, to integrate into her daily routine for emotional health.
- Plan for dietary consultation to address weight loss while considering her heart failure diet restrictions.

Simulation Outcome:

Students should recognize the need for increased social interaction through music therapy or community opera appreciation groups, reassess her heart failure management to prevent episodes of decompensation, and introduce fiber-rich diet or gentle laxatives for constipation, all while ensuring her autonomy is respected in decision-making processes.

# Mrs. Evelyn Chen

Patient Information

- Name: Evelyn Chen
- Age: 79
- Gender: Female
- Marital Status: Married (55 years)
- Living Situation: Lives with husband in a retirement community

Chief Complaint

"I've been feeling very tired lately, and I think I might have an infection. My husband says I've been acting strangely too."

History of Present Illness

Mrs. Chen has been experiencing increasing fatigue over the past two weeks. She reports low-grade fever, decreased appetite, and burning sensation when urinating. Her husband notes that she has been more confused than usual, especially in the evenings, and has had difficulty sleeping at night.

Past Medical History

- Rheumatoid Arthritis (diagnosed 20 years ago)
- Osteoporosis
- Hypothyroidism
- Gastroesophageal Reflux Disease (GERD)
- Cataracts (surgery 2 years ago)

Medications

- Methotrexate 15 mg weekly
- Prednisone 5 mg daily
- Folic acid 1 mg daily
- Alendronate 70 mg weekly
- Levothyroxine 75 mcg daily
- Omeprazole 20 mg daily
- Calcium + Vitamin D supplement daily

Allergies

- Penicillin (hives)

Social History

- Retired librarian
- Lives with husband who has early-stage Alzheimer's disease
- Two adult children living in different states
- Active in local senior center and book club
- No smoking history, occasional glass of wine with dinner
- Uses a cane for ambulation due to arthritis

Review of Systems

- General: Fatigue, low-grade fever, decreased appetite
- Genitourinary: Dysuria, increased urinary frequency
- Neurological: Confusion, especially in the evenings
- Musculoskeletal: Chronic joint pain and stiffness
- Psychiatric: Irritability, sleep disturbances
- Gastrointestinal: Occasional heartburn

Physical Examination

- Vital Signs:
- BP: 118/72 mmHg
- HR: 92 bpm
- RR: 18/min
- Temp: 37.8°C (100°F)
- O2 Sat: 97% on room air
- General: Appears tired, slightly confused
- Neurological: Alert but disoriented to time, person and place intact
- Musculoskeletal: Swelling and tenderness in multiple joints, especially hands and knees
- Skin: No rashes, but skin appears dry
- Abdomen: Soft, mild suprapubic tenderness on palpation

Laboratory and Imaging Results

- Complete Blood Count (CBC):
- WBC: 12,500/µL (elevated)
- Hemoglobin: 10.8 g/dL (low)
- Basic Metabolic Panel (BMP):
- Sodium: 133 mEq/L (slightly low)
- Creatinine: 1.3 mg/dL (mildly elevated)
- Urinalysis:
- Leukocyte esterase: Positive
- Nitrites: Positive
- WBC: >20/hpf
- ESR: 45 mm/hr (elevated)
- CRP: 3.5 mg/dL (elevated)
- TSH: 5.8 mIU/L (slightly elevated)

Assessment

- Urinary Tract Infection with associated delirium
- Rheumatoid Arthritis flare
- Anemia of chronic disease
- Hypothyroidism, undertreated
- Possible medication-induced cognitive effects (e.g., from prednisone)
- Caregiver stress (caring for husband with early Alzheimer's)
- Falls risk due to multiple factors (arthritis, medication side effects, delirium)

Plan

- Start appropriate antibiotic therapy for UTI based on local guidelines and patient's allergies
- Rheumatology consultation for management of RA flare
- Adjust levothyroxine dose based on TSH results
- Geriatric psychiatry consultation for evaluation of delirium and cognitive status
- Review and potentially adjust medications, especially considering cognitive effects
- Occupational therapy and physical therapy evaluations
- Social work consultation for caregiver support and community resource connection
- Nutrition consultation for appetite issues and overall health
- Consider home health services for short-term support during recovery

Simulation Learning Objectives

- Medical Students:
- Diagnose and manage urinary tract infection in the elderly
- Recognize and manage delirium, differentiating it from dementia
- Understand the complexities of managing autoimmune diseases in older adults
- Develop a comprehensive care plan considering multiple chronic conditions and acute illness
- Nursing Students:
- Implement delirium prevention and management strategies
- Conduct pain assessment and management in a patient with chronic arthritis
- Develop and implement a fall prevention plan
- Educate patient and caregiver on medication management and signs of infection
- Social Work Students:
- Assess caregiver stress and provide appropriate support and resources
- Evaluate the need for additional home services or respite care
- Explore community resources for patients with chronic diseases and their caregivers
- Address advance care planning considerations
- Interdisciplinary Objectives:
- Practice effective communication with patients experiencing delirium
- Collaborate to develop a person-centered care plan addressing medical, functional, and psychosocial needs
- Recognize and address polypharmacy issues in older adults with multiple chronic conditions
- Develop strategies for supporting both the patient and their caregiver spouse

Simulation Scenario Progression

- Initial Assessment: Each discipline conducts their respective assessments in the emergency department or upon admission.
- Team Huddle: Interdisciplinary team meets to discuss findings and develop an initial care plan.
- Acute Management: Simulate management of UTI and delirium, including non-pharmacological interventions.
- Medication Review: Team collaborates on medication reconciliation, considering potential cognitive effects and drug interactions.
- Family Meeting: Simulate a meeting with Mrs. Chen (when more lucid) and her husband to discuss diagnosis, treatment plan, and support needs.
- Discharge Planning: Team develops a comprehensive plan for transition of care, including medication management, follow-up appointments, and caregiver support.
- Follow-up: Simulate a post-discharge follow-up appointment to assess resolution of acute issues and manage chronic conditions.

This case study provides multiple learning opportunities for students across healthcare disciplines, focusing on the complex care needs of an older adult with multiple chronic conditions, an acute infection leading to delirium, and the added dimension of being a caregiver to a spouse with cognitive impairment.

# Mr. Arthur Chen

Patient Demographics:

- Age: 76
- Gender: Male
- Marital Status: Married, wife with moderate dementia
- Living Situation: Lives with wife in a one-story home
- Children: Two sons, one lives locally, one lives overseas
- Medical History: Type 2 Diabetes, Coronary Artery Disease (s/p CABG 10 years prior), Peripheral Artery Disease, Depression, history of smoking

Presenting Complaint:

Mr. Chen is referred to the home health agency after a recent hospitalization for uncontrolled blood sugar and worsening peripheral artery disease. He reports feeling overwhelmed with caring for his wife, whose dementia has progressed significantly. He is experiencing increasing fatigue, pain in his legs, and difficulty managing his own medications and diet.

Physical Examination:

- Vital Signs: BP 130/80 mmHg, HR 72 bpm, RR 16, Temp 98.9 F
- General Appearance: Tired appearing, lower extremities are pale and cool to touch
- Cardiovascular: Weak pedal pulses bilaterally
- Neurological: Alert and oriented, reports feeling down and hopeless
- Other: Small ulcer on right heel

Social History:

- Mr. Chen was a chef before retirement.
- He is the primary caregiver for his wife, and he is reluctant to consider assisted living.
- He has limited mobility due to his peripheral artery disease and relies on his son for transportation.
- He is struggling to cope with the changes in his wife's condition and his own declining health.

Learning Objectives:

- Caregiver Burden: Students will need to assess Mr. Chen's caregiver burden and identify strategies to provide support and respite care.
- Chronic Disease Management: Students will need to address Mr. Chen's multiple chronic conditions, including diabetes, coronary artery disease, and peripheral artery disease.
- Medication Adherence: Students will need to assess and address barriers to medication adherence.
- Wound Care: Students will need to develop a plan for managing Mr. Chen's foot ulcer.
- Mental Health: Students will need to assess and address Mr. Chen's depression.
- Advance Care Planning: Students will need to initiate a discussion about advance care planning with Mr. Chen and his family.
- Cultural Considerations: Students will need to be sensitive to Mr. Chen's cultural background and beliefs when providing care.

Simulation Activities:

- Home Visit: Students can conduct a simulated home visit to assess Mr. Chen's living environment and provide education on disease management and self-care.
- Caregiver Support Group: Students can role-play a caregiver support group to provide Mr. Chen with an opportunity to share his experiences and connect with others.
- Interprofessional Case Conference: Students from different disciplines can participate in a case conference to discuss Mr. Chen's care plan and coordinate services.
- Medication Management Simulation: Students can practice medication reconciliation and develop strategies to improve adherence.
- Advance Care Planning Role-Play: Students can role-play a conversation with Mr. Chen and his family about advance care planning.

Assessment:

- Students can be assessed on their ability to:
- Assess caregiver burden and provide support
- Develop a comprehensive care plan for multiple chronic conditions
- Address barriers to medication adherence and self-care
- Provide culturally sensitive care
- Initiate a discussion about advance care planning
- Collaborate effectively with other disciplines

This case study highlights the complex challenges faced by many older adults with chronic conditions and caregiver responsibilities. It provides students with an opportunity to develop the knowledge, skills, and attitudes necessary to provide high-quality, person-centered care to this vulnerable population.

# Ms. Alice Dubois

Patient Demographics:

- Age: 75
- Gender: Female
- Marital Status: Never married, lives alone
- Living Situation: Independent living apartment in a retirement community
- Children: None
- Medical History: Osteoarthritis, Hypertension, Anxiety, Depression, Social Anxiety Disorder

Presenting Complaint:

Ms. Dubois is referred to the social work department by the retirement community staff due to concerns about her increasing social isolation and withdrawal. She rarely leaves her apartment, has stopped participating in community activities, and has declined offers for social interaction. She reports feeling lonely and sad but is resistant to seeking help or engaging with others.

Physical Examination:

- Vital Signs: BP 120/80 mmHg, HR 68 bpm, RR 16, Temp 98.0 F
- General Appearance: Well-groomed, appears anxious and withdrawn
- Musculoskeletal: Limited range of motion in hands and knees due to osteoarthritis

Social History:

- Ms. Dubois was a successful accountant before retiring.
- She has a history of anxiety and depression, which worsened after her retirement.
- She has always been introverted and has a limited social support network.
- She reports feeling overwhelmed by the thought of socializing and fears being judged or rejected by others.
- She is struggling to find meaning and purpose in her life after retirement.

Learning Objectives:

- Mental Health in Older Adults: Students will need to assess Ms. Dubois's anxiety, depression, and social anxiety and develop a plan for intervention and treatment.
- Social Isolation and Loneliness: Students will need to address Ms. Dubois's social isolation and explore strategies to promote social connection and engagement.
- Adjustment to Retirement: Students will need to understand the challenges of adjusting to retirement and help Ms. Dubois develop coping mechanisms and find new sources of meaning and purpose.
- Motivational Interviewing and Therapeutic Communication: Students will need to practice effective communication strategies to build rapport with Ms. Dubois and encourage her to engage in treatment and social activities.
- Community Resources: Students will need to connect Ms. Dubois with appropriate community resources, such as support groups, therapy services, and social activities within the retirement community.

Simulation Activities:

- Therapeutic Communication Role-Play: Students can practice communication techniques to build trust and rapport with Ms. Dubois and address her anxiety and resistance to help.
- Motivational Interviewing Simulation: Students can use motivational interviewing techniques to encourage Ms. Dubois to explore social opportunities and engage in treatment.
- Community Resource Navigation: Students can research and present information on relevant community resources within the retirement community and surrounding area.
- Interprofessional Case Conference: Students from different disciplines can collaborate to discuss Ms. Dubois's care plan and coordinate services.
- Support Group Role-Play: Students can simulate a support group meeting for older adults with social anxiety to provide Ms. Dubois with a safe space to connect with others.

Assessment:

- Students can be assessed on their ability to:
- Assess and address mental health concerns in older adults
- Develop strategies to combat social isolation and loneliness
- Facilitate adjustment to retirement and promote meaning and purpose
- Utilize effective communication and motivational interviewing techniques
- Connect older adults with appropriate community resources

This case study focuses on the often overlooked issue of mental health and social isolation in older adults. It provides students with an opportunity to develop the skills necessary to address these challenges and promote the well-being of older adults living in retirement communities.

# Mr. Walter Freeman

Patient Information

- Name: Walter Freeman
- Age: 86
- Gender: Male
- Marital Status: Widowed (5 years ago)
- Living Situation: Lives alone in a rural farmhouse

Chief Complaint

"I fell off my tractor yesterday and now my chest hurts. I don't want to be here; I need to get back to my farm."

History of Present Illness

Mr. Freeman was brought to the emergency department by his neighbor after falling from his tractor yesterday afternoon. He reports severe pain in his chest and right hip. He initially refused to come to the hospital but relented when he found it difficult to breathe. Mr. Freeman is adamant about returning home as soon as possible, stating he has animals to care for.

Past Medical History

- Chronic Obstructive Pulmonary Disease (COPD)
- Atrial Fibrillation
- Osteoarthritis
- Hearing Impairment
- History of Skin Cancer (Basal Cell Carcinoma, removed 2 years ago)

Medications

- Tiotropium inhaler 18 mcg daily
- Albuterol inhaler as needed
- Apixaban 5 mg twice daily
- Metoprolol 25 mg twice daily
- Acetaminophen 500 mg as needed for pain

Allergies

- No known drug allergies

Social History

- Retired farmer, but still actively manages his small farm
- Widowed 5 years ago; wife died of stroke
- One adult son who lives 3 hours away
- No history of smoking or alcohol use
- Fiercely independent, has refused previous suggestions to move closer to town
- Limited social interactions, primarily with neighbors and at local farmers' market

Review of Systems

- General: Fatigue, no recent weight changes
- Respiratory: Increased shortness of breath, chronic cough
- Cardiovascular: No palpitations, occasional lightheadedness
- Musculoskeletal: Chronic joint pain, new severe pain in chest and right hip
- Neurological: No headaches or dizziness
- Skin: Multiple actinic keratoses on face and arms

Physical Examination

- Vital Signs:
- BP: 158/90 mmHg
- HR: 92 bpm, irregular
- RR: 24/min
- Temp: 37.0°C (98.6°F)
- O2 Sat: 88% on room air, 94% on 2L nasal cannula
- General: Alert, oriented, appears uncomfortable and slightly short of breath
- Respiratory: Decreased breath sounds bilaterally, with crackles at bases
- Cardiovascular: Irregular rhythm, no murmurs
- Musculoskeletal:
- Tenderness and ecchymosis over right lateral chest wall
- Pain with passive movement of right hip
- Multiple joint deformities consistent with osteoarthritis
- Neurological: Grossly intact, limited by pain
- Skin: Multiple actinic keratoses on sun-exposed areas

Laboratory and Imaging Results

- Complete Blood Count (CBC):
- WBC: 11,000/µL (slightly elevated)
- Hemoglobin: 13.2 g/dL
- Platelets: 180,000/µL
- Basic Metabolic Panel:
- Creatinine: 1.3 mg/dL (mildly elevated)
- Otherwise within normal limits
- Troponin: Negative
- BNP: 180 pg/mL (mildly elevated)
- Chest X-ray: Multiple rib fractures on right side, no pneumothorax
- Hip X-ray: No acute fracture
- ECG: Atrial fibrillation with controlled ventricular response

Assessment

- Multiple rib fractures
- COPD exacerbation
- Atrial Fibrillation
- Osteoarthritis
- Possible hip contusion
- Chronic kidney disease, Stage 3
- Fall risk
- Caregiver absence
- Potential self-neglect

Plan

- Admit for pain management and monitoring of respiratory status
- Pain management with consideration of risks in elderly patient (avoid NSAIDs, cautious opioid use)
- Pulmonology consultation for management of COPD exacerbation
- Cardiology consultation for management of atrial fibrillation in the context of trauma
- Physical therapy and occupational therapy evaluations
- Social work consultation for:
- Home safety evaluation
- Assessment of capacity for independent living
- Discussion of long-term care options
- Geriatric consultation for comprehensive assessment and care planning
- Advance care planning discussion
- Consider anticoagulation bridge given need to hold apixaban peri-injury

Simulation Learning Objectives

- Medical Students:
- Manage acute trauma in an elderly patient with multiple comorbidities
- Recognize and address COPD exacerbation in the context of chest wall injury
- Develop a comprehensive care plan balancing acute needs with chronic disease management
- Navigate the complexities of anticoagulation management in a patient with recent trauma
- Nursing Students:
- Implement appropriate pain management strategies for elderly patients with rib fractures
- Conduct fall risk assessment and implement prevention strategies
- Develop and implement a plan to prevent hospital-acquired complications in a frail elderly patient
- Recognize signs of self-neglect and initiate appropriate interventions
- Social Work Students:
- Assess capacity for independent living in a fiercely independent elderly patient
- Navigate ethical considerations related to autonomy versus safety
- Explore options for increased support or alternative living arrangements in a rural setting
- Facilitate discussions about advance care planning and long-term care preferences
- Interdisciplinary Objectives:
- Practice effective communication with patients who have sensory impairments
- Collaborate to develop a person-centered care plan that addresses medical, functional, and psychosocial needs
- Recognize and address potential elder abuse or self-neglect
- Develop strategies for supporting safe discharge planning for a high-risk rural-dwelling elder

Simulation Scenario Progression

- Initial Assessment: Each discipline conducts their respective assessments, with a focus on acute trauma management and underlying chronic conditions.
- Team Huddle: Interdisciplinary team meets to discuss findings and develop an initial care plan.
- Acute Management: Simulate management of pain and respiratory status, including medication administration and monitoring.
- Family Meeting: Conduct a family meeting (including son via video call) to discuss hospital course, concerns about independent living, and future care needs.
- Capacity Assessment: Team collaborates to assess Mr. Freeman's capacity for medical decision-making and independent living.
- Care Planning: Develop a comprehensive care plan that addresses acute medical needs, chronic disease management, and safety concerns.
- Ethical Discussion: Navigate ethical considerations related to respecting patient autonomy versus ensuring safety.
- Discharge Planning: Team develops a plan for safe discharge, including home modifications, support services, and follow-up care.
- Follow-up: Simulate a follow-up visit to assess recovery, medication management, and adjustment to any new support services.

This case study provides multiple learning opportunities for students across healthcare disciplines, focusing on the complex care needs of an older adult living independently in a rural setting, dealing with acute trauma superimposed on multiple chronic conditions, and navigating the balance between autonomy and safety.

# Mr. Harold Green

Patient Profile:

- Name: Harold Green
- Age: 78
- Gender: Male
- Ethnicity: Caucasian
- Marital Status: Married
- Living Situation: Lives with spouse in a two-story home
- Insurance: Medicare and private supplemental insurance

Medical History:

- Chronic Obstructive Pulmonary Disease (COPD) (diagnosed 8 years ago)
- Congestive Heart Failure (diagnosed 5 years ago)
- Benign Prostatic Hyperplasia (BPH) (diagnosed 10 years ago)
- Hyperlipidemia (diagnosed 15 years ago)
- Moderate Dementia (recently diagnosed)

Current Medications:

- Albuterol inhaler PRN
- Furosemide 40 mg daily
- Simvastatin 20 mg daily
- Tamsulosin 0.4 mg daily
- Donepezil 5 mg daily

Chief Complaint: Mr. Green presents with increasing shortness of breath, difficulty sleeping due to nocturnal dyspnea, and recent episodes of urinary incontinence.

Social History:

- Retired engineer
- Has two adult children who live in the same city and visit weekly
- Enjoys gardening and reading but has reduced activity due to health issues
- Active in a local senior center but has had decreased participation recently

Physical Examination Findings:

- BP: 140/85 mmHg
- HR: 95 bpm
- RR: 22 breaths/min
- Temp: 98.2°F
- Weight: 180 lbs, Height: 5'10"
- General: Appears tired, mild respiratory distress
- HEENT: No acute distress, mild peripheral edema
- Cardiovascular: Regular rate and rhythm, mild jugular venous distension
- Respiratory: Bilateral wheezing, crackles at lung bases
- Abdomen: Mild suprapubic tenderness
- Neurological: Oriented to person but not to place or time, moderate cognitive impairment

Simulation Objectives:

- Medical Students:
- Conduct a thorough evaluation of COPD and heart failure management.
- Develop an appropriate medication regimen and titration plan.
- Recognize and address symptoms of dementia and urinary incontinence.
- Nursing Students:
- Perform a comprehensive physical assessment and monitor vital signs.
- Develop a care plan focusing on respiratory and cardiac function, sleep hygiene, and incontinence management.
- Educate the patient and family on disease management and symptom monitoring.
- Social Work Students:
- Assess the impact of Mr. Green's health on his daily life and family dynamics.
- Identify resources and support services to assist with his care at home.
- Collaborate with the healthcare team to address Mr. Green's social and psychological needs.

Discussion Points:

- Managing multiple chronic conditions in elderly patients.
- Importance of family involvement and support in geriatric care.
- Strategies for improving quality of life and reducing hospital readmissions.
- Role of interdisciplinary teamwork in providing comprehensive care.
- This case study can be used to foster collaboration among students from different health disciplines, emphasizing the need for a holistic approach to geriatric care.

# Mr. Samuel Greenberg

Background:

Patient: Mr. Samuel Greenberg Age: 82 years Gender: Male Ethnicity: Jewish Marital Status: Widowed Living Situation: Lives in a retirement community apartment, with a son who lives nearby but works long hours. 

Medical History:

- Parkinson's Disease: Diagnosed 5 years ago, currently on levodopa-carbidopa, experiencing wearing off phenomena.
- Benign Prostatic Hyperplasia (BPH): Managed with tamsulosin.
- Hyperlipidemia: Controlled with statins.
- Chronic Kidney Disease (Stage 3): Monitored through diet and hydration advice.
- Hearing Loss: Uses a hearing aid.

Social History:

- Retired accountant, once very active in financial literacy programs for the community.
- Never smoked, occasional social drinking.
- No known drug allergies.

Current Presentation:Mr. Greenberg's son brought him to the clinic after noticing an increase in his tremor, more frequent falls, and confusion, particularly in the evening. He also reports changes in his sleep pattern and appetite.

Assessment:

- Vitals: BP: 138/82 mmHg, HR: 68 bpm, RR: 16 breaths/min, Temp: 97.8°F, O2 Sat: 96% on room air.
- General Appearance: Shuffling gait, stooped posture, moderate tremor in hands at rest.
- Mental Status: Sometimes confused, especially after sundowning. MMSE score of 24/30, showing issues with attention and calculation.
- Physical Exam:
- Neurological: Rigidity in limbs, bradykinesia, positive cogwheel rigidity.
- Gastrointestinal: Soft, non-tender abdomen, normal bowel sounds.
- Cardiovascular: Normal heart sounds, no edema noted.
- Urological: No acute urinary retention signs, but reports nocturia.

Medications:

- Levodopa-carbidopa
- Tamsulosin
- Atorvastatin
- Multivitamin

Psychosocial Factors:

Feeling isolated despite living in a community, misses his wife deeply.

Worried about losing his independence as his Parkinson's progresses.

Occasionally feels like a burden to his son.

Interdisciplinary Learning Objectives:

- Medical Students:
- Understand the management of Parkinson's disease, including dosage adjustments and potential for polypharmacy issues.
- Recognize the signs of Parkinson's disease complications like psychosis and motor fluctuations.
- Nursing Students:
- Develop strategies for managing Parkinson's symptoms, including mobility aids and fall prevention.
- Educate on medication timing and the importance of regular follow-ups.
- Social Work Students:
- Assess the patient's social support network and explore community resources for elderly with mobility issues.
- Address psychological aspects like grief, isolation, and fears of losing autonomy.
- Interdisciplinary Goals:
- Coordinate care to optimize medication for Parkinson's while minimizing side effects.
- Plan for potential transitions to higher care settings as the disease progresses.

Simulation Scenario:

- Setting: Outpatient clinic visit followed by a home visit.

- Tasks:
- Review current medication regimen and adjust for Parkinson's symptoms.
- Assess home for safety (e.g., removing trip hazards, installing grab bars).
- Discuss dietary needs considering his kidney disease and medication interactions.
- Plan mental health support, possibly involving occupational therapy for daily living skills.

Simulation Outcome:

Students should identify the need for a comprehensive review of his Parkinson's treatment, possibly introducing a dopamine agonist or COMT inhibitor. They should also consider non-pharmacological interventions like physical therapy to improve gait and balance. Discussions about future care arrangements, including possible assisted living or in-home care, should be initiated to prepare for potential future needs.

This case study provides a multifaceted scenario where students must consider the interplay of multiple health conditions, medication management, psychological well-being, and social support in geriatric care.

# Mr. George Hawkins

Patient Information

- Name: George Hawkins
- Age: 82
- Gender: Male
- Marital Status: Married (55 years)
- Living Situation: Lives with wife in a single-story apartment in an assisted living facility

Chief Complaint

"I'm so confused lately. My wife says I'm not myself, and I keep forgetting things."

History of Present Illness

Mr. Hawkins' wife reports a gradual decline in his cognitive function over the past 6 months. She notes he has become increasingly forgetful, sometimes getting lost in familiar places, and has difficulty managing his medications. In the past week, he has become more agitated, especially in the evenings, and has had episodes of visual hallucinations.

Past Medical History

- Parkinson's Disease (diagnosed 5 years ago)
- Coronary Artery Disease (CAD) with history of myocardial infarction 10 years ago
- Chronic Obstructive Pulmonary Disease (COPD)
- Benign Prostatic Hyperplasia (BPH)
- Depression (diagnosed 2 years ago)

Medications

- Levodopa/Carbidopa 25/100 mg three times daily
- Atorvastatin 40 mg daily
- Tiotropium inhaler 18 mcg daily
- Tamsulosin 0.4 mg daily
- Sertraline 50 mg daily
- Aspirin 81 mg daily

Allergies

- No known drug allergies

Social History

- Retired mechanical engineer
- Married for 55 years; wife is primary caregiver
- Two adult children who live in nearby cities
- Stopped driving 6 months ago due to Parkinson's progression
- Former smoker (quit 15 years ago), no alcohol use

Review of Systems

- General: Fatigue, unintentional weight loss of 5 kg in 3 months
- Neurological: Tremors, stiffness, balance issues (Parkinson's symptoms)
- Psychiatric: Confusion, agitation, visual hallucinations
- Respiratory: Chronic cough, occasional shortness of breath
- Cardiovascular: No chest pain or palpitations
- Genitourinary: Urinary frequency and urgency
- Gastrointestinal: Poor appetite, constipation

Physical Examination

- Vital Signs:
- BP: 128/76 mmHg
- HR: 68 bpm
- RR: 20/min
- Temp: 36.8°C (98.2°F)
- O2 Sat: 94% on room air
- General: Alert but disoriented to time and place, appears older than stated age
- Neurological:
- Resting tremor in both hands
- Bradykinesia and rigidity noted
- Shuffling gait, stooped posture
- MMSE (Mini-Mental State Examination) score: 18/30
- Cardiovascular: Regular rate and rhythm, S4 gallop present
- Respiratory: Decreased breath sounds bilaterally, occasional wheezes
- Abdomen: Soft, non-tender, no masses
- Skin: Dry, poor turgor

Laboratory and Imaging Results

- Complete Blood Count (CBC):
- Hemoglobin: 11.2 g/dL (slightly low)
- WBC: Within normal limits
- Basic Metabolic Panel (BMP):
- Sodium: 133 mEq/L (slightly low)
- Creatinine: 1.3 mg/dL (mildly elevated)
- Thyroid Stimulating Hormone (TSH): Within normal limits
- Vitamin B12: 180 pg/mL (low)
- CT scan of the brain: Moderate cortical atrophy, no acute changes

Assessment

- Probable Lewy Body Dementia (given Parkinson's history, cognitive decline, and visual hallucinations)
- Parkinson's Disease
- Delirium, likely multifactorial (medication side effects, possible UTI, electrolyte imbalance)
- Coronary Artery Disease
- COPD
- Depression
- Vitamin B12 deficiency
- Malnutrition risk

Plan

- Neurology consultation for evaluation and management of probable Lewy Body Dementia
- Adjust Parkinson's medications (potential contribution to hallucinations)
- Urine analysis and culture to rule out UTI
- Start Vitamin B12 supplementation
- Psychiatry consultation for management of depression and behavioral symptoms
- Occupational therapy and physical therapy evaluations
- Nutrition consultation for weight loss and malnutrition risk
- Consider sleep study to evaluate for sleep disorders
- Social work consultation for caregiver support and long-term care planning
- Medication reconciliation and consider deprescribing where appropriate

Simulation Learning Objectives

- Medical Students:
- Differentiate between delirium and dementia
- Diagnose and manage Lewy Body Dementia
- Recognize and address polypharmacy in elderly patients
- Develop a comprehensive care plan for a patient with multiple chronic conditions
- Nursing Students:
- Conduct a thorough geriatric nursing assessment, including cognitive and functional assessments
- Implement strategies to manage behavioral symptoms in dementia
- Develop and implement a fall prevention plan
- Educate caregivers on managing dementia-related behaviors
- Social Work Students:
- Assess caregiver stress and provide appropriate support and resources
- Evaluate the need for additional home services or higher level of care
- Discuss advance care planning with patient and family
- Identify community resources for patients with dementia and their caregivers
- Interdisciplinary Objectives:
- Practice effective communication in complex care situations
- Collaborate to develop a person-centered care plan addressing medical, functional, and psychosocial needs
- Recognize and address ethical issues in geriatric and dementia care (e.g., capacity for decision-making, safety vs. autonomy)
- Develop strategies for supporting and educating family caregivers

Simulation Scenario Progression

- Initial Assessment: Each discipline conducts their respective assessments.
- Team Huddle: Interdisciplinary team meets to discuss findings and develop an initial care plan.
- Family Meeting: Simulate a meeting with Mr. Hawkins and his wife to discuss diagnosis, prognosis, and care options.
- Medication Review: Team collaborates on medication reconciliation and deprescribing plan.
- Behavioral Management: Simulate managing an episode of confusion and agitation.
- Discharge Planning: Team develops a comprehensive plan for ongoing care and support at home or in a higher level of care facility.
- Follow-up: Simulate a follow-up appointment 1 month later to assess effectiveness of interventions and adjust the care plan.

This case study provides multiple learning opportunities for students across healthcare disciplines, focusing on the complex care needs of an older adult with cognitive impairment, multiple chronic conditions, and significant caregiver involvement.

# Mrs. Emily Huang

Patient Profile:

Name: Emily Huang

Age: 88 years old

Gender: Female

Ethnicity: Chinese American

Marital Status: Widowed for 12 years

Living Situation: Lives in an assisted living facility

Occupation: Retired university librarian

Insurance: Medicare with supplemental private insurance

Primary Language: English (fluent), Mandarin (native language)

Advance Directive: No formal advance directive in place, but her family reports she prefers not to have aggressive treatments.

Chief Complaint:

Mrs. Huang is brought to the primary care clinic by her daughter for an evaluation of worsening confusion, frequent falls, and decreased appetite. The staff at the assisted living facility has noted that she has been more forgetful, less engaged in activities, and is experiencing difficulty managing her medications. She has fallen twice in the past month, although no injuries were reported.

Past Medical History:

Hypertension (diagnosed 25 years ago)

Type 2 Diabetes Mellitus (diagnosed 15 years ago)

Osteoarthritis (diagnosed 10 years ago)

Mild cognitive impairment (diagnosed 2 years ago, with recent worsening)

Depression (diagnosed 5 years ago after the death of her husband)

Recurrent urinary tract infections (UTIs)

Frailty syndrome (recently identified)

Current Medications:

Metformin 500 mg twice daily (for diabetes)

Lisinopril 10 mg once daily (for hypertension)

Acetaminophen 500 mg as needed for pain (up to 4 times per day)

Sertraline 50 mg once daily (for depression)

Vitamin D supplement daily (for bone health)

Calcium carbonate 500 mg twice daily

Nitrofurantoin 50 mg once daily (prophylactic for UTIs)

Multivitamin daily

Social History:

Mrs. Huang is originally from China and immigrated to the U.S. 50 years ago. She has maintained strong cultural ties with her Chinese community, participating in social events at her assisted living facility when she was more mobile and engaged.

She has two adult children: a daughter who lives nearby and visits regularly and a son who lives out of state and visits occasionally.

Mrs. Huang speaks both English and Mandarin, though she increasingly prefers Mandarin as her cognitive function declines.

She enjoys reading, but her cognitive decline has reduced her ability to focus on books or conversations.

Functional Status:

Mrs. Huang requires assistance with most ADLs, including bathing, dressing, and medication management.

She uses a walker but is at high risk for falls due to weakness, poor balance, and occasional dizziness.

Her appetite has decreased in recent months, and she has unintentionally lost 7 pounds over the last six months.

She reports fatigue and mild pain in her knees and back due to osteoarthritis.

Review of Systems:

General: Weight loss, fatigue, frailty

Cardiovascular: No chest pain, controlled hypertension, no palpitations

Respiratory: No shortness of breath, occasional mild cough

Gastrointestinal: Decreased appetite, no nausea or vomiting, occasional constipation

Genitourinary: Frequent urination, history of recurrent UTIs, currently asymptomatic

Musculoskeletal: Chronic knee and back pain from osteoarthritis, weakness, unsteady gait

Neurological: Worsening confusion, difficulty concentrating, increased forgetfulness, no recent stroke or seizures

Psychological: Feels sad occasionally, mild withdrawal from social activities, but denies suicidal thoughts

Physical Examination Findings:

General: Frail, thin, appears older than her stated age

Vital signs:

Blood pressure: 135/75 mmHg

Pulse: 72 bpm, regular

Respiratory rate: 16 breaths/min

Oxygen saturation: 97% on room air

Temperature: 98.0°F

Cardiovascular: Regular heart sounds, no murmurs

Lungs: Clear to auscultation bilaterally

Abdomen: Soft, non-tender

Musculoskeletal: Mild tenderness in knees, limited range of motion, slight kyphosis

Neurological: Oriented to person but not to time or place, unable to complete Mini-Mental Status Exam (MMSE) without assistance

Skin: Thin, dry skin with mild bruising on arms from recent falls

Lab Results:

Complete Blood Count (CBC): Mild anemia (Hemoglobin 11.2 g/dL)

Basic Metabolic Panel (BMP): Creatinine 1.4 mg/dL, glucose 130 mg/dL (fasting), normal electrolytes

HbA1c: 7.2% (slightly above target)

Urinalysis: Negative for infection

Vitamin D level: Low (18 ng/mL)

Interdisciplinary Learning Objectives:

Medical Students:

Conduct a comprehensive geriatric assessment, focusing on cognitive decline, frailty, and polypharmacy.

Develop a plan to manage Mrs. Huang’s diabetes, hypertension, and osteoarthritis while minimizing the risk of falls and medication-related side effects.

Evaluate the potential causes of her recurrent UTIs and explore non-pharmacological prevention strategies.

Consider interventions to improve her nutritional status and address her weight loss and frailty.

Nursing Students:

Perform a functional assessment to evaluate Mrs. Huang’s mobility, balance, and risk for falls, and suggest interventions to reduce fall risk.

Educate Mrs. Huang and her family on safe medication management and potential side effects, including the risk of polypharmacy in elderly patients.

Monitor her nutritional intake and develop a care plan to improve her appetite and overall nutrition, including assessing for potential causes of decreased appetite.

Implement a skin care regimen to prevent bruising and pressure sores due to frailty and thin skin.

Social Work Students:

Conduct a psychosocial assessment of Mrs. Huang’s mental and emotional well-being, considering her isolation, cognitive decline, and loss of independence.

Explore cultural factors in Mrs. Huang’s care, such as language preferences and her connection to the Chinese community.

Discuss potential caregiver strain on her daughter and explore support services, such as home health aides or community resources.

Facilitate discussions about advance care planning and decision-making, including the possibility of completing a formal advance directive.

Discussion Questions:

How can the interdisciplinary team address Mrs. Huang’s polypharmacy and minimize the risk of adverse drug reactions or side effects?

What strategies can be implemented to reduce Mrs. Huang’s fall risk and improve her mobility, given her frailty and cognitive decline?

How should the team address Mrs. Huang’s nutritional needs and unintentional weight loss, particularly in the context of her decreased appetite and frailty?

How can the healthcare team incorporate Mrs. Huang’s cultural preferences and family dynamics into her care plan, particularly regarding her cognitive decline and decision-making?

How would the team approach a conversation with Mrs. Huang and her family about advance care planning, especially considering her cognitive decline and lack of a formal directive?

Simulation Objectives:

Develop an interdisciplinary care plan that addresses polypharmacy, frailty, cognitive decline, and fall prevention in an elderly patient.

Practice culturally competent care, taking into account language preferences and the role of family in decision-making.

Collaborate on strategies to improve Mrs. Huang’s nutrition, mobility, and overall quality of life while considering her cognitive and physical limitations.

Facilitate discussions with the patient and her family regarding advance care planning, including addressing cognitive decline and long-term care options.

This case study focuses on the challenges of managing polypharmacy, frailty, and cognitive decline in elderly patients. It encourages interdisciplinary collaboration, cultural sensitivity, and the integration of medical, nursing, and social work interventions to improve Mrs. Huang’s overall quality of life.

# Mrs. Eleanor Jefferson

Patient Profile:

- Name: Eleanor Jefferson
- Age: 82
- Gender: Female
- Ethnicity: African American
- Marital Status: Widowed
- Living Situation: Lives alone in a single-story home
- Insurance: Medicare

Medical History:

- Hypertension (diagnosed 10 years ago)
- Type 2 Diabetes Mellitus (diagnosed 15 years ago)
- Osteoarthritis in both knees
- Mild cognitive impairment (recently diagnosed)
- History of stroke (2 years ago)
- Recent episode of pneumonia (6 months ago)

Current Medications:

- Metformin 500 mg BID
- Lisinopril 20 mg daily
- Acetaminophen 500 mg TID for pain
- Aspirin 81 mg daily
- Insulin glargine 10 units at bedtime

Chief Complaint: Mrs. Jefferson presents with increasing difficulty managing her daily activities, frequent falls, and confusion regarding her medication regimen.

Social History:

- Retired schoolteacher
- Has one daughter who lives out of state and visits monthly
- Active in her church community but has decreased participation recently
- Relies on neighbors for help with groceries and transportation

Physical Examination Findings:

- BP: 150/90 mmHg
- HR: 88 bpm
- RR: 18 breaths/min
- Temp: 98.6°F
- Weight: 150 lbs, Height: 5'4"
- General: Alert but confused at times, frail appearance
- HEENT: No acute distress
- Cardiovascular: Regular rate and rhythm, no murmurs
- Respiratory: Clear to auscultation bilaterally
- Musculoskeletal: Decreased range of motion in knees, pain on palpation
- Neurological: Mild cognitive impairment, positive Romberg test

Simulation Objectives:

- Medical Students:
- Develop a comprehensive geriatric assessment.
- Create a management plan for chronic conditions.
- Recognize and address polypharmacy issues.
- Nursing Students:
- Conduct a thorough physical examination.
- Develop a care plan focusing on fall prevention and medication management.
- Educate the patient on safe medication practices and lifestyle modifications.
- Social Work Students:
- Assess the patient's social support system and community resources.
- Develop an intervention plan to improve the patient's social engagement and access to services.
- Collaborate with healthcare providers to address the patient's holistic needs.

Discussion Points:

- How to balance medical management with quality of life in elderly patients.
- Strategies to prevent falls and enhance home safety.
- Importance of interdisciplinary care and effective communication among healthcare providers.
- Addressing the psychosocial aspects of aging and chronic illness.
- This case study can serve as a basis for simulation scenarios, encouraging students to work together across disciplines to provide comprehensive care for elderly patients.

# Mr. Harold Jenkins

Patient Information

Name: Harold Jenkins

Age: 78

Gender: Male

Marital Status: Married (53 years)

Living Situation: Lives with wife in a two-story home

Chief Complaint

"I'm here because my wife insisted. I've been having these dizzy spells and I fell last week. But I'm more worried about my memory - I feel like I'm losing my mind."

History of Present Illness

Mr. Jenkins reports experiencing intermittent dizziness and lightheadedness over the past month, particularly when standing up quickly. He had a fall last week but didn't sustain any injuries. His wife notes that he's become increasingly forgetful over the past six months, often misplacing items and forgetting appointments. Mr. Jenkins admits to feeling anxious about these changes and has been having trouble sleeping.

Past Medical History

Parkinson's Disease (diagnosed 3 years ago)

Hypertension

Benign Prostatic Hyperplasia (BPH)

History of Transient Ischemic Attack (TIA) 2 years ago

Osteoarthritis (knees and hips)

Medications

Levodopa/Carbidopa 25/100 mg three times daily

Ropinirole 2 mg three times daily

Lisinopril 20 mg daily

Tamsulosin 0.4 mg daily

Aspirin 81 mg daily

Acetaminophen 500 mg as needed for pain

Allergies

Penicillin (rash)

Social History

Retired high school principal

Lives with wife who is primary caregiver

Two adult children living in nearby cities

Stopped driving 6 months ago due to family's concerns

Former smoker (quit 20 years ago), occasional glass of wine with dinner

Used to enjoy golfing and woodworking but has given up these hobbies recently

Review of Systems

General: Fatigue, unintentional weight loss of 5 lbs in 1 month

Neurological: Dizziness, memory problems, tremor, stiffness

Cardiovascular: No chest pain, occasional palpitations

Genitourinary: Urinary frequency and urgency, nocturia

Psychiatric: Anxiety, sleep disturbances

Musculoskeletal: Chronic knee and hip pain, increased stiffness

Physical Examination

Vital Signs:

BP: 110/70 mmHg (supine), 90/60 mmHg (standing)

HR: 68 bpm (supine), 88 bpm (standing)

RR: 16/min

Temp: 36.6°C (97.9°F)

O2 Sat: 98% on room air

General: Alert, oriented to person and place but not to time, appears anxious

Neurological:

Resting tremor in both hands

Bradykinesia and rigidity noted

Postural instability on pull test

MMSE (Mini-Mental State Examination) score: 22/30

Cardiovascular: Regular rate and rhythm, no murmurs

Musculoskeletal: Decreased range of motion in knees and hips, stable gait with shuffling

Skin: No rashes or lesions

Laboratory and Imaging Results

Complete Blood Count (CBC): Within normal limits

Comprehensive Metabolic Panel:

Sodium: 133 mEq/L (slightly low)

Otherwise within normal limits

Thyroid Stimulating Hormone (TSH): Within normal limits

Vitamin B12: 190 pg/mL (low)

MRI brain: Moderate cortical atrophy, no acute infarcts, small vessel ischemic changes

Assessment

Orthostatic hypotension

Mild Cognitive Impairment (MCI) vs. early dementia

Parkinson's Disease with worsening symptoms

Falls risk

Anxiety

Vitamin B12 deficiency

Possible depression

Osteoarthritis

Sleep disturbance

Plan

Neurology consultation for management of Parkinson's Disease and cognitive assessment

Adjust antihypertensive medication due to orthostatic hypotension

Start Vitamin B12 supplementation

Occupational therapy and physical therapy evaluations for fall prevention and home safety

Cognitive assessment and possible neuropsychological testing

Geriatric psychiatry consultation for evaluation of anxiety and possible depression

Sleep study to evaluate for sleep disorders

Social work consultation for caregiver support and community resource connection

Medication reconciliation and consider deprescribing where appropriate

Discuss advance care planning

Simulation Learning Objectives

Medical Students:

Evaluate and manage orthostatic hypotension in the context of Parkinson's Disease

Differentiate between Mild Cognitive Impairment (MCI) and early dementia

Develop a comprehensive care plan addressing multiple geriatric syndromes

Navigate the complexities of medication management in Parkinson's Disease

Nursing Students:

Conduct a thorough fall risk assessment and implement prevention strategies

Develop and implement a plan for medication management and adherence

Recognize and address sleep disturbances in elderly patients

Provide education and support for patients and caregivers dealing with progressive neurological conditions

Social Work Students:

Assess caregiver stress and provide appropriate support and resources

Evaluate the need for additional home services or community support

Facilitate discussions about advance care planning and long-term care preferences

Address the psychosocial impact of declining cognitive and physical function

Interdisciplinary Objectives:

Practice effective communication with patients who have cognitive impairment

Collaborate to develop a person-centered care plan that addresses medical, functional, and psychosocial needs

Recognize and address the impact of multiple co-existing conditions on patient care

Develop strategies for supporting both the patient and their caregiver in the context of a progressive neurological condition

Simulation Scenario Progression

Initial Assessment: Each discipline conducts their respective assessments, focusing on fall risk, cognitive status, and Parkinson's symptoms.

Team Huddle: Interdisciplinary team meets to discuss findings and develop an initial care plan.

Medication Review: Simulate a pharmacy consultation to address polypharmacy and medication adjustment.

Family Meeting: Conduct a family meeting with Mr. Jenkins and his wife to discuss findings, concerns, and care planning.

Therapeutic Intervention: Role-play a physical therapy session focusing on fall prevention and a psychiatry consultation addressing anxiety and sleep issues.

Cognitive Assessment: Simulate a detailed cognitive assessment and discussion of results with the patient and family.

Care Planning: Develop a comprehensive care plan that addresses medical needs, safety concerns, and psychosocial support.

Advance Care Planning: Facilitate a discussion about future care preferences and document advance directives.

Follow-up: Simulate a follow-up visit to assess the effectiveness of interventions, medication changes, and adjustment to new support services.

This case study provides multiple learning opportunities for students across healthcare disciplines, focusing on the complex care needs of an older adult dealing with Parkinson's Disease, cognitive decline, and multiple geriatric syndromes. It emphasizes the importance of interdisciplinary collaboration in addressing the multifaceted needs of geriatric patients with progressive neurological conditions.

# Mrs. Barbara Jensen

Patient Profile:

- Name: Barbara Jensen
- Age: 83
- Gender: Female
- Ethnicity: Caucasian
- Marital Status: Widowed
- Living Situation: Lives alone in a single-story house
- Insurance: Medicare

Medical History:

- Alzheimer's disease (diagnosed 4 years ago)
- Type 2 Diabetes Mellitus (diagnosed 15 years ago)
- Hypertension (diagnosed 20 years ago)
- Osteoarthritis in both hips (diagnosed 10 years ago)
- Recent history of urinary tract infections (last 3 months)
- Current Medications:
- Metformin 500 mg BID
- Donepezil 10 mg daily
- Lisinopril 20 mg daily
- Acetaminophen 500 mg TID for pain
- Insulin glargine 10 units at bedtime

Chief Complaint: Mrs. Jensen presents with worsening memory issues, difficulty managing her diabetes, and increased hip pain affecting her mobility.

Social History:

- Retired librarian
- Has two daughters, one lives nearby and visits daily, the other lives out of state
- Enjoys reading and knitting but has reduced activity due to cognitive decline
- Participates in a local senior center but has decreased involvement recently

Physical Examination Findings:

- BP: 150/90 mmHg
- HR: 85 bpm
- RR: 18 breaths/min
- Temp: 98.6°F
- Weight: 145 lbs, Height: 5'4"
- General: Alert but occasionally confused, frail appearance
- HEENT: No acute distress
- Cardiovascular: Regular rate and rhythm, no murmurs
- Respiratory: Clear to auscultation bilaterally
- Musculoskeletal: Decreased range of motion in hips, pain on palpation
- Neurological: Moderate cognitive impairment, follows simple commands

Simulation Objectives:

- Medical Students:
- Develop a comprehensive management plan for Alzheimer's disease and its complications.
- Address issues related to polypharmacy and medication adherence.
- Evaluate and manage diabetes and hypertension in the context of other comorbidities.
- Nursing Students:
- Conduct a thorough physical assessment, focusing on pain management and mobility.
- Develop a care plan to address fall prevention, medication management, and blood sugar control.
- Educate the patient and family on safe medication practices, lifestyle modifications, and diabetes management.
- Social Work Students:
- Assess the impact of cognitive decline on Mrs. Jensen's daily life and social interactions.
- Identify community resources and support systems for patients with Alzheimer's disease.
- Collaborate with the healthcare team to address Mrs. Jensen's psychosocial needs and improve her quality of life.

Discussion Points:

- Balancing the management of multiple chronic conditions in elderly patients.
- Strategies to prevent falls and enhance home safety.
- Importance of interdisciplinary care and effective communication among healthcare providers.
- Addressing the psychosocial aspects of aging and chronic illness.
- This case study provides an opportunity for students from different health disciplines to collaborate and develop a comprehensive, patient-centered care plan for elderly patients with multiple chronic conditions.

# Mr. Henry Johnson

Patient Profile:

Name: Henry Johnson

Age: 78 years old

Gender: Male

Ethnicity: African American

Marital Status: Widower (wife passed away 5 years ago)

Living Situation: Lives alone in an apartment

Occupation: Retired truck driver

Insurance: Medicare and Medicaid

Primary Language: English

Advance Directive: Yes, prefers comfort measures and no life-sustaining treatments

Chief Complaint:

Mr. Johnson is admitted to the hospital with worsening, uncontrolled pain due to metastatic prostate cancer. He has been using increasing doses of opioids at home for pain relief, but his pain is now interfering with his ability to eat, sleep, and walk. His daughter, who lives nearby, is concerned that he is becoming more confused and forgetful, and she fears that he is overmedicating himself. Mr. Johnson expresses that he is tired of the pain and is considering stopping all aggressive treatments, including chemotherapy.

Past Medical History:

Metastatic prostate cancer (diagnosed 3 years ago)

Chronic lower back pain (due to bone metastases)

Opioid dependence (due to long-term use for cancer-related pain)

Hypertension (diagnosed 20 years ago)

Type 2 Diabetes Mellitus (diagnosed 15 years ago)

Depression (diagnosed 5 years ago after the death of his wife)

History of smoking (40 pack-years, quit 10 years ago)

Current Medications:

Oxycodone 10 mg every 4 hours as needed (for pain)

Acetaminophen 650 mg every 6 hours as needed (for pain)

Lisinopril 10 mg once daily (for hypertension)

Metformin 500 mg twice daily (for diabetes)

Duloxetine 30 mg once daily (for depression)

Insulin glargine 10 units nightly (for diabetes)

Ondansetron 4 mg every 8 hours as needed (for nausea)

Gabapentin 300 mg twice daily (for nerve pain)

Social History:

Mr. Johnson was married for 45 years before his wife passed away. He lives alone in an apartment but has daily visits from his daughter, who helps him manage his medications and meals.

He used to enjoy fishing and woodworking, but his pain and fatigue have limited these activities. He spends most of his time watching TV or sleeping.

He quit smoking 10 years ago after his prostate cancer diagnosis but was a heavy smoker for most of his life.

His daughter is his primary caregiver, but she is struggling with her father’s increasing dependence and is unsure how to manage his worsening pain.

Functional Status:

Mr. Johnson is mostly independent with ADLs, but his pain limits his ability to walk, bathe, and dress. He requires assistance with meal preparation and medication management.

He uses a walker to get around, but due to his pain and weakness, he has become mostly sedentary.

His appetite has decreased, and he has unintentionally lost 15 pounds over the past 6 months.

He expresses feelings of sadness and hopelessness about his condition and fears becoming a burden on his daughter.

Review of Systems:

General: Weight loss, fatigue, worsening pain, nausea, decreased appetite

Cardiovascular: No chest pain or palpitations, controlled hypertension

Respiratory: Mild shortness of breath with exertion, no cough

Gastrointestinal: Poor appetite, occasional constipation, nausea (controlled with ondansetron)

Genitourinary: Urinary incontinence, controlled with adult briefs

Musculoskeletal: Severe lower back pain radiating to the legs, weakness, limited mobility

Neurological: Increased confusion, occasional forgetfulness, no recent falls

Psychological: Feels sad, hopeless, considers stopping treatment for cancer

Physical Examination Findings:

General: Frail, appears tired and in distress, grimaces with movement

Vital signs:

Blood pressure: 130/80 mmHg

Pulse: 88 bpm, regular

Respiratory rate: 20 breaths/min

Oxygen saturation: 96% on room air

Temperature: 98.0°F

Cardiovascular: Regular heart sounds, no murmurs

Lungs: Clear to auscultation bilaterally

Abdomen: Soft, non-tender, no organomegaly

Musculoskeletal: Tenderness over lower back and pelvis, limited range of motion due to pain

Neurological: Alert but slow to respond, oriented to person but confused about date and time

Skin: No rashes, mild bruising over arms from frequent needle sticks

Lab Results:

Complete Blood Count (CBC): Mild anemia (Hemoglobin 10.5 g/dL)

Basic Metabolic Panel (BMP): Creatinine 1.3 mg/dL, glucose 140 mg/dL (fasting), electrolytes normal

Liver Function Tests (LFTs): Mildly elevated AST and ALT

Prostate-Specific Antigen (PSA): Elevated (consistent with metastatic disease progression)

HbA1c: 7.8% (slightly elevated)

Interdisciplinary Learning Objectives:

Medical Students:

Assess Mr. Johnson’s pain management regimen, including his opioid use, and develop a plan to manage his pain while addressing potential opioid dependence.

Review his current cancer treatment and discuss the options for palliative care or hospice, considering his expressed desire to stop aggressive treatments.

Address his nutritional status, including weight loss and decreased appetite, and explore strategies to improve his nutrition and prevent further decline.

Discuss the management of his comorbid conditions (diabetes, hypertension) in the context of his advanced cancer.

Nursing Students:

Perform a comprehensive assessment of Mr. Johnson’s functional status, focusing on his ability to perform ADLs and manage pain.

Provide education on opioid safety, including strategies to prevent overdose and manage side effects like constipation and confusion.

Develop a care plan that addresses his pain management, mobility limitations, and risk for pressure ulcers due to his sedentary lifestyle.

Monitor his mental health and provide emotional support, considering his feelings of hopelessness and depression.

Social Work Students:

Conduct a psychosocial assessment of Mr. Johnson’s emotional well-being and explore his thoughts about ending aggressive cancer treatments.

Facilitate discussions with Mr. Johnson and his family about the transition to palliative care or hospice, ensuring that his wishes are respected.

Explore caregiver strain and provide support for his daughter, who may be experiencing burnout from managing her father’s care.

Discuss advance care planning and ensure that Mr. Johnson’s advance directive is up to date, including his preferences for end-of-life care.

Discussion Questions:

How can the healthcare team manage Mr. Johnson’s pain effectively while minimizing the risks associated with opioid dependence and side effects?

What are the key considerations when discussing the transition to palliative care or hospice for Mr. Johnson, and how can the team support him in making this decision?

How should the interdisciplinary team address Mr. Johnson’s emotional distress and depression, particularly his fears of becoming a burden to his daughter?

What strategies can be implemented to support Mr. Johnson’s daughter, who is his primary caregiver, and prevent caregiver burnout?

How can the healthcare team ensure that Mr. Johnson’s advance directive and end-of-life wishes are respected, and what role does the family play in this process?

Simulation Objectives:

Develop an interdisciplinary care plan that addresses Mr. Johnson’s pain management, emotional well-being, and potential transition to palliative care or hospice.

Practice sensitive communication strategies for discussing end-of-life care, including addressing Mr. Johnson’s fears and ensuring his wishes are respected.

Explore strategies to prevent opioid overdose and manage the side effects of long-term opioid use, particularly in elderly patients with cancer.

Support the patient’s family, especially the primary caregiver, by offering resources to manage caregiver strain and ensure their involvement in care decisions.

This case study emphasizes the challenges of managing chronic pain, opioid dependence, and the transition to palliative care in a patient with advanced cancer. It encourages interdisciplinary collaboration to address the medical, psychological, and social aspects of care, including end-of-life decision-making and caregiver support.

# Mr. Samuel Johnson

Patient Information

- Name: Samuel Johnson
- Age: 84
- Gender: Male
- Marital Status: Widowed (1 year ago)
- Living Situation: Recently moved to an assisted living facility

Chief Complaint

"I don't know why I'm here. My daughter thinks I'm not eating well, but I'm fine. I just want to go home."

History of Present Illness

Mr. Johnson was brought to the emergency department by his daughter, who reports that her father has lost significant weight over the past three months. She became concerned after visiting him and finding little food in his apartment. Mr. Johnson appears resistant to being in the hospital and insists he's been eating fine. The daughter also mentions that her father has been increasingly forgetful and has missed several doctors' appointments.

Past Medical History

- Alzheimer's Disease (diagnosed 2 years ago)
- Coronary Artery Disease with history of CABG 10 years ago
- Type 2 Diabetes Mellitus
- Chronic Kidney Disease Stage 3
- History of Major Depressive Disorder

Medications

- Donepezil 10 mg daily
- Metformin 500 mg twice daily
- Atorvastatin 40 mg daily
- Aspirin 81 mg daily
- Lisinopril 10 mg daily
- Escitalopram 10 mg daily

Allergies

- Codeine (nausea and vomiting)

Social History

- Retired high school history teacher
- Widowed 1 year ago; wife died after a long battle with cancer
- Recently moved to an assisted living facility (3 months ago)
- One adult daughter who lives 30 minutes away
- Former smoker (quit 30 years ago), no alcohol use
- Used to enjoy gardening and reading, but has lost interest in these activities

Review of Systems

- General: Unintentional weight loss, fatigue
- Neurological: Increased forgetfulness, difficulty with daily tasks
- Psychiatric: Low mood, anhedonia
- Gastrointestinal: Poor appetite, no nausea or vomiting
- Cardiovascular: No chest pain or shortness of breath
- Endocrine: Polyuria, polydipsia

Physical Examination

- Vital Signs:
- BP: 130/70 mmHg
- HR: 72 bpm
- RR: 16/min
- Temp: 36.6°C (97.9°F)
- O2 Sat: 97% on room air
- General: Alert but disoriented, appears older than stated age, cachectic
- Neurological:
- MMSE (Mini-Mental State Examination) score: 19/30
- Clock-drawing test: Impaired
- Cardiovascular: Regular rate and rhythm, no murmurs
- Respiratory: Clear to auscultation bilaterally
- Abdomen: Soft, non-tender, no masses
- Skin: Dry, poor turgor, no pressure ulcers

Laboratory and Imaging Results

- Complete Blood Count (CBC):
- WBC: 5,500/µL
- Hemoglobin: 11.2 g/dL (low)
- MCV: 82 fL
- Basic Metabolic Panel (BMP):
- Sodium: 135 mEq/L
- Potassium: 4.2 mEq/L
- BUN: 28 mg/dL (elevated)
- Creatinine: 1.6 mg/dL (elevated)
- Glucose: 168 mg/dL (elevated)
- HbA1c: 7.8%
- Albumin: 3.0 g/dL (low)
- Vitamin B12: 180 pg/mL (low)
- Vitamin D: 18 ng/mL (deficient)
- TSH: Within normal limits

Assessment

- Alzheimer's Disease with moderate cognitive impairment
- Malnutrition
- Major Depressive Disorder, likely exacerbated by recent life changes
- Type 2 Diabetes Mellitus, suboptimal control
- Chronic Kidney Disease, stable
- Vitamin B12 deficiency
- Vitamin D deficiency
- Caregiver stress (daughter)
- Adjustment disorder related to recent move to assisted living

Plan

- Geriatric psychiatry consultation for evaluation of cognitive status and depression
- Nutrition consultation for malnutrition and diabetes management
- Adjust diabetes medications considering renal function
- Start Vitamin B12 and Vitamin D supplementation
- Occupational therapy evaluation for assessment of activities of daily living
- Social work consultation for:
- Evaluation of current living situation
- Caregiver support for daughter
- Advance care planning discussion
- Consider addition of memantine for Alzheimer's Disease
- Medication reconciliation and consider deprescribing where appropriate

Simulation Learning Objectives

- Medical Students:
- Conduct a comprehensive geriatric assessment
- Manage multiple chronic conditions in the context of cognitive impairment
- Recognize and address malnutrition in the elderly
- Develop a care plan that balances medical needs with patient autonomy
- Nursing Students:
- Implement strategies for managing patients with dementia in an acute care setting
- Conduct nutritional assessments and interventions
- Develop and implement a plan to prevent hospital-acquired complications in a frail elderly patient
- Practice patient-centered communication with cognitively impaired patients
- Social Work Students:
- Assess capacity for independent living and need for additional support services
- Provide support and resources for family caregivers
- Facilitate discussions about advance care planning
- Navigate ethical considerations related to patient autonomy and safety
- Interdisciplinary Objectives:
- Practice effective communication with patients who have cognitive impairment
- Collaborate to develop a person-centered care plan that addresses medical, functional, and psychosocial needs

Recognize and address ethical dilemmas in geriatric care (e.g., autonomy vs. safety)

Develop strategies for managing transitions of care in elderly patients with cognitive impairment

Simulation Scenario Progression

- Initial Assessment: Each discipline conducts their respective assessments, with a focus on managing Mr. Johnson's resistance to care.
- Team Huddle: Interdisciplinary team meets to discuss findings and develop an initial care plan.
- Family Meeting: Simulate a meeting with Mr. Johnson and his daughter to discuss findings, treatment options, and care planning.
- Capacity Assessment: Team collaborates to assess Mr. Johnson's capacity for medical decision-making and independent living.
- Care Planning: Develop a comprehensive care plan that balances medical needs, safety concerns, and respect for patient autonomy.
- Ethical Discussion: Address ethical considerations related to involuntary admission, capacity, and end-of-life planning.
- Discharge Planning: Team develops a plan for safe discharge, including medication management, follow-up care, and increased support services.
- Follow-up: Simulate a follow-up visit to assess adjustment to the care plan and address ongoing concerns.

This case study provides multiple learning opportunities for students across healthcare disciplines, focusing on the complex care needs of an older adult with cognitive impairment, multiple chronic conditions, and significant psychosocial factors impacting care.

# Mr. Robert Johnson

Patient Demographics:

- Age: 72
- Gender: Male
- Marital Status: Single, lives with long-term partner
- Living Situation: Lives in a suburban home with his partner who has multiple sclerosis
- Children: Two adult children from a previous marriage, limited contact
- Medical History: Alcohol Use Disorder, Early-stage Dementia, Peripheral Neuropathy, Hypertension

Presenting Complaint:

Mr. Johnson is brought to the emergency department by his partner's home health aide who found him confused and agitated. He has a history of falls and has become increasingly forgetful. He is also experiencing worsening pain in his feet and legs, which he attributes to his "old war injury." He admits to drinking heavily to manage his pain and cope with his partner's declining health.

Physical Examination:

- Vital Signs: BP 160/100 mmHg, HR 96 bpm, RR 18, Temp 99.0 F
- General Appearance: Unkempt, odor of alcohol, ataxic gait
- Neurological: Confusion, impaired memory, decreased sensation in lower extremities
- Gastrointestinal: Abdominal distention, tenderness to palpation

Social History:

- Mr. Johnson was a construction worker before retiring.
- He is a veteran and struggles with PTSD from his wartime experiences.
- He is the primary caregiver for his partner, who has progressive multiple sclerosis.
- He has a history of alcohol abuse and has been resistant to seeking treatment.
- He is socially isolated and has strained relationships with his children.

Learning Objectives:

- Substance Use Disorder in Older Adults: Students will need to assess Mr. Johnson's alcohol use and develop a plan for intervention and treatment.
- Cognitive Impairment and Dementia Care: Students will need to assess his cognitive function and address safety concerns related to his dementia.
- Chronic Pain Management: Students will need to address his chronic pain and explore alternative pain management strategies.
- Caregiver Burden and Complex Care Needs: Students will need to assess the caregiver burden on both Mr. Johnson and his partner and connect them with appropriate support services.
- Interprofessional Collaboration: Students from different disciplines will need to collaborate to develop a comprehensive care plan that addresses Mr. Johnson's complex physical, mental health, and social needs.
- Ethical Considerations: Students will need to address ethical dilemmas related to Mr. Johnson's autonomy, capacity for decision-making, and potential self-neglect.

Simulation Activities:

- Motivational Interviewing: Students can practice motivational interviewing techniques to encourage Mr. Johnson to engage in treatment for his alcohol use disorder.
- Pain Assessment and Management Simulation: Students can assess Mr. Johnson's pain and explore non-pharmacological pain management strategies.
- Home Safety Assessment: Students can conduct a home visit to assess safety risks and make recommendations for modifications.
- Caregiver Support Group Role-Play: Students can participate in a support group for caregivers of individuals with multiple sclerosis.
- Interprofessional Case Conference: Students from different disciplines can collaborate to develop a comprehensive care plan for Mr. Johnson and his partner.

Assessment:

- Students can be assessed on their ability to:
- Assess and address substance use disorder in older adults
- Manage cognitive impairment and dementia-related behaviors
- Develop a comprehensive pain management plan
- Address caregiver burden and complex care needs
- Collaborate effectively with other disciplines
- Address ethical considerations related to patient autonomy and safety

This case study provides a valuable opportunity for students to develop the skills necessary to address the complex challenges faced by older adults with substance use disorders, cognitive impairment, chronic pain, and caregiving responsibilities.

# Mr. David Lee

Patient Demographics:

- Age: 80
- Gender: Male
- Marital Status: Married
- Living Situation: Lives with wife in a multi-generational household with their daughter, son-in-law, and two grandchildren.
- Children: One daughter
- Medical History: Stroke (6 months prior with residual right-sided weakness and aphasia), Type 2 Diabetes, Hypertension, Early-stage Alzheimer's Disease

Presenting Complaint:

Mr. Lee is brought to the rehabilitation clinic by his family for a follow-up appointment. While he has made some progress in physical therapy, his family is concerned about his increasing frustration with communication difficulties due to aphasia. They also report that he has become more withdrawn and less interested in participating in family activities. His wife is struggling to manage his care needs along with her responsibilities for the grandchildren.

Physical Examination:

- Vital Signs: BP 130/80 mmHg, HR 76 bpm, RR 16, Temp 98.6 F
- General Appearance: Mild right-sided weakness, facial droop, difficulty with expressive language
- Neurological: Aphasia (expressive), reduced motor strength on right side, mild cognitive impairment
- Other: Evidence of poor wound healing on right foot

Social History:

- Mr. Lee was a successful businessman before his stroke.
- He is accustomed to being independent and providing for his family.
- He enjoys spending time with his grandchildren and attending cultural events.
- He is struggling to adjust to his physical limitations and communication difficulties.
- His family is supportive but experiencing stress related to his care needs and the demands of a multi-generational household.

Learning Objectives:

- Stroke Rehabilitation and Communication Challenges: Students will need to assess Mr. Lee's communication needs and develop strategies to improve his communication skills and social interaction.
- Cognitive Impairment and Alzheimer's Disease: Students will need to assess his cognitive function and provide education and support to his family about Alzheimer's Disease.
- Family Dynamics and Caregiver Support: Students will need to assess the impact of Mr. Lee's condition on his family and provide support and resources to address caregiver burden and family dynamics in a multi-generational household.
- Cultural Considerations: Students will need to be sensitive to Mr. Lee's cultural background and beliefs when providing care and communicating with his family.
- Interprofessional Collaboration: Students from different disciplines will need to collaborate to develop a comprehensive care plan that addresses Mr. Lee's physical, cognitive, and psychosocial needs.

Simulation Activities:

- Communication Strategies Role-Play: Students can practice communication techniques with a standardized patient portraying Mr. Lee to enhance their skills in interacting with individuals with aphasia.
- Family Meeting Simulation: Students can facilitate a family meeting to address concerns, provide education about Alzheimer's Disease, and develop strategies to support Mr. Lee and his caregivers.
- Interprofessional Case Conference: Students from different disciplines can collaborate to discuss Mr. Lee's care plan and coordinate services.
- Home Visit Simulation: Students can conduct a home visit to assess the home environment and make recommendations for modifications to support Mr. Lee's mobility and safety.

Assessment:

- Students can be assessed on their ability to:
- Assess and address communication challenges in stroke patients
- Understand and manage early-stage Alzheimer's Disease
- Provide support and resources to families and caregivers
- Demonstrate cultural sensitivity in caregiving
- Collaborate effectively with other disciplines

This case study highlights the complex interplay of physical, cognitive, and psychosocial factors that can affect stroke survivors and their families. It provides students with an opportunity to develop the knowledge, skills, and attitudes necessary to provide holistic, patient-centered care to this population in a culturally sensitive manner.

# Mr. William “Bill” Martinez

Patient Profile:

Name: William “Bill” Martinez

Age: 85 years old

Gender: Male

Ethnicity: Hispanic

Marital Status: Widowed for 10 years

Living Situation: Lives with his adult son, Robert, and daughter-in-law

Occupation: Retired construction worker

Insurance: Medicare and Medicaid

Primary Language: Bilingual (Spanish and English)

Advance Directive: No formal documentation; family reports verbal wishes

Chief Complaint:

Mr. Martinez is brought to the primary care clinic by his son, Robert, due to concerns about his increasing confusion, episodes of agitation, and difficulty managing daily activities. Robert expresses frustration with his father’s wandering behavior and verbal aggression, particularly in the evenings. He reports that the caregiving burden has strained his family’s relationship and finances.

Past Medical History:

Alzheimer’s Disease (diagnosed 3 years ago, moderate stage)

Hypertension (diagnosed 20 years ago)

Hyperlipidemia (diagnosed 15 years ago)

Gout (diagnosed 7 years ago)

History of stroke (2 years ago, no residual deficits)

Current Medications:

Donepezil 10 mg once daily

Atorvastatin 40 mg once daily

Lisinopril 20 mg once daily

Allopurinol 100 mg once daily

Aspirin 81 mg once daily

Melatonin 3 mg at bedtime (for sleep)

Social History:

Mr. Martinez was widowed 10 years ago and moved in with his son and daughter-in-law shortly after his Alzheimer’s diagnosis.

He is originally from Mexico and has lived in the U.S. for the past 50 years. He is fluent in both Spanish and English but reverts to Spanish when agitated.

His son works full-time, and his daughter-in-law provides most of his care during the day, though she is not trained in caregiving and expresses feeling overwhelmed.

Mr. Martinez enjoys listening to music but no longer engages in many of his former hobbies, like gardening or attending church, due to his cognitive decline.

Functional Status:

Mr. Martinez is no longer independent in his ADLs. He requires assistance with bathing, dressing, and toileting.

He has episodes of incontinence and forgets to take his medications, which are managed by his son.

He wanders around the house and has attempted to leave several times, particularly in the evening (suspected “sundowning”).

His memory has significantly worsened over the past year, and he sometimes does not recognize his son.

He is increasingly agitated and verbally aggressive, particularly when being assisted with personal care.

Review of Systems:

General: Weight stable, occasional fatigue

Cardiovascular: No chest pain, occasional mild headaches, no palpitations

Respiratory: No cough, no shortness of breath

Gastrointestinal: Regular bowel movements, no diarrhea or constipation

Genitourinary: Occasional incontinence, particularly at night

Musculoskeletal: History of gout with occasional flare-ups, no current joint pain

Neurological: Increased forgetfulness, disorientation, episodes of wandering, and verbal aggression; no recent strokes

Psychological: Agitation, anxiety in the evenings, verbally aggressive with caregivers, feelings of sadness

Physical Examination Findings:

General: Appears well-nourished, slightly disheveled in appearance

Vital signs:

Blood pressure: 150/85 mmHg

Pulse: 70 bpm, regular

Respiratory rate: 16 breaths/min

Oxygen saturation: 98% on room air

Temperature: 98.6°F

Cardiovascular: Regular heart sounds, no murmurs

Lungs: Clear to auscultation bilaterally

Abdomen: Soft, non-tender

Neurological: Oriented to person but not to time or place, unable to complete Mini-Mental Status Exam (MMSE) due to agitation

Musculoskeletal: No acute joint swelling or tenderness

Skin: No rashes or pressure ulcers

Lab Results:

Complete Blood Count (CBC): Normal

Basic Metabolic Panel (BMP): Normal

Lipid Panel: Elevated LDL (130 mg/dL)

Uric Acid Level: Mildly elevated (7.2 mg/dL)

Interdisciplinary Learning Objectives:

Medical Students:

Conduct a comprehensive assessment of Mr. Martinez’s cognitive and behavioral symptoms, focusing on the progression of Alzheimer’s disease.

Develop a pharmacological plan to manage Mr. Martinez’s hypertension, hyperlipidemia, and gout in the context of his Alzheimer’s.

Evaluate options for managing behavioral symptoms like agitation and wandering (non-pharmacological and pharmacological approaches).

Assess the risk of future cardiovascular events, considering his history of stroke and current cardiovascular health.

Nursing Students:

Perform a functional assessment to evaluate Mr. Martinez’s current level of independence with ADLs and develop strategies for providing safe care.

Educate the family on managing behaviors associated with Alzheimer’s, such as wandering, sundowning, and aggression.

Implement fall prevention strategies and monitor for signs of dehydration or malnutrition, given his cognitive impairment.

Assess the caregiving burden on his family, particularly his daughter-in-law, and discuss the potential for respite care.

Social Work Students:

Conduct a psychosocial assessment to evaluate the emotional, financial, and relational impact of caregiving on Mr. Martinez’s family.

Explore cultural factors in Mr. Martinez’s care, such as language preferences and family dynamics in caregiving.

Discuss community resources for caregiving support, such as adult day programs, Alzheimer’s support groups, and financial assistance.

Facilitate conversations about advance care planning, including the potential for a formal advance directive or exploring long-term care options.

Discussion Questions:

How can the interdisciplinary team address Mr. Martinez’s increasing agitation and aggression while respecting his dignity and autonomy?

What strategies can be employed to manage Mr. Martinez’s wandering behavior, especially at night?

How can the healthcare team provide support to Mr. Martinez’s son and daughter-in-law to reduce their caregiving burden and prevent burnout?

How would you approach a conversation with the family about long-term care planning and the potential need for assisted living or memory care?

What role does cultural competence play in Mr. Martinez’s care, and how can the team ensure his cultural and linguistic preferences are respected?

Simulation Objectives:

Develop an interdisciplinary care plan that addresses both Mr. Martinez’s cognitive decline and the well-being of his caregivers.

Practice culturally sensitive communication strategies for engaging with Hispanic patients and families.

Discuss ethical decision-making in the context of cognitive decline, including issues of autonomy, safety, and long-term care planning.

Collaborate with family members to ensure patient-centered care that considers both the medical and social dimensions of caregiving.

This case focuses on cognitive decline, caregiver burden, and the complexities of managing behavioral symptoms in Alzheimer's disease. It encourages interdisciplinary collaboration, cultural competence, and ethical decision-making in geriatric care.

# Mrs. Gloria Martinez

Patient Profile:

- Name: Gloria Martinez
- Age: 85
- Gender: Female
- Ethnicity: Hispanic
- Marital Status: Divorced
- Living Situation: Lives in an assisted living facility
- Insurance: Medicare and Medicaid

Medical History:

- Alzheimer's disease (diagnosed 3 years ago)
- Hypertension (diagnosed 20 years ago)
- Chronic Kidney Disease Stage 3 (diagnosed 5 years ago)
- Osteoporosis (diagnosed 10 years ago)
- Recent hip fracture (3 months ago)
- Current Medications:
- Donepezil 10 mg daily
- Amlodipine 5 mg daily
- Alendronate 70 mg weekly
- Calcium 500 mg BID with Vitamin D
- Lisinopril 10 mg daily
- Hydrocodone/acetaminophen 5/325 mg PRN for pain

Chief Complaint: Mrs. Martinez presents with increasing confusion, difficulty in mobility following her hip fracture, and occasional agitation.

Social History:

- Former librarian
- Has two sons, one of whom visits weekly, and the other lives out of state and visits occasionally
- Speaks primarily Spanish, with limited English proficiency
- Enjoys reading and listening to music but has had decreased engagement due to cognitive decline

Physical Examination Findings:

- BP: 145/85 mmHg
- HR: 82 bpm
- RR: 16 breaths/min
- Temp: 97.8°F
- Weight: 135 lbs, Height: 5'2"
- General: Appears anxious, requires assistance to stand
- HEENT: No acute distress
- Cardiovascular: Regular rate and rhythm, no murmurs
- Respiratory: Clear to auscultation bilaterally
- Musculoskeletal: Pain and limited range of motion in the right hip
- Neurological: Disoriented to time and place, follows simple commands

Simulation Objectives:

- Medical Students:
- Develop a management plan for Alzheimer's disease and its complications.
- Address issues related to polypharmacy and medication adherence.
- Evaluate and manage chronic kidney disease in the context of other comorbidities.
- Nursing Students:
- Conduct a comprehensive assessment, focusing on pain management and mobility.
- Develop a care plan to address fall prevention and safe ambulation.
- Educate the patient and family about managing Alzheimer's disease and ensuring medication safety.
- Social Work Students:
- Assess the impact of cognitive decline on Mrs. Martinez's social interactions and quality of life.
- Identify community resources and support systems for patients with Alzheimer's disease.
- Collaborate with the healthcare team to address cultural and language barriers in care provision.

Discussion Points:

- Balancing the management of multiple chronic conditions in elderly patients.
- The importance of fall prevention strategies and rehabilitation post-hip fracture.
- Addressing the psychosocial aspects of living with Alzheimer's disease.
- Ensuring culturally competent care and effective communication with patients and families.
- This case study provides an opportunity for students to engage in interdisciplinary collaboration and develop a comprehensive, patient-centered care plan for elderly patients.

# Mrs. Li Mei

Background:

Patient: Mrs. Li Mei Age: 81 years Gender: Female Ethnicity: Chinese Marital Status: Widowed Living Situation: Lives with her youngest daughter in a multi-generational home.

Medical History:

- Osteoarthritis: Severe in both knees, limits mobility significantly.
- Hypertension: Controlled with medication.
- Cataracts: Post-operative state for the left eye, scheduled for the right eye.
- Gastritis: Chronic, managed with diet and proton pump inhibitors (PPIs).
- Insomnia: Has been using herbal remedies, but recently prescribed zolpidem by her GP due to worsening sleep quality.

Social History:

- Former tailor, still enjoys mending clothes for family members when able.
- Non-smoker, drinks traditional Chinese tea daily.
- No known allergies.

Current Presentation:Mrs. Li was brought to the geriatric clinic by her daughter due to concerns about her increasing forgetfulness, frequent falls at home, and an episode where she was found by neighbors wandering in the neighborhood unsure of how to get back home. She also mentions feeling dizzy on standing, which has worsened since starting zolpidem. 

Assessment:

- Vitals: BP: 130/85 mmHg lying, 110/75 mmHg standing, HR: 70 bpm, RR: 16 breaths/min, Temp: 98.2°F, O2 Sat: 98% on room air.
- General Appearance: Thin, well-dressed, appears slightly confused but cooperative.
- Mental Status: Mini-Mental State Examination (MMSE) score of 20/30, with noticeable deficits in orientation to time and recall.
- Physical Exam:
- Musculoskeletal: Decreased range of motion in knees, varus deformity, uses a cane for support.
- Neurological: Normal except for proprioception loss in lower limbs, likely due to osteoarthritis or neuropathy.
- Vision: Post-operative changes in left eye, cataracts still significant in the right eye.

Medications:

- Amlodipine for hypertension
- Omeprazole for gastritis
- Zolpidem for insomnia

Psychosocial Factors:

Feels she is losing her role in the family due to her physical and cognitive limitations.

Cultural expectation to not burden her children, causing her emotional distress about her condition.

Interdisciplinary Learning Objectives:

- Medical Students:
- Investigate the cause of falls and dizziness, considering postural hypotension, medication side effects, or vestibular issues.
- Manage the interaction between osteoarthritis pain management and her other medications, considering the risk of gastritis.
- Nursing Students:
- Develop a care plan focusing on fall prevention, safe mobility, and home modifications.
- Education on medication management, particularly the risks associated with zolpidem in the elderly.
- Social Work Students:
- Assess the family dynamics and support system, addressing the cultural aspects of caregiving and aging.
- Facilitate discussions on balancing independence with safety, possibly introducing home care services or community resources.
- Interdisciplinary Goals:
- Enhance Mrs. Li's quality of life through improved mobility, cognitive function, and social engagement.
- Coordinate care to ensure all aspects of her health, from physical to mental and social well-being, are addressed holistically.

Simulation Scenario:

- Setting: Home visit followed by a multidisciplinary clinic appointment.
- Tasks:
- Assess the home for fall risks and suggest modifications (e.g., removing rugs, installing handrails).
- Review medication regimen, especially looking at zolpidem's impact on her cognition and balance.
- Plan for cataract surgery on the right eye, considering her overall health and anesthesia risks.
- Address her emotional and social needs through engagement in family activities tailored to her capabilities.

Simulation Outcome:

Students should identify the need for a physical therapy referral for knee osteoarthritis, reassess her sleep medication due to side effects, and involve occupational therapy for daily living activities. They should also explore community programs that align with her interests to enhance social interaction, respecting her cultural background and family dynamics.

# Mrs. Gwendolyn Moss

Patient Information

- Name: Gwendolyn Moss
- Age: 81
- Gender: Female
- Marital Status: Widowed (2 years ago)
- Living Situation: Lives in a continuing care retirement community (CCRC)

Chief Complaint

"I'm having trouble keeping my balance, and I'm worried about falling. Also, my arthritis is acting up something awful."

History of Present Illness

Mrs. Moss reports increasing difficulty with balance over the past month. She's had two near-falls in the past week and is now using a walker, which she resents. She also complains of worsening pain and stiffness in her hands and knees, making it hard to perform daily activities. Mrs. Moss is frustrated by her declining mobility and independence.

Past Medical History

- Rheumatoid Arthritis (diagnosed 25 years ago)
- Osteoporosis
- Macular Degeneration
- Hypertension
- Gastroesophageal Reflux Disease (GERD)
- History of major depressive episode (5 years ago)

Medications

- Methotrexate 15 mg weekly
- Prednisone 5 mg daily
- Alendronate 70 mg weekly
- Calcium + Vitamin D supplement daily
- Lisinopril 20 mg daily
- Omeprazole 20 mg daily
- Acetaminophen 500 mg as needed for pain

Allergies

- Sulfa drugs (severe rash)
- Penicillin (nausea)

Social History

- Retired librarian and community theater actress
- Widowed 2 years ago; husband died of Parkinson's disease
- Two adult children who live in different states
- Moved to CCRC 1 year ago, reluctantly, at children's insistence
- Active in CCRC community theater and book club
- No smoking history, occasional glass of wine with dinner

Review of Systems

- General: Fatigue, no recent weight changes
- Musculoskeletal: Increased joint pain and stiffness, especially in hands and knees
- Neurological: Difficulty with balance, no vertigo
- Ophthalmological: Gradually worsening central vision
- Gastrointestinal: Occasional heartburn
- Psychiatric: Feelings of frustration and some anxiety about loss of independence

Physical Examination

- Vital Signs:
- BP: 138/82 mmHg
- HR: 76 bpm
- RR: 16/min
- Temp: 36.7°C (98.1°F)
- O2 Sat: 97% on room air
- General: Alert, oriented, appears frustrated but engaged
- Musculoskeletal:
- Hands show bilateral ulnar deviation and swan-neck deformities
- Knees with bony enlargement and crepitus
- Decreased range of motion in affected joints
- Neurological:
- Strength 5/5 in all extremities
- Proprioception decreased in lower extremities
- Romberg test positive
- Ophthalmological: Visual acuity 20/70 bilaterally with corrective lenses
- Skin: No rashes, normal turgor

Laboratory and Imaging Results

- Complete Blood Count (CBC):
- WBC: 7,500/µL
- Hemoglobin: 11.8 g/dL (slightly low)
- Platelets: 220,000/µL
- Comprehensive Metabolic Panel: Within normal limits
- Erythrocyte Sedimentation Rate (ESR): 40 mm/hr (elevated)
- C-Reactive Protein (CRP): 2.5 mg/dL (elevated)
- Vitamin D level: 22 ng/mL (insufficient)
- DEXA scan: T-score -2.8 (osteoporosis)

Assessment

- Rheumatoid Arthritis flare
- Increased fall risk due to multiple factors (arthritis, vision impairment, possible neuropathy)
- Osteoporosis
- Macular Degeneration
- Hypertension, well-controlled
- GERD
- Adjustment disorder related to loss of independence
- Possible depression recurrence

Plan

- Rheumatology consultation for management of RA flare
- Physical therapy evaluation for balance training and fall prevention
- Occupational therapy evaluation for adaptive equipment and strategies for ADLs
- Ophthalmology follow-up for macular degeneration management
- Consider addition of Vitamin D supplementation
- Geriatric psychiatry consultation for evaluation of mood and adjustment to life changes
- Social work consultation for:
- Assessment of current living situation and needs
- Exploration of additional support services within CCRC
- Facilitation of family communication and support
- Medication review with focus on fall risk and consideration of deprescribing where appropriate

Simulation Learning Objectives

- Medical Students:
- Manage rheumatoid arthritis flare in an elderly patient
- Develop a comprehensive fall prevention plan
- Recognize and address the interplay between physical and mental health in the elderly
- Navigate the balance between managing chronic conditions and maintaining quality of life
- Nursing Students:
- Conduct a thorough fall risk assessment
- Implement strategies to improve medication adherence in patients with arthritis
- Develop patient education plans for managing multiple chronic conditions
- Recognize signs of adjustment difficulties and depression in elderly patients
- Social Work Students:
- Assess psychosocial impact of declining physical function and independence
- Evaluate appropriateness of current living situation and need for additional services
- Facilitate family discussions about care needs and preferences
- Identify and connect patients with appropriate community resources within a CCRC setting
- Interdisciplinary Objectives:
- Practice effective communication with patients who have sensory impairments
- Collaborate to develop a person-centered care plan that balances safety, independence, and quality of life
- Recognize and address the emotional impact of functional decline in the elderly
- Develop strategies for supporting transitions in care within a CCRC environment

Simulation Scenario Progression

- Initial Assessment: Each discipline conducts their respective assessments, with a focus on fall risk, functional status, and emotional well-being.
- Team Huddle: Interdisciplinary team meets to discuss findings and develop an initial care plan.
- Patient Education: Simulate educating Mrs. Moss about fall prevention strategies and management of RA flare.
- Family Meeting: Conduct a family meeting (including children via video call) to discuss concerns, care needs, and preferences for support.
- Therapeutic Intervention: Role-play a physical therapy session focusing on balance training and a psychiatry consultation addressing adjustment difficulties.
- Care Planning: Develop a comprehensive care plan that addresses medical needs, fall prevention, and psychosocial support.
- CCRC Coordination: Simulate a meeting with CCRC staff to coordinate implementation of new support services and environmental modifications.
- Follow-up: Simulate a follow-up visit to assess the effectiveness of interventions, medication changes, and adjustment to new support services.

This case study provides multiple learning opportunities for students across healthcare disciplines, focusing on the complex care needs of an older adult dealing with chronic progressive conditions, increased fall risk, and the psychosocial challenges of adjusting to functional decline and changes in living situation.

# Mrs. Agnes Müller

Background:

Patient: Mrs. Agnes Müller Age: 76 years Gender: Female Ethnicity: German Marital Status: Divorced Living Situation: Lives alone in an apartment in an urban area, with a neighbor who checks in on her regularly. Has one son living abroad. 

Medical History:

- Rheumatoid Arthritis: Diagnosed 20 years ago, currently managed with methotrexate and low-dose prednisone.
- Chronic Obstructive Pulmonary Disease (COPD): Moderate, requires daily inhalers.
- Type 2 Diabetes: Well-managed with diet, exercise, and metformin.
- Macular Degeneration: Early stages, affecting her reading vision.

Social History:

- Former librarian, very active in local book clubs until recently.
- Quit smoking 10 years ago after a 30-year history of smoking.
- Drinks a glass of wine with dinner occasionally.
- No known allergies.

Current Presentation:Mrs. Müller was brought to the emergency department by her neighbor after a fall at home, which resulted in a hip fracture. She reports increasing difficulty with household chores, feeling weaker, and has mentioned forgetting to take her medications several times in the last month. 

Assessment:

- Vitals: BP: 140/85 mmHg, HR: 80 bpm, RR: 18 breaths/min, Temp: 98.6°F, O2 Sat: 93% on room air, in pain from the fracture.
- General Appearance: Visibly in pain, anxious about her future mobility and independence.
- Mental Status: Montreal Cognitive Assessment (MoCA) score of 22/30, indicating mild cognitive impairment, particularly in areas of memory and complex attention.
- Physical Exam:
- Musculoskeletal: Right hip fracture confirmed by X-ray, limited range of motion, swelling, and tenderness.
- Respiratory: Decreased breath sounds bilaterally, no acute distress at rest.
- Visual: Decreased visual acuity, uses glasses for near vision.

Medications:

- Methotrexate weekly for RA
- Prednisone daily
- Metformin for diabetes
- Albuterol and Tiotropium for COPD
- Vitamin D and Calcium supplements

Psychosocial Factors:

- Expresses fear of losing her independence and becoming dependent on others for care.
- Loneliness due to her son living abroad, though they communicate frequently online.
- Financial concerns about the cost of surgery and rehabilitation.

Interdisciplinary Learning Objectives:

- Medical Students:
- Manage post-operative care for a geriatric patient with multiple comorbidities, focusing on pain management, infection prevention, and early mobilization.
- Consider the interactions between medications for RA, COPD, and diabetes in the context of surgery and recovery.
- Nursing Students:
- Develop a nursing care plan for hip fracture recovery, emphasizing mobility, prevention of pressure ulcers, and patient education on safety at home post...

# Mr. William "Bill" O'Connell

Patient Demographics:

- Age: 85
- Gender: Male
- Marital Status: Widower (wife passed away 1 year ago)
- Living Situation: Lives alone in a assisted living facility
- Children: Two sons, one local and one who lives overseas
- Medical History: Heart Failure, Chronic Obstructive Pulmonary Disease (COPD), Type 2 Diabetes, Moderate hearing loss, Glaucoma

Presenting Complaint:

Mr. O'Connell is referred to the facility's nurse practitioner by the activities director due to concerns about his declining participation in social activities and increasing withdrawal from other residents. He has also been experiencing increased shortness of breath and fatigue, making it difficult for him to engage in activities he once enjoyed.

Physical Examination:

- Vital Signs: BP 140/90 mmHg, HR 90 bpm, RR 22, Temp 98.8 F
- General Appearance: Appears tired and somewhat disheveled, using accessory muscles to breathe
- Respiratory: Diminished breath sounds, wheezes on auscultation
- Cardiovascular: Elevated heart rate, mild edema in lower extremities
- Other: Reports difficulty hearing during conversation

Social History:

- Mr. O'Connell was a firefighter for 30 years and a very active member of his community.
- He enjoyed socializing with friends, playing cards, and attending community events.
- He is struggling to cope with the loss of his wife and his declining health.
- He reports feeling isolated and lonely, even within the assisted living facility.
- He is reluctant to participate in activities due to his shortness of breath and fatigue.

Learning Objectives:

- Chronic Disease Management in Older Adults: Students will need to assess and manage Mr. O'Connell's multiple chronic conditions, including heart failure, COPD, and diabetes.
- Sensory Impairment: Students will need to address his hearing loss and vision impairment and ensure he has access to assistive devices and communication strategies.
- Grief and Loss: Students will need to assess and address his grief and provide support and resources to help him cope with the loss of his wife.
- Social Isolation and Depression: Students will need to assess his social and emotional well-being and develop strategies to promote social engagement and reduce feelings of loneliness.
- Interprofessional Collaboration: Students from different disciplines will need to collaborate to develop a comprehensive care plan that addresses his physical, emotional, and social needs.

Simulation Activities:

- Respiratory Assessment and Management: Students can practice assessing and managing his respiratory symptoms, including oxygen therapy and breathing techniques.
- Communication Strategies for Hearing Loss: Students can practice communication strategies to effectively interact with Mr. O'Connell, considering his hearing impairment.
- Grief Counseling Role-Play: Students can role-play grief counseling sessions to provide support and explore coping mechanisms.
- Social Activity Planning: Students can collaborate to plan social activities that are tailored to Mr. O'Connell's interests and physical limitations.
- Interprofessional Case Conference: Students from different disciplines can discuss Mr. O'Connell's care plan and coordinate services.

Assessment:

- Students can be assessed on their ability to:
- Assess and manage chronic diseases in older adults
- Address sensory impairments and communication challenges
- Provide grief support and counseling
- Promote social engagement and reduce loneliness
- Collaborate effectively with other disciplines

This case study highlights the interconnectedness of physical health, sensory impairments, grief, and social isolation in older adults. It provides students with an opportunity to develop a comprehensive understanding of geriatric care and the importance of interprofessional collaboration in addressing the complex needs of this population.

# Mr. Johnathan "Jack" O'Connor

Background:

Patient: Mr. Johnathan "Jack" O'Connor Age: 87 years Gender: Male Ethnicity: Irish-American Marital Status: Married Living Situation: Lives with his wife in a two-story house; they have an elevator installed due to his mobility issues. 

Medical History:

- Coronary Artery Disease: Had a myocardial infarction 7 years ago, followed by coronary artery bypass grafting (CABG).
- Chronic Atrial Fibrillation: On anticoagulation therapy.
- Diabetes Mellitus Type 2: Poorly controlled, leading to peripheral neuropathy.
- Chronic Venous Insufficiency: With history of leg ulcers.
- Hearing Loss: Uses hearing aids but still has considerable difficulty in noisy environments.

Social History:

- Retired steelworker, active in veterans' affairs, proud of his service in the military.
- Former smoker, quit 25 years ago. Drinks 2-3 beers weekly at social gatherings.
- No known drug allergies.

Current Presentation: Mr. O'Connor was referred to...

Case Study: Mr. Samuel O'Reilly

Patient Profile:

- Name: Samuel O'Reilly
- Age: 79
- Gender: Male
- Ethnicity: Caucasian
- Marital Status: Widowed
- Living Situation: Resides in a skilled nursing facility
- Insurance: Medicare

Medical History:

- Parkinson's disease (diagnosed 10 years ago)
- Hypertension (diagnosed 15 years ago)
- Chronic Kidney Disease Stage 4 (diagnosed 3 years ago)
- Depression (diagnosed 5 years ago)
- History of myocardial infarction (7 years ago)
- Current Medications:
- Carbidopa/Levodopa 25/100 mg TID
- Metoprolol 50 mg BID
- Sertraline 50 mg daily
- Amlodipine 5 mg daily
- Atorvastatin 40 mg daily

Chief Complaint: Mr. O'Reilly presents with increasing tremors, difficulty walking, frequent episodes of hypotension, and feelings of sadness and isolation.

Social History:

- Former construction worker
- Has two daughters who visit regularly
- Enjoys playing the piano but has difficulty due to tremors
- Prefers spending time alone, has limited interaction with other residents

Physical Examination Findings:

- BP: 115/75 mmHg (postural drop noted)
- HR: 65 bpm
- RR: 18 breaths/min
- Temp: 97.9°F
- Weight: 170 lbs, Height: 5'9"
- General: Appears anxious, resting tremor observed
- HEENT: No acute distress
- Cardiovascular: Regular rate and rhythm, no murmurs
- Respiratory: Clear to auscultation bilaterally
- Musculoskeletal: Rigidity in limbs, bradykinesia
- Neurological: Resting tremor, cogwheel rigidity, shuffling gait

Simulation Objectives:

- Medical Students:
- Conduct a comprehensive assessment of Parkinson's disease and its progression.
- Develop a management plan for symptomatic relief and medication adjustments.
- Address issues related to chronic kidney disease and its impact on overall health.
- Nursing Students:
- Perform a detailed physical examination, focusing on mobility and safety.
- Develop a care plan to manage tremors, gait disturbances, and hypotension.
- Educate the patient on fall prevention, medication adherence, and lifestyle modifications.
- Social Work Students:
- Assess the impact of Mr. O'Reilly's medical conditions on his mental health and social well-being.
- Identify community resources and support groups for patients with Parkinson's disease.
- Collaborate with the healthcare team to address Mr. O'Reilly's psychosocial needs and improve his quality of life.

Discussion Points:

- Managing the progression of neurodegenerative diseases in elderly patients.
- Importance of addressing both physical and mental health in geriatric care.
- Strategies to enhance social engagement and reduce feelings of isolation.
- Role of interdisciplinary teamwork in providing holistic care.
- This case study encourages students to work together across disciplines to deliver comprehensive care for elderly patients, addressing both their medical and psychosocial needs.

# Mr. Samuel O'Reilly

Patient Profile:

Name: Samuel O'Reilly

Age: 79

Gender: Male

Ethnicity: Caucasian

Marital Status: Widowed

Living Situation: Resides in a skilled nursing facility

Insurance: Medicare

Medical History:

Parkinson's disease (diagnosed 10 years ago)

Hypertension (diagnosed 15 years ago)

Chronic Kidney Disease Stage 4 (diagnosed 3 years ago)

Depression (diagnosed 5 years ago)

History of myocardial infarction (7 years ago)

Current Medications:

Carbidopa/Levodopa 25/100 mg TID

Metoprolol 50 mg BID

Sertraline 50 mg daily

Amlodipine 5 mg daily

Atorvastatin 40 mg daily

Chief Complaint: Mr. O'Reilly presents with increasing tremors, difficulty walking, frequent episodes of hypotension, and feelings of sadness and isolation.

Social History:

Former construction worker

Has two daughters who visit regularly

Enjoys playing the piano but has difficulty due to tremors

Prefers spending time alone, has limited interaction with other residents

Physical Examination Findings:

BP: 115/75 mmHg (postural drop noted)

HR: 65 bpm

RR: 18 breaths/min

Temp: 97.9°F

Weight: 170 lbs, Height: 5'9"

General: Appears anxious, resting tremor observed

HEENT: No acute distress

Cardiovascular: Regular rate and rhythm, no murmurs

Respiratory: Clear to auscultation bilaterally

Musculoskeletal: Rigidity in limbs, bradykinesia

Neurological: Resting tremor, cogwheel rigidity, shuffling gait

Simulation Objectives:

Medical Students:

Conduct a comprehensive assessment of Parkinson's disease and its progression.

Develop a management plan for symptomatic relief and medication adjustments.

Address issues related to chronic kidney disease and its impact on overall health.

Nursing Students:

Perform a detailed physical examination, focusing on mobility and safety.

Develop a care plan to manage tremors, gait disturbances, and hypotension.

Educate the patient on fall prevention, medication adherence, and lifestyle modifications.

Social Work Students:

Assess the impact of Mr. O'Reilly's medical conditions on his mental health and social well-being.

Identify community resources and support groups for patients with Parkinson's disease.

Collaborate with the healthcare team to address Mr. O'Reilly's psychosocial needs and improve his quality of life.

Discussion Points:

Managing the progression of neurodegenerative diseases in elderly patients.

Importance of addressing both physical and mental health in geriatric care.

Strategies to enhance social engagement and reduce feelings of isolation.

Role of interdisciplinary teamwork in providing holistic care.

This case study encourages students to work together across disciplines to deliver comprehensive care for elderly patients, addressing both their medical and psychosocial needs.

# Mrs. Doris Patel

Patient Profile:

Name: Doris Patel

Age: 77 years old

Gender: Female

Ethnicity: Indian American

Marital Status: Married for 52 years

Living Situation: Lives with her husband in a senior apartment community

Occupation: Retired nurse

Insurance: Medicare Part D and private supplemental insurance

Primary Language: English (fluent), Hindi (native language)

Advance Directive: Yes (wishes to remain at home with no extraordinary life-saving measures)

Chief Complaint:

Mrs. Patel presents to her primary care physician with complaints of worsening chronic lower back and joint pain that has significantly affected her mobility and daily functioning over the past six months. She also mentions frequent episodes of dizziness, which have resulted in a near fall. She feels her medications may be contributing to her symptoms, and her family is concerned about her taking too many medications.

Past Medical History:

Osteoarthritis (diagnosed 10 years ago)

Hypertension (diagnosed 20 years ago)

Chronic Kidney Disease (Stage 2)

Depression (diagnosed 5 years ago)

Gastroesophageal Reflux Disease (GERD)

History of falls (one fall last year, resulting in a wrist fracture)

Insomnia

Current Medications:

Lisinopril 10 mg once daily (for hypertension)

Amlodipine 5 mg once daily (for hypertension)

Acetaminophen 500 mg every 6 hours as needed (for pain)

Ibuprofen 400 mg every 8 hours as needed (for arthritis)

Omeprazole 20 mg once daily (for GERD)

Sertraline 50 mg once daily (for depression)

Melatonin 5 mg at bedtime (for sleep)

Multivitamin daily

Social History:

Mrs. Patel is originally from India and moved to the U.S. 30 years ago. She is culturally connected to her heritage and prefers to cook traditional Indian food.

She lives with her husband in a senior apartment community where they are both active in social activities.

They have two adult children, both living out of state. They stay in contact regularly by phone but visit only a few times a year.

Mrs. Patel used to enjoy gardening and walking, but her chronic pain has limited her physical activities in recent years.

She abstains from alcohol and smoking.

Functional Status:

Mrs. Patel is independent with most ADLs, but her chronic pain has significantly impacted her ability to perform IADLs, particularly housekeeping and grocery shopping.

She experiences difficulty walking long distances, climbing stairs, and standing for extended periods due to joint pain.

She uses a cane for balance and occasionally needs her husband’s assistance with household chores.

She has been experiencing dizziness more frequently, and she attributes this to her medications but is unsure which one may be causing it.

Review of Systems:

General: Fatigue, chronic pain, dizziness

Cardiovascular: No chest pain or palpitations, controlled hypertension

Respiratory: No shortness of breath or cough

Gastrointestinal: No nausea or vomiting, occasional indigestion (controlled with omeprazole)

Genitourinary: No urinary complaints, but occasional urgency

Musculoskeletal: Chronic lower back pain, worsening joint pain (particularly knees and hands)

Neurological: Dizziness, no recent falls, no confusion

Psychological: Feels sad occasionally, but mood is stable on current antidepressant

Sleep: Difficulty falling asleep, wakes up frequently due to pain

Physical Examination Findings:

General: Alert and oriented, appears well-nourished

Vital signs:

Blood pressure: 135/80 mmHg

Pulse: 68 bpm, regular

Respiratory rate: 16 breaths/min

Oxygen saturation: 98% on room air

Temperature: 98.2°F

Cardiovascular: Regular heart sounds, no murmurs

Lungs: Clear to auscultation bilaterally

Abdomen: Soft, non-tender

Musculoskeletal: Tenderness over lower lumbar spine, limited range of motion in knees due to osteoarthritis, mild swelling in finger joints

Neurological: Intact cranial nerves, no motor or sensory deficits, reports dizziness with quick changes in position

Lab Results:

Complete Blood Count (CBC): Normal

Basic Metabolic Panel (BMP): Slightly elevated creatinine (1.3 mg/dL)

Liver Function Tests (LFTs): Normal

Erythrocyte Sedimentation Rate (ESR): Elevated at 40 mm/h (suggestive of inflammation)

Urinalysis: Normal

Interdisciplinary Learning Objectives:

Medical Students:

Perform a comprehensive assessment of Mrs. Patel’s chronic pain and evaluate the impact of polypharmacy on her symptoms.

Review and adjust her medication regimen, considering her chronic kidney disease, hypertension, and pain management needs.

Develop a plan to manage her osteoarthritis and dizziness, including potential non-pharmacologic interventions.

Assess her risk of future falls and recommend appropriate preventive measures.

Nursing Students:

Conduct a functional assessment to evaluate Mrs. Patel’s mobility and fall risk, and provide education on fall prevention strategies.

Educate Mrs. Patel and her family on pain management options, including non-drug therapies like physical therapy and lifestyle modifications.

Monitor Mrs. Patel’s response to pain medications and assess for potential side effects, including those related to polypharmacy.

Implement strategies to manage Mrs. Patel’s sleep disturbances and educate her on healthy sleep hygiene practices.

Social Work Students:

Explore the emotional and social impacts of chronic pain on Mrs. Patel’s quality of life, including her relationships and sense of independence.

Assess for any signs of caregiver burden in Mrs. Patel’s husband, and provide resources for additional support or respite care.

Discuss the cultural implications of pain management, including Mrs. Patel’s preferences for non-Western treatments or dietary considerations.

Assist the family in exploring community resources, such as senior exercise programs or pain management support groups.

Discussion Questions:

How can the healthcare team collaborate to develop an effective pain management plan for Mrs. Patel while minimizing the risks associated with polypharmacy?

What strategies can be employed to address Mrs. Patel’s dizziness and reduce her risk of falls?

How can the interdisciplinary team involve Mrs. Patel’s husband in her care, particularly in supporting her functional independence and preventing caregiver burnout?

What are some culturally sensitive approaches the team could use to incorporate Mrs. Patel’s preferences and beliefs into her treatment plan?

How would you address Mrs. Patel’s insomnia in a way that avoids contributing to polypharmacy or worsening her current conditions?

Simulation Objectives:

Develop an interdisciplinary care plan that balances the management of chronic pain and polypharmacy in an elderly patient.

Practice communication strategies to educate elderly patients and their families on safe medication use and fall prevention.

Collaborate with healthcare team members to address the psychosocial aspects of living with chronic pain, including its effects on mental health and social relationships.

Ensure culturally competent care by considering Mrs. Patel’s preferences for pain management and the role her cultural background plays in decision-making.

This case study focuses on the challenges of managing polypharmacy, chronic pain, and functional decline in elderly patients. It highlights the importance of interdisciplinary teamwork to address the medical, functional, and psychosocial needs of patients like Mrs. Patel, with attention to cultural sensitivity.

# Mrs. Lila Patel

Patient Information

- Name: Lila Patel
- Age: 72
- Gender: Female
- Marital Status: Married (48 years)
- Living Situation: Lives with husband in a single-story home

Chief Complaint

"I've been having terrible pain in my stomach, and I'm so tired all the time. I think something is seriously wrong."

History of Present Illness

Mrs. Patel reports a 3-month history of worsening abdominal pain, primarily in the upper right quadrant. The pain is accompanied by nausea, loss of appetite, and an unintentional 15-pound weight loss. She also notes increasing fatigue and occasional night sweats. Mrs. Patel admits she has delayed seeking medical attention due to fear of a serious diagnosis and concerns about burdening her family.

Past Medical History

- Hepatitis B (diagnosed in young adulthood)
- Type 2 Diabetes Mellitus (diagnosed 10 years ago)
- Hypertension
- Hyperlipidemia
- Osteoarthritis of the knees

Medications

- Metformin 1000 mg twice daily
- Lisinopril 20 mg daily
- Atorvastatin 40 mg daily
- Acetaminophen 500 mg as needed for pain
- Glucosamine supplement daily

Allergies

- Sulfa drugs (rash)

Social History

- Retired elementary school teacher
- Lives with husband who has mild hearing impairment
- Two adult children living in nearby cities
- Active in local Hindu temple community
- No history of alcohol use or smoking
- Immigrated from India 40 years ago

Review of Systems

- General: Fatigue, unintentional weight loss, night sweats
- Gastrointestinal: Right upper quadrant abdominal pain, nausea, decreased appetite
- Musculoskeletal: Chronic knee pain
- Neurological: No headaches or dizziness
- Cardiovascular: No chest pain or palpitations
- Respiratory: No shortness of breath or cough

Physical Examination

- Vital Signs:
- BP: 142/88 mmHg
- HR: 88 bpm
- RR: 18/min
- Temp: 37.2°C (99°F)
- O2 Sat: 98% on room air
- General: Alert, oriented, appears uncomfortable and fatigued
- Abdominal: Tender to palpation in right upper quadrant, palpable mass noted
- Cardiovascular: Regular rate and rhythm, no murmurs
- Respiratory: Clear to auscultation bilaterally
- Musculoskeletal: Crepitus in both knees, full range of motion
- Skin: No jaundice noted

Laboratory and Imaging Results

- Complete Blood Count (CBC):
- WBC: 11,500/µL (elevated)
- Hemoglobin: 10.8 g/dL (low)
- Platelets: 380,000/µL
- Comprehensive Metabolic Panel:
- AST: 150 U/L (elevated)
- ALT: 165 U/L (elevated)
- Alkaline Phosphatase: 450 U/L (elevated)
- Total Bilirubin: 1.8 mg/dL (slightly elevated)
- Albumin: 3.2 g/dL (low)
- Alpha-fetoprotein (AFP): 380 ng/mL (significantly elevated)
- HbA1c: 7.6%
- Hepatitis B surface antigen: Positive
- Abdominal CT scan: 6 cm mass in the right lobe of the liver, consistent with hepatocellular carcinoma

Assessment

- Probable hepatocellular carcinoma
- Chronic Hepatitis B infection
- Type 2 Diabetes Mellitus, suboptimal control
- Hypertension
- Anemia of chronic disease
- Osteoarthritis of the knees
- Caregiver stress (for husband with hearing impairment)

Plan

- Urgent oncology referral for further evaluation and treatment planning of liver mass
- Hepatology consultation for management of chronic Hepatitis B
- Pain management consultation
- Nutrition consultation for cancer-related weight loss and diabetes management
- Social work consultation for:
- Psychosocial support for new cancer diagnosis
- Cultural considerations in care planning
- Caregiver support assessment
- Palliative care consultation for symptom management and goals of care discussion
- Adjust diabetes management considering liver function and potential cancer treatment
- Consider screening for hepatitis in close contacts

Simulation Learning Objectives

- Medical Students:
- Recognize signs and symptoms of hepatocellular carcinoma in a patient with chronic Hepatitis B
- Develop a comprehensive care plan for a patient with newly diagnosed cancer and multiple comorbidities
- Understand the importance of hepatitis screening and prevention in at-risk populations
- Navigate the complexities of breaking bad news to patients from diverse cultural backgrounds
- Nursing Students:
- Conduct a culturally sensitive nursing assessment for a patient with a new cancer diagnosis
- Implement appropriate pain management strategies for cancer-related pain
- Develop patient education plans for managing multiple chronic conditions alongside cancer
- Recognize and address psychosocial needs of patients facing a life-threatening diagnosis
- Social Work Students:
- Provide culturally appropriate psychosocial support for a patient and family dealing with a new cancer diagnosis
- Assess and address caregiver needs in the context of a serious illness
- Navigate ethical considerations related to truth-telling and family involvement in different cultural contexts
- Identify and connect patients with appropriate community resources for cancer support
- Interdisciplinary Objectives:
- Practice effective, culturally sensitive communication when delivering serious news
- Collaborate to develop a person-centered care plan that addresses medical, functional, and psychosocial needs
- Recognize and address cultural factors that may impact treatment decisions and care planning
- Develop strategies for supporting both the patient and their caregiver in the context of a serious illness

Simulation Scenario Progression

- Initial Assessment: Each discipline conducts their respective assessments, with a focus on cultural sensitivity and breaking bad news.
- Team Huddle: Interdisciplinary team meets to discuss findings and develop an initial care plan.
- Breaking Bad News: Simulate the process of informing Mrs. Patel and her family about the cancer diagnosis, with attention to cultural considerations.
- Family Meeting: Conduct a family meeting to discuss treatment options, prognosis, and care preferences.
- Care Planning: Develop a comprehensive care plan that addresses cancer management, symptom control, and management of comorbidities.
- Cultural Competence: Address cultural and spiritual needs in the care plan, including involvement of community supports.
- Ethical Discussion: Navigate ethical considerations related to truth-telling, family involvement in decision-making, and end-of-life care planning.
- Follow-up: Simulate a follow-up visit to assess coping, symptom management, and adjust the care plan as needed.

This case study provides multiple learning opportunities for students across healthcare disciplines, focusing on the complex care needs of an older adult with a new cancer diagnosis, multiple chronic conditions, and important cultural considerations impacting care.

# Mrs. Anna Patel

Patient Profile:

- Name: Anna Patel
- Age: 80
- Gender: Female
- Ethnicity: Indian American
- Marital Status: Widowed
- Living Situation: Lives alone in an apartment
- Insurance: Medicare

Medical History:

- Coronary Artery Disease (diagnosed 10 years ago)
- Type 2 Diabetes Mellitus (diagnosed 20 years ago)
- Rheumatoid Arthritis (diagnosed 15 years ago)
- Moderate hearing loss (uses hearing aids)
- Depression (diagnosed 3 years ago)
- Current Medications:
- Metformin 500 mg BID
- Atorvastatin 20 mg daily
- Methotrexate 7.5 mg weekly
- Insulin as needed
- Sertraline 25 mg daily

Chief Complaint: Mrs. Patel presents with worsening joint pain, difficulty managing her blood sugar levels, and increased feelings of loneliness and sadness.

Social History:

- Former seamstress
- Has three children, one of whom lives nearby and visits weekly
- Enjoys traditional Indian music and cooking but has reduced participation due to arthritis
- Active in a local senior center but has decreased involvement recently

Physical Examination Findings:

- BP: 140/85 mmHg
- HR: 78 bpm
- RR: 18 breaths/min
- Temp: 98.1°F
- Weight: 160 lbs, Height: 5'3"
- General: Appears fatigued, frail
- HEENT: Uses bilateral hearing aids
- Cardiovascular: Regular rate and rhythm, no murmurs
- Respiratory: Clear to auscultation bilaterally
- Musculoskeletal: Swollen and tender joints, decreased range of motion
- Neurological: Alert and oriented, mild depressive symptoms

Simulation Objectives:

- Medical Students:
- Develop a comprehensive management plan for rheumatoid arthritis and diabetes.
- Address polypharmacy and potential drug interactions.
- Evaluate and manage coronary artery disease in the context of her other conditions.
- Nursing Students:
- Perform a thorough physical assessment, focusing on joint pain and mobility.
- Develop a care plan for managing chronic pain and blood sugar levels.
- Educate the patient on lifestyle modifications and self-care strategies.
- Social Work Students:
- Assess the impact of Mrs. Patel's health conditions on her daily life and social interactions.
- Identify community resources and support systems for elderly patients with chronic illnesses.
- Collaborate with the healthcare team to address Mrs. Patel's psychosocial needs and improve her quality of life.

Discussion Points:

- Balancing the management of multiple chronic conditions in elderly patients.
- Strategies for addressing pain management and improving mobility.
- The importance of social support and community engagement in maintaining mental health.
- The role of interdisciplinary teamwork in providing holistic care.
- This case study allows students to engage in collaborative, patient-centered care, focusing on the unique challenges and needs of elderly patients with multiple chronic conditions.

# Mr. Abdul Rahman

Background:Patient: Mr. Abdul Rahman Age: 79 years Gender: Male Ethnicity: South Asian (Pakistani)Marital Status: MarriedLiving Situation: Resides with his extended family in a large house, which includes his children and grandchildren. 

Medical History:

- Type 2 Diabetes: Poorly controlled with episodes of hypoglycemia, currently on insulin therapy.
- Chronic Kidney Disease (CKD): Stage 3, due to diabetic nephropathy.
- Benign Prostatic Hyperplasia (BPH): Symptomatic, managed with medication.
- Anxiety: Occasional episodes, managed with counseling rather than medication due to concerns over polypharmacy.

Social History:

- Retired civil engineer, enjoyed gardening but has reduced activity due to mobility issues.
- Non-smoker, occasionally enjoys traditional sweets which conflicts with his diabetic diet.
- No known drug allergies.

Current Presentation: Mr. Rahman has been brought to the clinic by his son due to increased forgetfulness,...

# Mr. Michael Roberts

Patient Profile:

- Name: Michael Roberts
- Age: 81
- Gender: Male
- Ethnicity: African American
- Marital Status: Married
- Living Situation: Lives with his wife in a single-story home
- Insurance: Medicare

Medical History:

- Chronic Obstructive Pulmonary Disease (COPD) (diagnosed 9 years ago)
- Type 2 Diabetes Mellitus (diagnosed 18 years ago)
- Hypertension (diagnosed 20 years ago)
- Benign Prostatic Hyperplasia (BPH) (diagnosed 10 years ago)
- Depression (diagnosed 2 years ago)
- Current Medications:
- Metformin 500 mg BID
- Lisinopril 20 mg daily
- Tamsulosin 0.4 mg daily
- Sertraline 50 mg daily
- Albuterol inhaler PRN

Chief Complaint: Mr. Roberts presents with increasing shortness of breath, difficulty managing his blood sugar levels, and feelings of sadness and isolation.

Social History:

- Retired schoolteacher
- Has three children, all of whom live out of state and visit occasionally
- Enjoys playing chess and reading history books but has reduced activity due to health issues
- Previously active in his church community but has decreased participation recently

Physical Examination Findings:

- BP: 145/85 mmHg
- HR: 85 bpm
- RR: 20 breaths/min
- Temp: 98.4°F
- Weight: 175 lbs, Height: 5'8"
- General: Appears fatigued, using supplemental oxygen
- HEENT: No acute distress
- Cardiovascular: Regular rate and rhythm, mild peripheral edema
- Respiratory: Bilateral wheezing, diminished breath sounds at bases
- Musculoskeletal: Mild tremor in hands
- Neurological: Alert and oriented, mild depressive symptoms

Simulation Objectives:

- Medical Students:
- Conduct a thorough evaluation of COPD and diabetes management.
- Develop an appropriate medication regimen and titration plan.
- Recognize and address symptoms of depression and social isolation.
- Nursing Students:
- Perform a comprehensive physical assessment focusing on respiratory and cardiovascular function.
- Develop a care plan addressing shortness of breath, medication management, and blood sugar control.
- Educate the patient on lifestyle modifications, medication adherence, and use of supplemental oxygen.
- Social Work Students:
- Assess the impact of Mr. Roberts's health conditions on his daily life and family dynamics.
- Identify community resources and support systems for elderly patients with chronic illnesses.
- Collaborate with the healthcare team to address Mr. Roberts's psychosocial needs and improve his quality of life.

Discussion Points:

- Balancing the management of multiple chronic conditions in elderly patients.
- Importance of addressing both physical and mental health in geriatric care.
- Strategies for improving quality of life and reducing hospital readmissions.
- Role of interdisciplinary teamwork in providing comprehensive care.
- This case study provides an opportunity for students from different health disciplines to engage in collaborative, patient-centered care, focusing on the unique challenges and needs of elderly patients with multiple chronic conditions.

# Mrs. Amelia Rodriguez

Patient Information

- Name: Amelia Rodriguez
- Age: 75
- Gender: Female
- Marital Status: Divorced
- Living Situation: Lives alone in a senior apartment complex

Chief Complaint

"I'm having trouble breathing and my ankles are swollen. I'm also worried about my memory."

History of Present Illness

Mrs. Rodriguez reports gradually worsening shortness of breath over the past month, especially when lying flat. She has noticed increasing swelling in her ankles and has gained 4 kg in the last two weeks. She also mentions concerns about her memory, stating she sometimes forgets to take her medications and has missed a few doctor's appointments recently.

Past Medical History

- Congestive Heart Failure (diagnosed 3 years ago)
- Type 2 Diabetes Mellitus (diagnosed 15 years ago)
- Chronic Kidney Disease Stage 3
- Hypothyroidism
- Osteoporosis

Medications

- Metformin 1000 mg twice daily
- Furosemide 40 mg daily
- Lisinopril 10 mg daily
- Carvedilol 12.5 mg twice daily
- Levothyroxine 75 mcg daily
- Alendronate 70 mg weekly
- Aspirin 81 mg daily

Allergies

- Sulfa drugs (rash)

Social History

- Retired elementary school teacher
- Divorced 20 years ago, lives alone
- One adult daughter who lives in another state
- Limited social interactions, primarily phone calls with friends
- Non-smoker, occasional glass of wine with dinner
- Fixed income, sometimes struggles with medication costs

Review of Systems

- Cardiovascular: Shortness of breath, orthopnea, ankle edema
- Respiratory: Dyspnea on exertion, occasional dry cough
- Neurological: Forgetfulness, difficulty concentrating
- Musculoskeletal: Chronic lower back pain
- Endocrine: Polyuria, polydipsia
- Psychiatric: Feels lonely, some symptoms of depression

Physical Examination

- Vital Signs:
- BP: 150/90 mmHg
- HR: 88 bpm, irregular
- RR: 22/min
- Temp: 36.7°C (98.0°F)
- O2 Sat: 92% on room air
- General: Alert, oriented to person and place, but unsure of exact date
- Cardiovascular: Irregular rhythm, S3 gallop present, JVD noted
- Respiratory: Bibasilar crackles, decreased breath sounds at bases
- Extremities: 2+ pitting edema in both ankles extending to mid-calf
- Neurological: MMSE (Mini-Mental State Examination) score: 23/30
- Skin: Dry, slight tenting noted

Laboratory and Imaging Results

- Complete Blood Count (CBC):
- Hemoglobin: 10.8 g/dL (low)
- WBC: Within normal limits
- Basic Metabolic Panel (BMP):
- Sodium: 134 mEq/L
- Potassium: 4.8 mEq/L
- BUN: 32 mg/dL (elevated)
- Creatinine: 1.8 mg/dL (elevated)
- HbA1c: 7.8%
- TSH: 5.5 mIU/L (slightly elevated)
- BNP: 850 pg/mL (elevated)
- Chest X-ray: Cardiomegaly, pulmonary edema
- ECG: Atrial fibrillation with rapid ventricular response

Assessment

- Acute on chronic congestive heart failure exacerbation
- Newly diagnosed atrial fibrillation
- Type 2 Diabetes Mellitus, suboptimal control
- Chronic Kidney Disease, worsening
- Mild Cognitive Impairment
- Hypothyroidism, undertreated
- Depression
- Malnutrition risk
- Polypharmacy

Plan

- Admit to hospital for management of heart failure exacerbation
- Cardiology consultation for management of newly diagnosed atrial fibrillation
- Adjust heart failure medications (increase diuretics, consider adding spironolactone)
- Anticoagulation for stroke prevention in atrial fibrillation
- Endocrinology consultation for diabetes management
- Adjust hypothyroidism treatment
- Nephrology consultation for worsening kidney function
- Geriatric psychiatry consultation for cognitive assessment and depression evaluation
- Nutrition consultation for malnutrition risk and diabetes management
- Physical therapy and occupational therapy evaluations
- Social work consultation for home safety evaluation and community resource connection
- Medication reconciliation and consider deprescribing where appropriate

Simulation Learning Objectives

- Medical Students:
- Manage acute heart failure exacerbation in the context of multiple comorbidities
- Diagnose and initiate management for new-onset atrial fibrillation in an elderly patient
- Recognize and address the interplay between heart failure, diabetes, and kidney disease
- Develop a comprehensive care plan considering multiple chronic conditions and cognitive impairment
- Nursing Students:
- Conduct a thorough geriatric nursing assessment, including cognitive and functional assessments
- Implement heart failure management strategies (fluid restrictions, daily weights, medication administration)
- Develop patient education plans for multiple chronic conditions
- Implement fall prevention strategies in the hospital setting
- Social Work Students:
- Assess patient's social support system and ability to manage independently at home
- Evaluate financial constraints affecting medication adherence and access to care
- Identify and connect patient with community resources for seniors
- Discuss advance care planning and assess need for home health services
- Interdisciplinary Objectives:
- Practice effective communication in complex care situations
- Collaborate to develop a person-centered care plan addressing medical, functional, and psychosocial needs
- Recognize and address potential medication-related problems in older adults with polypharmacy
- Develop strategies for supporting medication adherence and chronic disease self-management in the context of mild cognitive impairment

Simulation Scenario Progression

- Initial Assessment: Each discipline conducts their respective assessments in the emergency department or upon admission.
- Team Huddle: Interdisciplinary team meets to discuss findings and develop an initial care plan.
- Acute Management: Simulate management of heart failure exacerbation and new-onset atrial fibrillation.
- Family Conference: Simulate a meeting with Mrs. Rodriguez and her daughter (via video call) to discuss diagnosis, treatment plan, and future care needs.
- Medication Review: Team collaborates on medication reconciliation and develops a plan to simplify the medication regimen.
- Discharge Planning: Team develops a comprehensive plan for transition of care, including medication management, follow-up appointments, and home safety modifications.
- Follow-up: Simulate a post-discharge follow-up appointment to assess adherence to the care plan and make necessary adjustments.

This case study provides multiple learning opportunities for students across healthcare disciplines, focusing on the complex care needs of an older adult with multiple chronic conditions, new acute issues, cognitive concerns, and limited social support.

# Mrs. Anna Rodriguez

Patient Profile:

Name: Anna Rodriguez

Age: 86 years old

Gender: Female

Ethnicity: Hispanic

Marital Status: Married for 60 years

Living Situation: Lives with her husband and adult daughter in their family home

Occupation: Retired homemaker

Insurance: Medicare and Medicaid

Primary Language: Spanish (fluent), limited English

Advance Directive: None; family expresses conflicting views on her care

Chief Complaint:

Mrs. Rodriguez’s daughter brings her to the clinic due to increasing agitation, frequent wandering, and difficulty sleeping at night. Mrs. Rodriguez has advanced dementia (Alzheimer’s disease) and requires 24-hour care. Her daughter, who is the primary caregiver, expresses feelings of being overwhelmed and is unsure how to manage her mother’s worsening symptoms. There is tension in the family regarding placing Mrs. Rodriguez in a nursing home, as her husband wants to continue caring for her at home, but the daughter feels that professional help is needed.

Past Medical History:

Alzheimer’s disease (diagnosed 6 years ago, now advanced stage)

Hypertension (diagnosed 20 years ago)

Osteoporosis (diagnosed 10 years ago)

Urinary incontinence (last 2 years, managed with adult diapers)

Hyperlipidemia (diagnosed 15 years ago)

Current Medications:

Donepezil 10 mg once daily (for dementia)

Lisinopril 10 mg once daily (for hypertension)

Calcium + Vitamin D supplements twice daily (for osteoporosis)

Atorvastatin 20 mg once daily (for hyperlipidemia)

Quetiapine 12.5 mg at bedtime (recently prescribed for agitation)

Social History:

Mrs. Rodriguez has been married to her husband for 60 years. He is 90 years old and in relatively good health but is becoming increasingly frail and less able to assist with her care.

Her adult daughter moved back home two years ago to help care for her. The daughter works part-time from home but feels that caregiving is becoming too much to manage.

Mrs. Rodriguez used to be active in her church and community but has been homebound for the last few years due to her dementia.

She speaks primarily Spanish and occasionally reverts to only Spanish during episodes of confusion, making communication more difficult for her daughter, who is more fluent in English.

Functional Status:

Mrs. Rodriguez is dependent on her family for all ADLs, including feeding, bathing, dressing, and toileting.

She frequently gets up at night and wanders around the house, causing safety concerns.

She is incontinent and wears adult diapers, but her family reports frequent accidents due to her removing the diapers.

She experiences agitation and confusion, especially in the evenings, and has started refusing food and medication at times.

She has fallen twice in the last six months, though no serious injuries were reported.

Review of Systems:

General: Weight loss of 8 pounds over the past 3 months, poor appetite

Cardiovascular: No chest pain or palpitations, controlled hypertension

Respiratory: No cough, no shortness of breath

Gastrointestinal: Poor appetite, no vomiting, occasional constipation

Genitourinary: Urinary incontinence, no recent UTIs

Musculoskeletal: Muscle weakness, limited mobility, osteoporosis-related back pain

Neurological: Advanced dementia, agitation, disorientation, recent increase in wandering and confusion

Psychological: Agitation, mood swings, frequent restlessness, especially at night

Physical Examination Findings:

General: Frail, thin, appears older than stated age

Vital signs:

Blood pressure: 140/85 mmHg

Pulse: 72 bpm, regular

Respiratory rate: 18 breaths/min

Oxygen saturation: 97% on room air

Temperature: 98.0°F

Cardiovascular: Regular heart sounds, no murmurs

Lungs: Clear to auscultation bilaterally

Abdomen: Soft, non-tender, no organomegaly

Musculoskeletal: Kyphosis, tenderness over lumbar spine, reduced range of motion in arms and legs

Neurological: Severe cognitive impairment, disoriented to time, place, and person, unable to follow simple commands

Skin: Dry, evidence of skin breakdown in the sacral area, no open sores

Lab Results:

Complete Blood Count (CBC): Mild anemia (Hemoglobin 11.5 g/dL)

Basic Metabolic Panel (BMP): Creatinine 1.2 mg/dL, glucose 115 mg/dL, electrolytes normal

Vitamin D level: Low (15 ng/mL)

Lipid Panel: LDL 120 mg/dL (above target)

Interdisciplinary Learning Objectives:

Medical Students:

Assess Mrs. Rodriguez’s current cognitive status and identify strategies to manage her agitation, wandering, and sleep disturbances in the context of advanced dementia.

Review her medication regimen and evaluate the risks and benefits of continuing certain medications, especially considering polypharmacy in a patient with advanced dementia.

Develop a plan for managing her urinary incontinence and fall risk, and monitor her skin for signs of breakdown.

Discuss the ethical issues involved in placing a patient with advanced dementia in a long-term care facility versus keeping her at home.

Nursing Students:

Perform a comprehensive functional assessment to determine Mrs. Rodriguez’s risk for falls, skin breakdown, and nutritional deficiencies.

Develop a plan to manage her incontinence and prevent further skin breakdown, including recommendations for improved hygiene and wound care.

Educate the family on non-pharmacological approaches to managing agitation and wandering, such as environmental modifications and establishing routines.

Assess the caregiver burden on Mrs. Rodriguez’s daughter and husband and provide support for stress reduction and self-care.

Social Work Students:

Conduct a psychosocial assessment of the family dynamics, exploring the caregiver burden and the conflicting opinions about placing Mrs. Rodriguez in a nursing home.

Explore community resources for caregiver support, such as respite care, adult day care programs, and home health services.

Facilitate discussions about advance care planning, including the need for a formal advance directive and decisions about long-term care.

Address cultural considerations in the family’s decision-making process, including their preference to care for Mrs. Rodriguez at home and their emotional connection to family caregiving.

Discussion Questions:

How can the healthcare team best manage Mrs. Rodriguez’s dementia-related symptoms, including agitation, wandering, and sleep disturbances, while minimizing the use of medications?

What are the ethical considerations involved in deciding whether to place Mrs. Rodriguez in a long-term care facility, particularly given the conflicting opinions within her family?

How can the interdisciplinary team support Mrs. Rodriguez’s daughter and husband, who are experiencing significant caregiver burden, and what resources could be provided to them?

How should the team address Mrs. Rodriguez’s nutrition and weight loss, considering her advanced dementia and refusal to eat at times?

What role do cultural values play in the family’s decision-making about caregiving, and how can the healthcare team respect these values while providing appropriate recommendations?

Simulation Objectives:

Develop an interdisciplinary care plan that addresses Mrs. Rodriguez’s dementia symptoms, focusing on reducing caregiver burden and managing agitation and wandering in a safe, compassionate manner.

Practice navigating ethical dilemmas related to long-term care placement, especially when family members have conflicting views on the patient’s care.

Explore culturally competent care by integrating the family’s cultural values and preferences into the care plan.

Facilitate conversations about advance care planning and end-of-life decisions with both the patient’s family and the interdisciplinary team.

This case study emphasizes the complexities of managing advanced dementia in an elderly patient while addressing caregiver burden, ethical issues, and the cultural values of the family. It encourages an interdisciplinary approach that incorporates medical, nursing, and social work perspectives to provide comprehensive and compassionate care.

# Mrs. Elena Rodriguez

Background:

Patient: Mrs. Elena Rodriguez Age: 85 years Gender: Female Ethnicity: Hispanic Marital Status: Widowed Living Situation: Resides in an assisted living facility due to increasing needs for daily assistance.

Medical History:

- Dementia: Mild to moderate Alzheimer's disease, diagnosed 4 years ago.
- Osteoporosis: Multiple vertebral compression fractures, on calcium and vitamin D supplements.
- Hypertension: Well-controlled with medication.
- Gastroesophageal Reflux Disease (GERD): Managed with proton pump inhibitors.

Social History:

- Former seamstress, enjoyed cooking traditional meals for family gatherings.
- No history of smoking or alcohol use.
- Allergic to penicillin.

Current Presentation:Mrs. Rodriguez was brought to the geriatric clinic by the facility's nurse after several episodes where she was found wandering at night, expressing confusion and distress about not being able to find her late husband. She has also shown a decline in her appetite and has lost weight over the past few months. 

Assessment:

- Vitals: BP: 125/75 mmHg, HR: 72 bpm, RR: 18 breaths/min, Temp: 98.2°F, O2 Sat: 97% on room air, Weight: Loss of 8 lbs in 3 months.
- General Appearance: Frail, with evident kyphosis, appears anxious when discussing her late husband.
- Mental Status: Montreal Cognitive Assessment (MoCA) score of 18/30, showing deficits in memory, orientation, and executive function.
- Physical Exam:
- Musculoskeletal: Reduced range of motion due to kyphosis, uses a walker for stability.
- Neurological: No focal deficits, but general slowing of responses.
- Gastrointestinal: Mild abdominal tenderness, likely related to her osteoporosis or GERD.

Medications:

- Donepezil for Alzheimer's disease
- Amlodipine for hypertension
- Omeprazole for GERD
- Calcium and Vitamin D supplements

Psychosocial Factors:-...

# Mrs. Gloria Rodriguez

Patient Demographics:

- Age: 68
- Gender: Female
- Marital Status: Divorced
- Living Situation: Small apartment in a low-income neighborhood
- Children: Two adult sons, estranged
- Medical History: Breast cancer (in remission), Osteoporosis, Anxiety, Chronic Obstructive Pulmonary Disease (COPD)

Presenting Complaint:

Mrs. Rodriguez is referred to the social work department by her oncologist. While Mrs. Rodriguez successfully completed treatment for breast cancer and is currently in remission, she reports increasing difficulty managing her daily activities due to fatigue, shortness of breath (related to COPD), and anxiety about her cancer returning. She is socially isolated and has limited financial resources.

Physical Examination:

- Vital Signs: BP 110/70 mmHg, HR 90 bpm, RR 20, Temp 97.8 F
- General Appearance: Thin, appears anxious, using pursed-lip breathing
- Respiratory: Decreased breath sounds bilaterally, wheezes on auscultation
- Musculoskeletal: Limited range of motion in shoulders due to post-mastectomy pain

Social History:

- Mrs. Rodriguez worked as a seamstress before her illness.
- She has been unable to work since her diagnosis and struggles with financial insecurity.
- She has limited health literacy and difficulty understanding medical information.
- She is estranged from her sons and has minimal social support.
- She reports feeling overwhelmed and hopeless about the future.

Learning Objectives:

- Psychosocial Assessment: Students will need to conduct a thorough psychosocial assessment to identify Mrs. Rodriguez's needs and challenges.
- Chronic Illness and Cancer Survivorship: Students will need to understand the challenges faced by cancer survivors, including physical, emotional, and social impacts.
- Social Determinants of Health: Students will need to identify and address social determinants of health that are affecting Mrs. Rodriguez's well-being, such as poverty, social isolation, and limited health literacy.
- Community Resources: Students will need to connect Mrs. Rodriguez with appropriate community resources, such as support groups, financial assistance programs, and transportation services.
- Patient Advocacy: Students will need to advocate for Mrs. Rodriguez's needs and help her navigate the healthcare system.
- Interprofessional Collaboration: Students from different disciplines will need to work together to develop a comprehensive care plan that addresses Mrs. Rodriguez's physical, emotional, and social needs.

Simulation Activities:

- Patient Interview: Students can conduct a simulated patient interview to practice communication skills and gather information about Mrs. Rodriguez's needs.
- Resource Navigation: Students can research and present information on relevant community resources.
- Interprofessional Case Conference: Students from different disciplines can participate in a case conference to discuss Mrs. Rodriguez's care plan and coordinate services.
- Home Visit: Students can conduct a simulated home visit to assess Mrs. Rodriguez's living environment and provide education and support.
- Support Group Role-Play: Students can role-play a support group meeting to provide Mrs. Rodriguez with an opportunity to connect with others and share her experiences.

Assessment:

- Students can be assessed on their ability to:
- Conduct a comprehensive psychosocial assessment
- Identify and address social determinants of health
- Connect patients with appropriate community resources
- Advocate for patient needs
- Collaborate effectively with other disciplines
- Communicate effectively with patients with limited health literacy

This case study highlights the complex interplay of physical, emotional, and social factors that can affect older adults with chronic illnesses. It provides students with an opportunity to develop the knowledge, skills, and attitudes necessary to provide holistic, patient-centered care to this vulnerable population.

# Ms. Maria Sanchez

Patient Demographics:

- Age: 88
- Gender: Female
- Marital Status: Widowed
- Living Situation: Lives alone in a senior apartment complex
- Children: One son who lives in another state
- Medical History: Congestive Heart Failure (CHF), Hypertension, Type 2 Diabetes, Macular Degeneration, Osteoporosis

Presenting Complaint:

Ms. Sanchez is referred to the geriatric clinic by her primary care physician due to increasing concerns about her safety and well-being. She has a history of multiple falls in the past few months, and her neighbors have reported that she seems confused and disoriented at times. She is also experiencing difficulty managing her medications and adhering to her dietary restrictions.

Physical Examination:

- Vital Signs: BP 150/100 mmHg, HR 100 bpm, RR 20, Temp 98.4 F
- General Appearance: Frail, poorly groomed, evidence of bruising on arms and legs
- Cardiovascular: Elevated heart rate, edema in lower extremities
- Neurological: Disoriented to time and place, difficulty with short-term memory
- Ophthalmological: Significant vision impairment due to macular degeneration

Social History:

- Ms. Sanchez was a homemaker and active in her church community before her health declined.
- She is a devout Catholic and relies on her faith for strength.
- She speaks limited English and prefers to communicate in Spanish.
- She is socially isolated and has limited contact with her son.
- She is resistant to the idea of leaving her apartment and moving to assisted living.

Learning Objectives:

- Comprehensive Geriatric Assessment: Students will need to conduct a comprehensive assessment of Ms. Sanchez's physical, cognitive, functional, and social needs.
- Fall Prevention: Students will need to identify fall risk factors and develop strategies to prevent future falls.
- Medication Management: Students will need to assess Ms. Sanchez's medication adherence and develop a plan to simplify her medication regimen.
- Cognitive Impairment: Students will need to assess Ms. Sanchez's cognitive function and provide education and support to her and her family.
- Cultural and Linguistic Competency: Students will need to demonstrate cultural sensitivity and utilize appropriate communication strategies when interacting with Ms. Sanchez.
- Ethical Considerations: Students will need to address ethical dilemmas related to Ms. Sanchez's autonomy and decision-making capacity.

Simulation Activities:

- Home Safety Assessment: Students can visit a simulated apartment environment to identify potential hazards and make recommendations for modifications.
- Medication Reconciliation and Education: Students can review Ms. Sanchez's medications and provide education in her preferred language.
- Cognitive Assessment and Communication Simulation: Students can practice communication techniques with a standardized patient portraying Ms. Sanchez.
- Family Meeting Role-Play: Students can role-play a family meeting with Ms. Sanchez and her son to discuss her care plan and address concerns.
- Interprofessional Case Conference: Students from different disciplines can collaborate to develop a comprehensive care plan for Ms. Sanchez.

Assessment:

- Students can be assessed on their ability to:
- Conduct a comprehensive geriatric assessment
- Identify and address fall risk factors
- Develop a medication management plan
- Assess and address cognitive impairment
- Demonstrate cultural and linguistic competency
- Address ethical considerations related to patient autonomy and decision-making

This case study provides a valuable opportunity for students to develop the skills necessary to provide culturally competent and patient-centered care to older adults with complex needs.

# Mrs. Emily Smith

Patient Demographics:

- Age: 82
- Gender: Female
- Marital Status: Widowed (husband passed away 5 years ago)
- Living Situation: Lives alone in a two-story home with stairs
- Children: One daughter, lives 2 hours away
- Medical History: Hypertension, Osteoarthritis, Mild Cognitive Impairment, History of falls

Presenting Complaint:

Mrs. Smith is brought to the emergency department by her daughter, who found her confused and disoriented at home. Mrs. Smith has a recent history of falls and has become increasingly forgetful. Her daughter is concerned about her ability to live independently.

Physical Examination:

- Vital Signs: BP 150/90 mmHg, HR 88 bpm, RR 18, Temp 98.6 F
- General Appearance: Appears frail, unsteady gait, some bruising on arms
- Neurological: Mildly disoriented to time and place, difficulty with short-term memory recall
- Musculoskeletal: Limited range of motion in knees and hips due to osteoarthritis

Social History:

- Mrs. Smith was a teacher before retirement.
- She has a limited social support network since her husband passed away.
- She enjoys reading and gardening but has been unable to participate in these activities recently due to her health.
- She is fiercely independent and resistant to the idea of leaving her home.

Learning Objectives:

- Interprofessional Collaboration: Students from different disciplines will need to work together to assess Mrs. Smith's needs and develop a comprehensive care plan.
- Geriatric Assessment: Students will need to perform a comprehensive geriatric assessment, including physical, cognitive, and functional assessments.
- Medication Management: Students will need to review Mrs. Smith's medication list and identify any potential drug interactions or side effects that may be contributing to her symptoms.
- Fall Prevention: Students will need to identify fall risk factors and develop strategies to prevent future falls.
- Cognitive Impairment: Students will need to assess Mrs. Smith's cognitive function and provide education and support to her and her family.
- Social Support and Community Resources: Students will need to identify social support and community resources that can help Mrs. Smith maintain her independence and quality of life.
- Ethical Considerations: Students will need to consider ethical issues related to Mrs. Smith's autonomy and decision-making capacity.

Simulation Activities:

- Home Safety Assessment: Students can visit a simulated home environment to identify potential hazards and make recommendations for modifications.
- Medication Reconciliation: Students can review Mrs. Smith's medication list and identify any potential issues.
- Family Meeting: Students can role-play a family meeting to discuss Mrs. Smith's care plan and address any concerns.
- Community Resource Navigation: Students can research and present information on available community resources.
- Ethical Dilemma Discussion: Students can discuss ethical dilemmas related to Mrs. Smith's care, such as balancing her desire for independence with her safety needs.

Assessment:

- Students can be assessed on their ability to:
- Perform a comprehensive geriatric assessment
- Develop a comprehensive care plan
- Collaborate effectively with other disciplines
- Communicate effectively with the patient and family
- Identify and address ethical considerations

This case study provides a rich learning opportunity for students from various health professional disciplines. By working together in a simulated environment, students can gain valuable experience in providing comprehensive care to older adults.


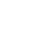


# Mrs. Irene Smith

Patient Profile:

- Name: Irene Smith
- Age: 82
- Gender: Female
- Ethnicity: Hispanic
- Marital Status: Widowed
- Living Situation: Lives alone in a small apartment
- Insurance: Medicare and Medicaid
- Medical History:
- Chronic Heart Failure (diagnosed 5 years ago)
- Type 2 Diabetes Mellitus (diagnosed 12 years ago)
- Osteoarthritis in knees (diagnosed 8 years ago)
- Macular Degeneration (diagnosed 4 years ago)
- History of falls (3 reported in the past year)

Current Medications:

- Metformin 500 mg BID
- Furosemide 40 mg daily
- Lisinopril 20 mg daily
- Acetaminophen 500 mg TID for pain
- Insulin as needed

Chief Complaint: Mrs. Smith presents with increasing shortness of breath, swelling in her legs, and difficulty managing her blood sugar levels.

Social History:

- Retired seamstress
- Has one son who lives out of state and visits monthly
- Enjoys sewing and listening to music but has reduced activity due to health issues
- Active in a local senior center but has decreased participation recently

Physical Examination Findings:

- BP: 150/90 mmHg
- HR: 90 bpm
- RR: 20 breaths/min
- Temp: 98.2°F
- Weight: 160 lbs, Height: 5'3"
- General: Appears tired, mild respiratory distress
- HEENT: No acute distress
- Cardiovascular: Regular rate and rhythm, mild pedal edema
- Respiratory: Clear to auscultation bilaterally
- Musculoskeletal: Decreased range of motion in knees, pain on palpation
- Neurological: Alert and oriented, follows simple commands

Simulation Objectives:

- Medical Students:
- Conduct a comprehensive evaluation of heart failure and diabetes management.
- Develop an appropriate medication regimen and titration plan.
- Address issues related to polypharmacy and fall prevention.
- Nursing Students:
- Perform a thorough physical assessment, focusing on respiratory and cardiovascular function.
- Develop a care plan to manage symptoms of heart failure, diabetes, and fall risk.
- Educate the patient on lifestyle modifications, medication adherence, and fall prevention strategies.
- Social Work Students:
- Assess the impact of Mrs. Smith's health conditions on her daily life and social interactions.
- Identify community resources and support systems for elderly patients with chronic illnesses.
- Collaborate with the healthcare team to address Mrs. Smith's psychosocial needs and improve her quality of life.

Discussion Points:

- Balancing the management of multiple chronic conditions in elderly patients.
- Importance of addressing both physical and mental health in geriatric care.
- Strategies for improving quality of life and reducing hospital readmissions.
- Role of interdisciplinary teamwork in providing comprehensive care.
- This case study provides an opportunity for students from different health disciplines to engage in collaborative, patient-centered care, focusing on the unique challenges and needs of elderly patients with multiple chronic conditions.

# Mr. John Stevens

Patient Profile:

Name: John Stevens

Age: 82 years old

Gender: Male

Ethnicity: Caucasian

Marital Status: Divorced, lives alone

Living Situation: Resides in a small apartment with home health aide assistance 3 times per week

Occupation: Retired mechanic

Insurance: Medicare and VA benefits

Primary Language: English

Advance Directive: Living will with Do Not Resuscitate (DNR) order

Chief Complaint:

Mr. Stevens is brought to the emergency department by his neighbor due to worsening shortness of breath and confusion. He has advanced chronic obstructive pulmonary disease (COPD) and has been experiencing progressively worsening respiratory symptoms over the past month. His neighbor, who helps him occasionally, reports that he has been less responsive, more fatigued, and appears weaker than usual.

Past Medical History:

Advanced COPD (diagnosed 12 years ago)

Coronary artery disease (CAD), status post-stent placement 8 years ago

Hypertension (diagnosed 25 years ago)

Anxiety (diagnosed 10 years ago)

History of smoking (60 pack-years, quit 10 years ago)

Osteoporosis (diagnosed 7 years ago)

Chronic back pain (from degenerative disc disease)

Current Medications:

Albuterol inhaler, as needed (used multiple times daily)

Tiotropium inhaler, once daily

Prednisone 10 mg daily (long-term use for COPD)

Furosemide 20 mg once daily (for fluid management in CAD)

Metoprolol 50 mg twice daily (for CAD and hypertension)

Calcium + Vitamin D supplements

Alprazolam 0.25 mg as needed (for anxiety)

Acetaminophen 500 mg every 6 hours as needed (for back pain)

Social History:

Mr. Stevens is divorced and has two adult children who live in another state. They visit infrequently, with occasional phone contact.

He lives alone in a small apartment but has a home health aide visit three times a week for medication management and light housekeeping.

He has a history of heavy smoking and alcohol use but quit both 10 years ago after his COPD diagnosis.

He is largely homebound due to his COPD, and his mobility is significantly limited by his shortness of breath. He occasionally uses a wheelchair for longer distances.

He expresses fear of dying alone and anxiety about his worsening health but is firm in his wish not to be intubated or resuscitated.

Functional Status:

Mr. Stevens is mostly independent with ADLs but requires assistance with meal preparation, medication management, and household chores.

He experiences significant shortness of breath with minimal exertion and uses home oxygen 24 hours a day.

His activity is limited to moving between his bed and his recliner. He uses a walker indoors but has recently needed more help due to weakness and fatigue.

He has difficulty sleeping due to chronic cough and frequent bouts of shortness of breath.

Review of Systems:

General: Weakness, fatigue, worsening shortness of breath, recent confusion

Cardiovascular: No chest pain or palpitations

Respiratory: Frequent shortness of breath, especially at rest, productive cough with yellow sputum, uses oxygen 24/7

Gastrointestinal: Poor appetite, occasional constipation

Genitourinary: No urinary complaints

Musculoskeletal: Chronic back pain, worsening weakness, limited mobility

Neurological: Recent confusion, occasional headaches, no loss of consciousness

Psychological: Anxiety about his health, fear of dying alone

Physical Examination Findings:

General: Frail, visibly fatigued, appears anxious, pale

Vital signs:

Blood pressure: 110/70 mmHg

Pulse: 85 bpm, regular

Respiratory rate: 26 breaths/min, shallow breathing

Oxygen saturation: 89% on 2L oxygen

Temperature: 100.4°F

Cardiovascular: Distant heart sounds, no murmurs

Lungs: Diminished breath sounds bilaterally, wheezes and crackles in lower lobes, increased work of breathing

Abdomen: Soft, non-tender

Musculoskeletal: Kyphosis, generalized muscle wasting

Skin: Thin, bruises easily, no pressure sores

Lab Results:

Complete Blood Count (CBC): Elevated white blood cell count (14,000/mm³), suggestive of infection

Basic Metabolic Panel (BMP): Normal

Arterial Blood Gas (ABG):

pH: 7.32 (acidosis)

pCO2: 60 mmHg (hypercapnia)

pO2: 55 mmHg (hypoxemia)

Chest X-ray: Bilateral infiltrates consistent with pneumonia, hyperinflation consistent with COPD

Interdisciplinary Learning Objectives:

Medical Students:

Conduct a comprehensive assessment of Mr. Stevens’ advanced COPD and manage his acute pneumonia exacerbation.

Evaluate the impact of long-term steroid use and manage potential complications, such as infections and osteoporosis.

Discuss palliative care options, including symptom management and end-of-life care, in alignment with Mr. Stevens' wishes.

Address his anxiety and confusion, considering his respiratory status and medications.

Nursing Students:

Perform an assessment of Mr. Stevens’ functional status and identify interventions to maintain comfort and reduce his anxiety.

Educate Mr. Stevens and his home health aide on infection control measures and the use of oxygen therapy.

Develop a care plan that includes palliative measures to manage his shortness of breath, fatigue, and anxiety.

Monitor for signs of respiratory failure and infection, and coordinate with the healthcare team to ensure timely interventions.

Social Work Students:

Conduct a psychosocial assessment of Mr. Stevens’ emotional well-being, including his fear of dying alone and anxiety about his health.

Discuss the availability of hospice or in-home palliative care services, considering his advanced COPD and expressed wishes.

Explore potential support networks for Mr. Stevens, including reaching out to his children or identifying community resources.

Facilitate conversations about advanced care planning, including his DNR order and comfort measures at the end of life.

Discussion Questions:

What are the key challenges in managing Mr. Stevens’ advanced COPD and pneumonia? How can the interdisciplinary team balance acute care with his end-of-life wishes?

How would you manage Mr. Stevens’ anxiety and fear of dying, particularly in the context of his respiratory distress and limited social support?

What palliative care options should be considered for Mr. Stevens to improve his quality of life while respecting his DNR order and wish to avoid intubation?

How can the team address potential caregiver strain for the home health aide and provide additional support for Mr. Stevens at home?

How would you facilitate a conversation with Mr. Stevens' children about his current condition, his wishes, and the potential transition to hospice care?

Simulation Objectives:

Develop an interdisciplinary care plan that focuses on both the acute management of pneumonia and the long-term management of advanced COPD.

Practice discussing end-of-life care options, including hospice, with patients and their families in a compassionate and culturally sensitive manner.

Collaborate on strategies to address Mr. Stevens' anxiety, fear, and desire for comfort while adhering to his advance directive and DNR.

Enhance communication between healthcare professionals and caregivers to ensure patient-centered care at the end of life.

This case highlights the complexities of managing advanced COPD and respiratory failure in a patient nearing end-of-life. It emphasizes the need for interdisciplinary teamwork to address acute medical issues while also respecting the patient’s autonomy and preferences for end-of-life care.

# Mr. Haruto Tanaka

Background:

Patient: Mr. Haruto TanakaAge: 90 yearsGender: MaleEthnicity: JapaneseMarital Status: WidowedLiving Situation: Recently moved to his daughter's home in a suburban area after living independently in the city for many years. 

Medical History:

- Chronic Kidney Disease (CKD): Stage 4, requiring careful medication management.
- Prostate Cancer: Underwent radiation therapy 10 years ago, currently in remission, PSA levels stable.
- Cataracts: Both eyes, operated on 3 years ago.
- Hypertension: Controlled with medication.

Social History:

- Former university professor in literature, still enjoys reading translated works into Japanese.
- Lifetime non-smoker, drinks green tea daily but no alcohol.
- Allergic to shellfish.

Current Presentation:Mr. Tanaka was brought to the geriatric clinic by his daughter due to increasing forgetfulness, difficulty in managing his medications, and several incidents of nocturnal enuresis which he finds very distressing and embarrassing. His daughter also mentions he seems more withdrawn and less interested in his books lately.

Assessment:

- Vitals: BP: 130/80 mmHg, HR: 65 bpm, RR: 16 breaths/min, Temp: 98.0°F, O2 Sat: 95% on room air.
- General Appearance: Well-groomed but appears slightly underweight, expressions of discomfort when discussing incontinence.
- Mental Status: Mini-Mental State Examination (MMSE) score of 21/30, showing deficits in memory, especially short-term, and some in orientation.
- Physical Exam:
- Urological: Palpable bladder upon examination, suggesting urinary retention issues.
- Neurological: Normal, except for mild peripheral neuropathy likely from age and CKD.
- Cardiovascular: Regular rate and rhythm, no murmurs.

Medications:

- Lisinopril for hypertension and kidney protection
- Calcium Channel Blocker for additional BP control
- Phosphate binder for CKD

Psychosocial Factors:

- Struggling with the transition from independence to needing assistance, which affects his self-esteem.
- Feels like a burden to his daughter, who has her own family to care for.
- Cultural stigma associated with incontinence adds to his distress.

Interdisciplinary Learning Objectives:

- Medical Students:
- Manage CKD in the context of other comorbidities, particularly focusing on drug dosage adjustments.
- Explore causes of urinary retention or incontinence in the elderly, considering prostate size, medication side effects, or neurological issues.
- Nursing Students:
- Develop a care plan for managing incontinence, including bladder training or catheterization if necessary.
- Educate on medication management strategies for patients with cognitive impairment.
- Social Work Students:
- Address the emotional and psychological impact of losing independence and dealing with stigmatized health issues.
- Facilitate family discussions on care responsibilities, exploring community resources or home health services.
- Interdisciplinary Goals:
- Improve Mr. Tanaka's quality of life through better symptom management, particularly focusing on incontinence and cognitive function.
- Coordinate care to ensure all aspects of his health and well-being are addressed, respecting his cultural background.

Simulation Scenario:

- Setting: Home visit followed by a clinic appointment.

- Tasks:
- Conduct a home environment assessment for safety and modifications needed due to mobility and vision issues.
- Review and adjust medication for CKD, considering his cognitive decline and urinary issues.
- Plan for psychological support to address his feelings...

# Mr. Leonard Thomas

Patient Profile:

- Name: Leonard Thomas
- Age: 76
- Gender: Male
- Ethnicity: African American
- Marital Status: Married
- Living Situation: Lives with his wife in a two-story house
- Insurance: Medicare

Medical History:

- Chronic Heart Failure (diagnosed 8 years ago)
- Type 2 Diabetes Mellitus (diagnosed 10 years ago)
- Chronic Kidney Disease Stage 3 (diagnosed 5 years ago)
- Benign Prostatic Hyperplasia (BPH) (diagnosed 4 years ago)
- Moderate Depression (diagnosed 2 years ago)
- Current Medications:
- Metformin 500 mg BID
- Furosemide 20 mg daily
- Carvedilol 6.25 mg BID
- Tamsulosin 0.4 mg daily
- Sertraline 50 mg daily

Chief Complaint: Mr. Thomas presents with increased shortness of breath, swelling in his legs, and difficulties managing his blood sugar levels.

Social History:

- Retired electrician
- Has three adult children, one of whom lives nearby and visits weekly
- Enjoys woodworking and watching sports but has been less active due to health issues
- Previously active in the community but has decreased participation recently

Physical Examination Findings:

- BP: 138/85 mmHg
- HR: 88 bpm
- RR: 20 breaths/min
- Temp: 98.4°F
- Weight: 190 lbs, Height: 5'10"
- General: Appears fatigued, mild respiratory distress
- HEENT: No acute distress
- Cardiovascular: Regular rate and rhythm, mild pedal edema
- Respiratory: Clear to auscultation bilaterally
- Abdomen: Mild suprapubic tenderness
- Musculoskeletal: Reduced mobility due to leg swelling
- Neurological: Alert and oriented, mild depressive symptoms

Simulation Objectives:

- Medical Students:
- Conduct a comprehensive assessment of heart failure and diabetes management.
- Develop a medication regimen and titration plan to address polypharmacy.
- Evaluate and manage chronic kidney disease in the context of other comorbidities.
- Nursing Students:
- Perform a detailed physical examination focusing on cardiovascular and respiratory function.
- Develop a care plan addressing symptoms of heart failure, diabetes management, and leg swelling.
- Educate the patient on lifestyle modifications, medication adherence, and dietary changes.
- Social Work Students:
- Assess the impact of Mr. Thomas's health conditions on his daily life and family dynamics.
- Identify community resources and support systems for elderly patients with chronic illnesses.
- Collaborate with the healthcare team to address Mr. Thomas's psychosocial needs and improve his quality of life.

Discussion Points:

- Balancing the management of multiple chronic conditions in elderly patients.
- Importance of addressing both physical and mental health in geriatric care.
- Strategies for improving quality of life and reducing hospital readmissions.
- Role of interdisciplinary teamwork in providing holistic care.
- This case study provides an opportunity for students from different health disciplines to engage in collaborative, patient-centered care, focusing on the unique challenges and needs of elderly patients with multiple chronic conditions.

# Mr. Harold Thompson

- Patient Profile:
- Name: Harold Thompson
- Age: 82 years old
- Gender: Male
- Ethnicity: Caucasian
- Marital Status: Widowed (for 5 years)
- Living Situation: Lives alone in a one-story house
- Occupation: Retired factory worker
- Insurance: Medicare
- Primary Language: English
- Advance Directive: None
- Chief Complaint:
- Mr. Thompson is brought to the emergency department by his neighbor after a fall at home. He reports feeling weak and dizzy over the past two weeks. The fall occurred while getting out of bed in the middle of the night to use the bathroom. He denies hitting his head but reports bruising on his right hip and mild soreness in his lower back. He did not seek immediate medical care and waited until morning when his neighbor found him struggling to walk.
- Past Medical History:
- Hypertension (diagnosed 15 years ago)
- Type 2 Diabetes (diagnosed 10 years ago)
- Mild cognitive impairment (diagnosed 1 year ago)
- Osteoarthritis (knees, diagnosed 5 years ago)
- Benign prostatic hyperplasia (BPH)
- Hearing loss (uses hearing aids)
- Current Medications:
- Metformin 500 mg twice daily
- Lisinopril 10 mg once daily
- Ibuprofen 400 mg as needed for knee pain
- Acetaminophen 650 mg as needed
- Tamsulosin 0.4 mg once daily
- Multivitamin daily
- Social History:
- Mr. Thompson has been widowed for five years. His wife was his primary caregiver before her death.
- He has one daughter, Sarah, who lives out of state and visits twice a year. He communicates with her by phone weekly.
- His social support network consists of a neighbor who checks in on him and a church group he attends every other Sunday.
- He reports no history of alcohol or tobacco use.
- He occasionally forgets to take his medications and relies on a pill organizer.
- Functional Status:
- Independent with activities of daily living (ADLs), but reports difficulty with instrumental activities of daily living (IADLs), such as grocery shopping and managing finances.
- He uses a cane to walk due to knee pain from osteoarthritis.
- Recently, he has been experiencing increased difficulty rising from a seated position and walking long distances.
- Reports needing to get up multiple times at night to urinate (due to BPH).
- Nutrition:
- Mr. Thompson reports a poor appetite over the past month. He eats two small meals a day and has unintentionally lost 5 pounds in the last three months.
- He consumes processed foods and frozen meals due to difficulty cooking.
- Review of Systems:
- General: Weight loss, fatigue, dizziness, occasional confusion
- Cardiovascular: No chest pain or palpitations
- Respiratory: No shortness of breath or cough
- Gastrointestinal: Regular bowel movements, no diarrhea or constipation
- Genitourinary: Frequent urination, especially at night
- Musculoskeletal: Knee pain, difficulty walking, and recent fall
- Neurological: Occasional forgetfulness, no loss of consciousness, dizziness
- Psychological: Feels lonely, denies depression, occasional anxiety about falling again
- Physical Examination Findings:
- General: Thin, frail appearance
- Vital signs:
- Blood pressure: 130/85 mmHg
- Pulse: 72 bpm, regular
- Respiratory rate: 16 breaths/min
- Oxygen saturation: 96% on room air
- Temperature: 98.0°F
- Head and neck: No trauma or tenderness
- Cardiovascular: No murmurs, regular rate and rhythm
- Lungs: Clear to auscultation bilaterally
- Abdomen: Soft, non-tender, no masses
- Musculoskeletal: Mild tenderness over the right hip, reduced range of motion in both knees due to osteoarthritis
- Neurological: Oriented to person but confused about the date and place; otherwise, no focal deficits
- Skin: Bruising on right hip and lower back, skin is thin with poor turgor
- Lab Results:
- Complete Blood Count (CBC): Normal
- Basic Metabolic Panel (BMP): Slightly elevated creatinine (1.4 mg/dL), normal electrolytes
- Hemoglobin A1c: 7.8%
- Urinalysis: Negative for infection, glucose present
- X-rays of hip and back: No fractures
- Interdisciplinary Learning Objectives:
- Medical Students:
- Conduct a comprehensive geriatric assessment, including falls risk and cognitive evaluation.
- Identify and manage polypharmacy concerns in the elderly.
- Develop a plan for managing Mr. Thompson's chronic conditions (hypertension, diabetes, BPH).
- Evaluate the need for further diagnostic testing or referrals (e.g., neurology for cognitive impairment).
- Nursing Students:
- Assess Mr. Thompson’s functional status and identify interventions to prevent falls.
- Provide education on safe medication management, nutrition, and diabetes self-care.
- Develop a care plan to monitor blood glucose levels, manage incontinence, and encourage mobility.
- Discuss discharge planning and home safety modifications to prevent future falls.
- Social Work Students:
- Evaluate Mr. Thompson’s social support network and identify gaps in caregiving.
- Explore community resources, such as Meals on Wheels and home health services.
- Discuss advance care planning, including creating a living will and appointing a healthcare proxy.
- Address potential elder abuse or neglect if applicable, and discuss ways to improve his social isolation and mental health.
- Discussion Questions:
- What are the key risk factors that led to Mr. Thompson’s fall? How can the interdisciplinary team work together to reduce these risks?
- What is the role of the social worker in addressing Mr. Thompson’s isolation and mental health needs?
- How would you manage Mr. Thompson’s polypharmacy and ensure he understands his medications?
- What are the most appropriate next steps in managing his mild cognitive impairment?
- How should the team approach the conversation about advance directives and long-term care planning?
- Simulation Objectives:
- Develop team-based care plans that integrate medical, nursing, and social work perspectives.
- Enhance communication among health professionals for comprehensive patient care.
- Practice culturally sensitive care and shared decision-making.
- This case study can be adapted to different levels of learners, focusing on their roles in the interdisciplinary care of elderly patients like Mr. Thompson.

# Mrs. Eleanor Thompson

Patient Information

- Name: Eleanor Thompson
- Age: 78
- Gender: Female
- Marital Status: Widowed (2 years ago)
- Living Situation: Lives alone in a two-story house

Chief Complaint

"I fell in my garden yesterday and now my hip hurts. I don't want to be a bother, but my daughter insisted I come in."

History of Present Illness

Mrs. Thompson fell while gardening yesterday afternoon. She reports landing on her right side and experiencing immediate pain in her right hip. She managed to get up on her own after about 10 minutes and went inside to rest. The pain has persisted and worsened overnight, making it difficult for her to walk or put weight on her right leg.

Past Medical History

- Hypertension (diagnosed 10 years ago)
- Type 2 Diabetes Mellitus (diagnosed 8 years ago)
- Osteoarthritis (knees and hands)
- Mild cognitive impairment (diagnosed 1 year ago)

Medications

- Lisinopril 10mg daily
- Metformin 500mg twice daily
- Acetaminophen 500mg as needed for pain
- Calcium + Vitamin D supplement daily

Allergies

- Penicillin (rash)

Social History

- Retired elementary school teacher
- Widowed 2 years ago; husband died of heart attack
- Two adult children: daughter lives 30 minutes away, son lives in another state
- Enjoys gardening and weekly bridge club with friends
- Doesn't drink alcohol or smoke

Review of Systems

- General: Fatigue, no recent weight changes
- Cardiovascular: No chest pain or palpitations
- Respiratory: No shortness of breath or cough
- Gastrointestinal: Occasional constipation
- Genitourinary: Occasional stress incontinence
- Musculoskeletal: Chronic knee and hand pain from osteoarthritis
- Neurological: Occasionally forgets to take medications, some difficulty with short-term memory

Physical Examination

- Vital Signs:
- BP: 145/85 mmHg
- HR: 82 bpm
- RR: 18/min
- Temp: 37.0°C (98.6°F)
- O2 Sat: 97% on room air
- General: Alert, oriented to person and place, but unsure of exact date
- Cardiovascular: Regular rate and rhythm, no murmurs
- Respiratory: Clear to auscultation bilaterally
- Abdomen: Soft, non-tender, no masses
- Musculoskeletal:
- Right hip: Tender to palpation, pain with passive and active movement
- Decreased range of motion in right hip
- Unable to bear full weight on right leg
- Neurological: Cranial nerves intact, 5/5 strength in upper extremities and left lower extremity, 4/5 strength in right lower extremity due to pain

Laboratory and Imaging Results

- Complete Blood Count (CBC): Within normal limits
- Basic Metabolic Panel (BMP):
- Glucose: 142 mg/dL (slightly elevated)
- Creatinine: 1.1 mg/dL (mildly elevated)
- X-ray of right hip: Reveals a non-displaced fracture of the right femoral neck

Assessment

- Right femoral neck fracture
- Hypertension, poorly controlled
- Type 2 Diabetes Mellitus
- Osteoarthritis
- Mild cognitive impairment
- Fall risk
- Potential medication management issues

Plan

- Orthopedic consultation for surgical evaluation and management of hip fracture
- Pain management: Start with IV morphine for acute pain, transition to oral pain medication as tolerated
- DVT prophylaxis
- Physical therapy and occupational therapy evaluations
- Reassess and adjust hypertension and diabetes management
- Geriatric consultation for comprehensive geriatric assessment
- Social work consultation for discharge planning and home safety evaluation
- Nutrition consultation for diabetes management and overall nutritional status
- Consider cognitive assessment to further evaluate mild cognitive impairment

Simulation Learning Objectives

- Medical Students:
- Diagnose and manage acute hip fracture in an elderly patient
- Recognize and address multiple comorbidities in geriatric patients
- Develop a comprehensive treatment plan considering the patient's overall health status
- Nursing Students:
- Conduct a thorough geriatric nursing assessment
- Implement fall prevention strategies
- Manage pain in elderly patients
- Assist with medication reconciliation and management
- Social Work Students:
- Assess the patient's social support system and living situation
- Identify potential barriers to discharge and develop a comprehensive discharge plan
- Evaluate the need for additional home services or alternative living arrangements
- Provide resources for caregiver support
- Interdisciplinary Objectives:
- Practice effective communication between healthcare disciplines
- Collaborate to develop a holistic care plan addressing medical, functional, and psychosocial needs
- Recognize and address potential ethical issues in geriatric care (e.g., autonomy vs. safety)

Simulation Scenario Progression

- Initial Assessment: Students from each discipline conduct their respective assessments and share findings.
- Team Huddle: Interdisciplinary team meets to discuss findings and develop a care plan.
- Family Meeting: Simulate a meeting with Mrs. Thompson's daughter to discuss care options and address concerns.
- Discharge Planning: Team collaborates to create a comprehensive discharge plan, considering Mrs. Thompson's medical needs, cognitive status, and living situation.
- Follow-up: Simulate a follow-up appointment 2 weeks post-discharge to assess recovery and adjust the care plan as needed.

This case study provides multiple learning opportunities for students across healthcare disciplines, encouraging collaboration and holistic patient care in a geriatric setting.

# Mr. Edward Thompson

Background:

Patient: Mr. Edward Thompson Age: 84 years Gender: Male Ethnicity: Caucasian Marital Status: Widow Living Situation: Lives alone in a two-story house, with a daughter living 30 miles away who visits weekly. 

Medical History:

- Hypertension: Diagnosed 20 years ago, managed with medication.
- Type 2 Diabetes: Diagnosed 10 years ago, managed with oral hypoglycemics and diet.
- Osteoarthritis: Chronic, affecting knees and hips, mobility increasingly limited.
- Chronic Obstructive Pulmonary Disease (COPD): Diagnosed 5 years ago, uses an inhaler.
- Previous Stroke: 2 years ago, left with mild right-sided weakness.

Social History:

- Retired engineer, used to be very active, enjoyed gardening and reading.
- Smoked for 40 years, quit 15 years ago.
- Drinks occasionally, 1-2 glasses of wine per week.
- No known drug allergies but reports sensitivity to NSAIDs causing stomach upset.

Current Presentation: Mr. Thompson was brought to the clinic by his daughter after she noticed he seemed more confused than usual during her last visit. He has also been less compliant with his diabetes management, leading to elevated glucose levels.

Assessment:

- Vitals: BP: 150/85 mmHg, HR: 78 bpm, RR: 22 breaths/min, Temp: 98.6°F, O2 Sat: 94% on room air.
- General Appearance: Thin, occasionally confused, slightly dyspneic at rest.
- Mental Status: Mini-Mental State Examination (MMSE) score of 23/30, showing deficits in orientation to time and place, and reduced recall.
- Physical Exam:
- Cardiovascular: Slight irregular heart rhythm, no murmurs.
- Respiratory: Prolonged expiration, decreased breath sounds at bases.
- Musculoskeletal: Limited range of motion in knees, difficulty with stairs noted.
- Neurological: Mild right-sided weakness, slow gait, unsteady.

Medications:

- Metformin
- Lisinopril
- Aspirin
- Salbutamol inhaler
- Ipratropium bromide inhaler
- Losartan for blood pressure control

Psychosocial Factors:

- Feels isolated, misses his wife.
- Worried about becoming a burden to his daughter.
- Has some financial concerns about covering healthcare costs.

Interdisciplinary Learning Objectives:

- Medical Students:
- Understand the complexities of polypharmacy in the elderly.
- Manage comorbid conditions like COPD and diabetes in the context of cognitive decline.
- Nursing Students:
- Develop a care plan for managing chronic conditions in home settings.
- Educate on medication adherence and symptom monitoring.
- Social Work Students:
- Assess the patient’s living situation for safety and support needs.
- Explore community resources for elderly care, including possible need for part-time care or moving to assisted living.
- Interdisciplinary Goals:
- Coordinate care to ensure medication management, nutritional needs, and physical therapy are addressed holistically.
- Discuss ethical considerations regarding autonomy versus safety, especially with cognitive decline.

Simulation Scenario:

- Setting: A home visit followed by a clinic appointment.
- Tasks:
- Conduct a comprehensive geriatric assessment.
- Address immediate medical issues (e.g., confusion, high glucose levels).
- Discuss lifestyle modifications and safety at home.
- Plan for ongoing care, considering psychological and social needs.

Simulation Outcome:

Students should identify the need for a fall risk assessment, suggest modifications to the home environment, recommend a review of medications with a geriatric pharmacist, and initiate discussions on end-of-life wishes and living arrangements.

This case study provides a realistic scenario for students to engage in critical thinking, communication, and collaborative practice, essential for managing complex geriatric cases effectively.

# Mr. James "Jim" Thompson

Patient Demographics:

- Age: 79
- Gender: Male
- Marital Status: Married
- Living Situation: Lives with wife in a rural farmhouse
- Children: Three adult children, all living out of state
- Medical History: Parkinson's Disease, Hypertension, History of Depression, Recent diagnosis of Prostate Cancer

Presenting Complaint:

Mr. Thompson is brought to his primary care physician by his wife who is concerned about his increasing social withdrawal, worsening tremors, and difficulty with mobility. Mr. Thompson is resistant to the visit and expresses frustration with his declining health and the side effects of his Parkinson's medications.

Physical Examination:

- Vital Signs: BP 140/90 mmHg, HR 80 bpm, RR 16, Temp 98.2 F
- General Appearance: Stooped posture, tremors in hands and legs, shuffling gait
- Neurological: Bradykinesia, rigidity, masked facies, mild cognitive impairment
- Genitourinary: Reports increased urinary frequency and urgency

Social History:

- Mr. Thompson was a farmer and continues to help with chores despite his limitations.
- He is a proud and independent man who values self-reliance.
- He enjoys spending time outdoors and with his dogs.
- He is struggling to accept his diagnosis of prostate cancer and has not yet decided on a treatment plan.
- His wife is his primary caregiver and is feeling overwhelmed by his increasing care needs.

Learning Objectives:

- Parkinson's Disease Management: Students will need to assess Mr. Thompson's Parkinson's symptoms and develop a plan to manage his motor and non-motor symptoms.
- Medication Management: Students will need to review his medication list and address potential side effects and interactions.
- Decision-Making in Cancer Care: Students will need to explore Mr. Thompson's understanding of his prostate cancer diagnosis and support him in making informed treatment decisions.
- Rural Health Challenges: Students will need to consider the unique challenges faced by older adults living in rural areas, such as access to specialized care and support services.
- Caregiver Support: Students will need to assess Mrs. Thompson's needs and provide education and resources to support her in her caregiving role.
- Communication and Empathy: Students will need to practice effective communication strategies when interacting with Mr. Thompson, considering his personality and emotional state.

Simulation Activities:

- Home Visit Simulation: Students can conduct a home visit to assess Mr. Thompson's living environment and functional abilities.
- Medication Review and Education: Students can review Mr. Thompson's medications and provide education on potential side effects and management strategies.
- Shared Decision-Making Role Play: Students can engage in role-play to practice discussing treatment options for prostate cancer with Mr. Thompson and his wife.
- Caregiver Support Group Simulation: Students can participate in a simulated support group for caregivers of individuals with Parkinson's Disease.
- Interprofessional Case Conference: Students from different disciplines can collaborate to develop a comprehensive care plan for Mr. Thompson.

Assessment:

- Students can be assessed on their ability to:
- Assess and manage Parkinson's Disease symptoms
- Address medication-related issues
- Facilitate shared decision-making in cancer care
- Understand the challenges of rural health
- Provide support and resources to caregivers
- Communicate effectively with patients experiencing emotional distress

This case study provides a valuable opportunity for students to develop the skills necessary to provide comprehensive and compassionate care to older adults with complex needs in a rural setting.

# Mrs. Patricia “Pat” Thompson

Patient Profile:

Name: Patricia “Pat” Thompson

Age: 74 years old

Gender: Female

Ethnicity: Caucasian

Marital Status: Married for 40 years

Living Situation: Lives with her husband in their home

Occupation: Retired school teacher

Insurance: Medicare

Primary Language: English

Advance Directive: Yes, wants comfort measures only if condition is irreversible

Chief Complaint:

Mrs. Thompson is admitted to the inpatient rehabilitation unit following a recent ischemic stroke that left her with right-sided weakness (hemiparesis) and speech difficulties (mild aphasia). Her family is supportive, but her husband expresses concern over her increasing sadness and withdrawal since the stroke. Mrs. Thompson herself feels overwhelmed by her limitations and is fearful about losing her independence.

Past Medical History:

Ischemic stroke (2 weeks ago)

Hypertension (diagnosed 15 years ago)

Type 2 Diabetes Mellitus (diagnosed 10 years ago)

Hyperlipidemia (diagnosed 10 years ago)

Depression (diagnosed 5 years ago, in remission until stroke)

History of transient ischemic attacks (TIAs) (2 years ago)

Current Medications:

Aspirin 81 mg once daily (post-stroke prevention)

Atorvastatin 40 mg once daily (for hyperlipidemia)

Lisinopril 10 mg once daily (for hypertension)

Metformin 500 mg twice daily (for diabetes)

Sertraline 50 mg once daily (for depression)

Multivitamin daily

Insulin glargine 10 units nightly (for diabetes)

Social History:

Mrs. Thompson lives with her husband in a one-story home. She is largely independent with ADLs but now requires assistance due to her stroke.

She and her husband have two adult children who live nearby and are involved in her care, visiting regularly.

Mrs. Thompson enjoys reading and gardening but has been unable to participate in these activities since her stroke, which has contributed to her feelings of sadness and frustration.

She quit smoking 20 years ago and does not drink alcohol.

Functional Status:

Before the stroke, Mrs. Thompson was fully independent in ADLs and walked regularly with her husband for exercise.

Since her stroke, she has right-sided hemiparesis, requiring assistance with walking, bathing, and dressing. She uses a wheelchair for mobility but is working with physical therapy to regain strength.

She has mild expressive aphasia, making it difficult for her to communicate her needs, though she can comprehend most speech and respond with short phrases or gestures.

She expresses fear about becoming a burden to her family and is hesitant to participate in rehabilitation exercises.

Review of Systems:

General: Feels tired, weak, and frustrated with her current condition

Cardiovascular: No chest pain or palpitations, controlled hypertension

Respiratory: No cough or shortness of breath

Gastrointestinal: Appetite reduced, occasional constipation

Genitourinary: No urinary complaints, no incontinence

Musculoskeletal: Right-sided weakness, limited mobility

Neurological: Right-sided hemiparesis, mild expressive aphasia, no recurrent stroke symptoms

Psychological: Feels sad, withdrawn, tearful at times; verbalizes fears of dependence and being a burden

Physical Examination Findings:

General: Thin, frail appearance; sitting in a wheelchair, visibly anxious

Vital signs:

Blood pressure: 140/85 mmHg

Pulse: 78 bpm, regular

Respiratory rate: 16 breaths/min

Oxygen saturation: 97% on room air

Temperature: 98.2°F

Cardiovascular: Regular heart sounds, no murmurs

Lungs: Clear to auscultation bilaterally

Abdomen: Soft, non-tender, no masses

Musculoskeletal: Right-sided hemiparesis with decreased strength (2/5) in the right arm and leg, limited range of motion

Neurological: Mild expressive aphasia, intact comprehension; no new neurological deficits

Skin: No breakdown, pressure areas intact

Lab Results:

Complete Blood Count (CBC): Normal

Basic Metabolic Panel (BMP): Normal, glucose elevated at 150 mg/dL (fasting)

Lipid Panel: LDL 130 mg/dL, HDL 40 mg/dL

HbA1c: 7.5% (slightly elevated)

Interdisciplinary Learning Objectives:

Medical Students:

Perform a thorough neurological assessment and develop a rehabilitation plan for Mrs. Thompson’s post-stroke hemiparesis and aphasia.

Review her current medication regimen and assess for any necessary adjustments, particularly regarding secondary stroke prevention, hypertension, and diabetes management.

Address her concerns about depression and the emotional impact of the stroke, considering adjustments to her treatment for depression.

Evaluate her stroke risk factors and develop strategies to reduce the risk of future strokes.

Nursing Students:

Assess Mrs. Thompson’s functional ability, focusing on her ADLs, mobility, and communication needs.

Provide education and support for her rehabilitation exercises, encouraging participation in physical therapy and speech therapy.

Monitor her nutritional intake, considering her reduced appetite, and develop strategies to prevent weight loss and malnutrition.

Address her psychosocial needs, focusing on her emotional well-being and fears of dependence, and offer strategies to reduce caregiver strain for her husband and family.

Social Work Students:

Conduct a psychosocial assessment of Mrs. Thompson and her family, exploring the emotional impact of the stroke on both the patient and her caregivers.

Facilitate conversations about long-term care planning, including advance directives and the potential need for home health care or rehabilitation services.

Discuss strategies for coping with depression and loss of independence, including counseling, support groups, and resources for both the patient and her family.

Explore family dynamics, ensuring that all members have a voice in Mrs. Thompson’s care and addressing any concerns about caregiver strain or family conflict.

Discussion Questions:

How can the interdisciplinary team best support Mrs. Thompson’s stroke recovery, balancing her physical rehabilitation with her emotional needs?

What are the key factors that need to be considered when managing Mrs. Thompson’s chronic conditions (hypertension, diabetes, hyperlipidemia) alongside her stroke rehabilitation?

How should the team address Mrs. Thompson’s depression, particularly in the context of her fears of losing independence and becoming a burden to her family?

What strategies can be implemented to prevent caregiver burnout for Mrs. Thompson’s husband and children, and what resources might help them manage her care at home?

How would the team facilitate conversations about advance care planning, ensuring that Mrs. Thompson’s wishes are respected as her health evolves?

Simulation Objectives:

Develop an interdisciplinary care plan that addresses Mrs. Thompson’s stroke rehabilitation, emotional well-being, and chronic disease management, with a focus on patient-centered care.

Practice communication strategies to encourage patient engagement in rehabilitation while acknowledging and addressing her emotional concerns about dependence and burden.

Explore ways to prevent caregiver burnout, emphasizing family dynamics and the importance of supporting caregivers alongside the patient.

Facilitate conversations about advance care planning, including discussions about long-term care needs and ensuring that Mrs. Thompson’s preferences for her care are respected.

This case study focuses on the challenges of stroke recovery, the emotional impact of losing independence, and the importance of family dynamics in caregiving. It encourages interdisciplinary collaboration to support Mrs. Thompson’s physical, emotional, and social well-being during her recovery process.

# Mr. Samuel “Sam” Wilson

Patient Profile:

Name: Samuel “Sam” Wilson

Age: 80 years old

Gender: Male

Ethnicity: African American

Marital Status: Widowed for 3 years

Living Situation: Lives alone in his home

Occupation: Retired school principal, U.S. Army veteran

Insurance: Medicare and VA benefits

Primary Language: English

Advance Directive: None documented, but previously expressed a desire to “not be kept alive on machines.”

Chief Complaint:

Mr. Wilson presents to the outpatient clinic with worsening fatigue, shortness of breath, and swelling in his legs over the past two weeks. He admits to feeling “down” since the death of his wife three years ago and says he has become more withdrawn from social activities. He reports difficulty managing his medications and forgets to take them regularly.

Past Medical History:

Congestive Heart Failure (CHF) (diagnosed 5 years ago)

Atrial Fibrillation (diagnosed 3 years ago)

Hypertension (diagnosed 20 years ago)

Type 2 Diabetes Mellitus (diagnosed 15 years ago)

Depression (diagnosed 2 years ago, following wife’s death)

Chronic kidney disease (Stage 3)

History of smoking (30 pack-years, quit 15 years ago)

Current Medications:

Furosemide 40 mg once daily (for CHF)

Metoprolol 50 mg twice daily (for atrial fibrillation and hypertension)

Warfarin 5 mg once daily (for atrial fibrillation)

Lisinopril 10 mg once daily (for hypertension)

Metformin 500 mg twice daily (for diabetes)

Sertraline 50 mg once daily (for depression)

Multivitamin daily

Aspirin 81 mg once daily

Social History:

Mr. Wilson was married for 50 years before his wife passed away. Since then, he has become socially isolated and spends most of his days watching television or sitting alone on his porch.

He has two adult children, but both live out of state and visit infrequently. They call occasionally but are not involved in his day-to-day care.

He is a retired school principal and Army veteran, but he no longer attends the local veterans’ group meetings due to fatigue and loss of interest.

He stopped smoking 15 years ago but was a heavy smoker for most of his adult life.

Functional Status:

Mr. Wilson reports difficulty with activities of daily living (ADLs), especially bathing and dressing, due to fatigue and shortness of breath.

He has been increasingly sedentary and walks with a cane. He has also experienced two falls in the past year, though without injury.

He lives alone and manages his medications independently but admits to often forgetting to take them or taking them at the wrong times.

His diet consists mainly of processed foods, and he has unintentionally lost 10 pounds over the past six months.

Review of Systems:

General: Fatigue, weight loss, decreased appetite

Cardiovascular: Shortness of breath on exertion, worsening peripheral edema, no chest pain

Respiratory: No cough, but reports mild wheezing when lying down

Gastrointestinal: Poor appetite, occasional constipation

Genitourinary: No urinary complaints, but reports decreased urine output

Musculoskeletal: Weakness, generalized muscle aches, occasional joint stiffness

Neurological: No headaches, dizziness, or syncope

Psychological: Reports feeling depressed, socially isolated, low motivation

Physical Examination Findings:

General: Thin, frail-appearing, slow to respond but cooperative

Vital signs:

Blood pressure: 135/85 mmHg

Pulse: 90 bpm, irregular

Respiratory rate: 22 breaths/min

Oxygen saturation: 94% on room air

Temperature: 97.8°F

Cardiovascular: Irregular heart rhythm, 2+ pitting edema in bilateral lower extremities

Lungs: Crackles at the bases bilaterally, mild wheezing on exhalation

Abdomen: Soft, non-tender, no organomegaly

Musculoskeletal: Generalized weakness, mild muscle atrophy in arms and legs

Skin: Dry, no rashes or ulcers

Lab Results:

Complete Blood Count (CBC): Mild anemia (Hemoglobin 11.0 g/dL)

Basic Metabolic Panel (BMP): Elevated creatinine (1.8 mg/dL), potassium 5.2 mEq/L (high), glucose 140 mg/dL (fasting)

BNP (B-type Natriuretic Peptide): Elevated at 550 pg/mL (indicative of heart failure)

INR: 2.3 (within target range for warfarin)

HbA1c: 7.5% (slightly elevated)

Interdisciplinary Learning Objectives:

Medical Students:

Assess Mr. Wilson’s heart failure and atrial fibrillation, considering his worsening symptoms and elevated BNP.

Evaluate the impact of his polypharmacy on his kidney function, blood pressure, and overall health, and adjust his medication regimen as necessary.

Address his poorly controlled diabetes and assess for potential cardiovascular complications.

Explore potential treatments for his depression and social isolation, with a focus on holistic, patient-centered care.

Nursing Students:

Perform a functional assessment to evaluate Mr. Wilson’s ability to manage ADLs and his risk for falls.

Educate Mr. Wilson on medication adherence, including strategies for managing multiple medications and the risks of missing doses.

Develop a care plan that addresses his heart failure symptoms, particularly his edema and shortness of breath, while considering his renal function.

Monitor his mental health and explore interventions to reduce his depression and improve his social engagement.

Social Work Students:

Conduct a psychosocial assessment to explore the impact of social isolation, depression, and grief on Mr. Wilson’s quality of life.

Discuss potential community resources, such as veterans’ groups, home health aides, or senior centers, to help address his isolation and provide support for his ADLs.

Facilitate conversations about advance care planning, including documenting his wishes regarding resuscitation and long-term care options.

Explore family dynamics and the possibility of involving Mr. Wilson’s children more actively in his care, even from a distance.

Discussion Questions:

How can the healthcare team address Mr. Wilson’s worsening heart failure and ensure that his medication regimen is optimized for his cardiovascular and renal health?

What interventions could be put in place to address Mr. Wilson’s depression and social isolation, particularly considering his history as a widower and veteran?

How can the interdisciplinary team help Mr. Wilson manage his multiple medications, particularly in light of his reported difficulties with adherence?

How should the team approach conversations with Mr. Wilson about advance care planning, including his preferences for end-of-life care and resuscitation?

What are some ways to ensure Mr. Wilson receives adequate support at home, given his physical limitations and social isolation?

Simulation Objectives:

Develop an interdisciplinary care plan that focuses on the management of Mr. Wilson’s heart failure, atrial fibrillation, and depression while considering his social isolation and frailty.

Practice patient-centered communication, especially in discussing sensitive topics such as grief, isolation, and advance care planning.

Collaborate on strategies to improve medication adherence in a patient with multiple chronic conditions and limited social support.

Explore community resources and support systems that can enhance Mr. Wilson’s quality of life and reduce his isolation, including veterans’ services and home health care.

This case study emphasizes the complexities of managing heart failure, polypharmacy, depression, and social isolation in an elderly patient, particularly one who is a widower and veteran. It encourages students to consider both the medical and psychosocial aspects of care in a comprehensive, interdisciplinary manner.

# Mrs. Clara Winston

Background:

Patient: Mrs. Clara Winston Age: 78 years Gender: Female Ethnicity: African American Marital Status: Married Living Situation: Lives with her husband in a single-story home in an urban area. Their children live out of state.

Medical History:

- Heart Failure: Diagnosed 6 years ago, NYHA Class II, managed with diuretics and beta-blockers.
- Atrial Fibrillation: Controlled with anticoagulants and rate-controlling medications.
- Hypothyroidism: Stable, treated with levothyroxine.
- Glaucoma: Managed with eye drops.
- Depression: Intermittent episodes, currently not on medication but has a history of SSRIs.

Social History:

- Former high school teacher, very involved in community activities before her health declined.
- Non-smoker, occasional alcohol (1 drink on social occasions).
- No known drug allergies but has a sensitivity to sulfa drugs.

Current Presentation: Mrs. Winston was brought to the emergency department by her husband after she experienced a fall at home, resulting in a minor head injury and left arm pain, which she initially thought was due to the fall. However, she also reports increasing fatigue and shortness of breath over the last few days. 

Assessment:

- Vitals: BP: 145/90 mmHg, HR: 95 bpm (irregular), RR: 20 breaths/min, Temp: 98.4°F, O2 Sat: 92% on room air.
- General Appearance: Anxious, slightly pale, with signs of recent weight gain and edema in both legs.
- Mental Status: Oriented to person, place, and time, but visibly distressed by her current condition.
- Physical Exam:
- Cardiovascular: Jugular venous distension, bilateral leg edema.
- Respiratory: Fine crackles in lung bases, labored breathing.
- Musculoskeletal: Left arm swelling and tenderness, possible fracture or severe sprain.
- Neurological: Normal except for mild tremor, possibly related to anxiety or medication side effects.

Medications:

- Furosemide
- Metoprolol
- Warfarin
- Levothyroxine
- Brimonidine eye drops

Psychosocial Factors:

- Mrs. Winston has been increasingly worried about her husband's ability to care for her if her condition worsens.
- She feels a loss of independence and is fearful of future falls.
- Financial concerns are minimal due to a good retirement plan, but she's anxious about medical expenses.

Interdisciplinary Learning Objectives:

- Medical Students:
- Diagnose and manage acute exacerbation of heart failure.
- Consider differential diagnoses for falls in the elderly, including stroke, heart issues, or medication side effects.
- Nursing Students:
- Develop a care plan focusing on fluid management, fall prevention, and medication management for heart failure patients.
- Teach self-care techniques for monitoring heart failure symptoms at home.
- Social Work Students:
- Assess the need for home modifications or assistive devices for safety.
- Support the couple in exploring long-term care options or in-home nursing services.
- Interdisciplinary Goals:
- Coordinate care to address immediate medical issues while planning for long-term management of chronic conditions.
- Discuss psychological support for coping with chronic illness and declining health.

Simulation Scenario:

- Setting: Emergency Department visit followed by a hospital admission.
- Tasks:
- Conduct an initial assessment and stabilize patient.
- Address immediate medical concerns (e.g., possible arm fracture, heart failure exacerbation).
- Plan for discharge including home care, medication adjustment, and follow-up appointments.
- Discuss lifestyle changes to prevent future exacerbations and falls.

Simulation Outcome:

Students should coordinate a multidisciplinary approach involving physiotherapy for mobility, cardiology consultation for heart failure management, and social services for home safety evaluations. They should also explore mental health support for dealing with her anxiety and depression.

This case study allows students to practice acute care management, chronic disease management, and the importance of holistic care in geriatrics, focusing on both immediate health crises and long-term planning.

# Mrs. Eleanor Wright

Patient Demographics:

- Age: 91
- Gender: Female
- Marital Status: Widowed
- Living Situation: Resides in a nursing home
- Children: One daughter who lives nearby, estranged son
- Medical History: Advanced Dementia, Osteoarthritis, Hypertension, History of Stroke (with residual left-sided weakness), Malnutrition

Presenting Complaint:

Mrs. Wright is experiencing increasing agitation and behavioral disturbances, including verbal and physical aggression towards staff. She has also been refusing meals and has lost a significant amount of weight in recent weeks. Her daughter is distressed by these changes and feels overwhelmed with guilt and grief.

Physical Examination:

- Vital Signs: BP 110/70 mmHg, HR 72 bpm, RR 16, Temp 97.6 F
- General Appearance: Thin, appears frail, contractures in her left hand, pressure ulcer developing on her sacrum
- Neurological: Limited communication, unable to follow commands, appears agitated and distressed
- Musculoskeletal: Limited mobility due to osteoarthritis and stroke-related weakness

Social History:

- Mrs. Wright was a librarian and enjoyed reading, gardening, and spending time with her family.
- She has a history of depression that worsened after her husband's death.
- Her relationship with her daughter is strained due to past conflicts.
- She has limited social interaction with other residents in the nursing home.

Learning Objectives:

- Dementia Care and Behavioral Management: Students will need to assess Mrs. Wright's behavioral disturbances and develop strategies to manage her agitation and aggression.
- Palliative Care and End-of-Life Considerations: Students will need to recognize the signs of advanced dementia and discuss end-of-life care options with Mrs. Wright's daughter.
- Nutrition and Hydration: Students will need to address Mrs. Wright's malnutrition and develop a plan to ensure adequate nutrition and hydration.
- Wound Care: Students will need to assess and manage the pressure ulcer.
- Family Dynamics and Communication: Students will need to facilitate communication between Mrs. Wright's daughter and the healthcare team and address family conflicts.
- Ethical Considerations: Students will need to address ethical dilemmas related to end-of-life care, decision-making capacity, and quality of life.

Simulation Activities:

- Behavioral Management Simulation: Students can practice de-escalation techniques and communication strategies for managing agitated and aggressive behaviors.
- Family Meeting Role-Play: Students can role-play a family meeting to discuss Mrs. Wright's care plan, address her daughter's concerns, and explore end-of-life care options.
- Interprofessional Case Conference: Students from different disciplines can collaborate to develop a comprehensive care plan for Mrs. Wright.
- Ethical Dilemma Discussion: Students can discuss ethical dilemmas related to Mrs. Wright's care, such as balancing her comfort with her daughter's wishes.

Assessment:

- Students can be assessed on their ability to:
- Assess and manage behavioral disturbances in dementia
- Understand palliative care principles and end-of-life care options
- Address nutritional needs and pressure ulcer management
- Facilitate family communication and address conflicts
- Address ethical considerations related to end-of-life care

This case study presents a challenging scenario that requires students to address the complex needs of an older adult with advanced dementia. It provides a valuable opportunity to develop skills in behavioral management, palliative care, and interprofessional collaboration, while also addressing ethical dilemmas related to end-of-life care.

# Mrs. Dorothy Wu

Patient Profile:

- Name: Dorothy Wu
- Age: 84
- Gender: Female
- Ethnicity: Chinese American
- Marital Status: Widowed
- Living Situation: Lives with her son and daughter-in-law in a single-story home
- Insurance: Medicare

Medical History:

- Chronic Obstructive Pulmonary Disease (COPD) (diagnosed 7 years ago)
- Atrial Fibrillation (diagnosed 5 years ago)
- Osteoporosis (diagnosed 12 years ago)
- Anxiety Disorder (diagnosed 4 years ago)
- Urinary Incontinence (recent onset)
- Current Medications:
- Tiotropium 18 mcg daily
- Warfarin 5 mg daily
- Alendronate 70 mg weekly
- Alprazolam 0.25 mg PRN for anxiety
- Calcium 500 mg with Vitamin D BID

Chief Complaint: Mrs. Wu presents with worsening shortness of breath, frequent episodes of urinary incontinence, and increased anxiety about her health.

Social History:

- Former accountant
- Has three children, with one living nearby and providing daily assistance
- Enjoys gardening and Tai Chi but has limited participation due to health issues
- Active in her local Chinese community center, attending cultural events and activities

Physical Examination Findings:

- BP: 135/80 mmHg
- HR: 90 bpm, irregular
- RR: 20 breaths/min
- Temp: 98.5°F
- Weight: 140 lbs, Height: 5'1"
- General: Appears anxious, using supplemental oxygen
- HEENT: No acute distress
- Cardiovascular: Irregular rhythm, no murmurs
- Respiratory: Bilateral wheezing, diminished breath sounds at bases
- Musculoskeletal: Kyphosis, limited range of motion in spine
- Neurological: Alert and oriented, mild tremor in hands

Simulation Objectives:

- Medical Students:
- Develop a comprehensive plan for managing COPD and atrial fibrillation.
- Address the impact of polypharmacy and potential drug interactions.
- Assess and manage symptoms of osteoporosis and anxiety.
- Nursing Students:
- Perform a detailed physical assessment, focusing on respiratory and cardiovascular systems.
- Develop a care plan addressing shortness of breath, incontinence, and anxiety management.
- Educate the patient on lifestyle modifications, medication adherence, and use of supplemental oxygen.
- Social Work Students:
- Assess the impact of Mrs. Wu's health on her daily life and family dynamics.
- Identify community resources and support systems for elderly patients with chronic illnesses.
- Collaborate with the healthcare team to address cultural and language barriers in care provision.

Discussion Points:

- Balancing the management of multiple chronic conditions in elderly patients.
- Importance of addressing both physical and mental health in geriatric care.
- Strategies for improving quality of life and reducing hospital readmissions.
- Role of interdisciplinary teamwork in providing comprehensive care.
- This case study encourages students to collaborate across disciplines to deliver holistic, patient-centered care for elderly patients, focusing on their unique needs and challenges.

# Mr. Takeshi Yamamoto

Patient Information

- Name: Takeshi Yamamoto
- Age: 88
- Gender: Male
- Marital Status: Widowed (3 years ago)
- Living Situation: Lives with adult son and daughter-in-law in a multi-generational home

Chief Complaint

"I fell in the bathroom last night and hurt my back. I'm also having trouble seeing clearly."

History of Present Illness

Mr. Yamamoto fell in the bathroom last night while getting up to urinate. He reports severe lower back pain and difficulty moving. His son notes that Mr. Yamamoto has been increasingly unsteady on his feet over the past few months. Mr. Yamamoto also mentions that his vision has been getting worse, making it hard for him to read and recognize faces.

Past Medical History

- Glaucoma (diagnosed 10 years ago)
- Osteoarthritis (knees and hips)
- Benign Prostatic Hyperplasia (BPH)
- Hypertension
- History of gastric ulcer (5 years ago)

Medications

- Timolol 0.5% eye drops twice daily
- Latanoprost 0.005% eye drops at bedtime
- Tamsulosin 0.4 mg daily
- Amlodipine 5 mg daily
- Acetaminophen 500 mg as needed for pain
- Omeprazole 20 mg daily

Allergies

- No known drug allergies

Social History

- Retired sushi chef
- Widowed 3 years ago; wife died of stroke
- Lives with son, daughter-in-law, and two grandchildren
- Enjoys gardening and teaching grandchildren to cook
- No history of smoking or alcohol use
- Immigrated from Japan 50 years ago, speaks English but prefers Japanese

Review of Systems

- General: Fatigue, unintentional weight loss of 3 kg in 2 months
- Musculoskeletal: Chronic knee and hip pain, acute lower back pain
- Neurological: Occasional dizziness, especially when standing up quickly
- Genitourinary: Urinary frequency, nocturia (3-4 times per night)
- Ophthalmological: Gradual vision loss, difficulty with night vision
- Gastrointestinal: Poor appetite, occasional constipation

Physical Examination

- Vital Signs:
- BP: 110/65 mmHg (lying down), 90/50 mmHg (standing)
- HR: 78 bpm (lying down), 96 bpm (standing)
- RR: 16/min
- Temp: 36.5°C (97.7°F)
- O2 Sat: 96% on room air
- General: Alert, oriented, appears frail
- Ophthalmological:
- Visual acuity: 20/100 OD, 20/80 OS
- Intraocular pressure: 28 mmHg OD, 26 mmHg OS
- Musculoskeletal:
- Tenderness over lower lumbar spine
- Limited range of motion in hips and knees
- Kyphotic posture
- Neurological:
- Decreased proprioception in lower extremities
- Positive Romberg test
- Skin: Multiple senile purpura on arms

Laboratory and Imaging Results

- Complete Blood Count (CBC):
- Hemoglobin: 11.0 g/dL (low)
- MCV: 78 fL (low)
- Basic Metabolic Panel (BMP):
- Sodium: 138 mEq/L
- Potassium: 3.3 mEq/L (low)
- Creatinine: 1.2 mg/dL
- Vitamin D level: 15 ng/mL (deficient)
- Vitamin B12 level: 220 pg/mL (low normal)
- X-ray of lumbar spine: Compression fracture of L2 vertebra, severe degenerative changes

Assessment

- Acute compression fracture of L2 vertebra
- Orthostatic hypotension
- Worsening glaucoma
- Falls risk
- Benign Prostatic Hyperplasia
- Osteoarthritis
- Vitamin D deficiency
- Possible B12 deficiency
- Microcytic anemia
- Frailty syndrome
- Hypokalemia

Plan

- Pain management for acute compression fracture
- Orthopedic consultation for management of compression fracture
- Ophthalmology consultation for management of worsening glaucoma
- Adjust antihypertensive medication due to orthostatic hypotension
- Start Vitamin D supplementation
- Further workup for anemia, including iron studies
- Consider Vitamin B12 supplementation
- Physical therapy and occupational therapy evaluations
- Nutrition consultation for weight loss and nutritional deficiencies
- Home safety evaluation
- Medication review and reconciliation

Simulation Learning Objectives

- Medical Students:
- Evaluate and manage acute back pain in the elderly
- Recognize and address multiple risk factors for falls
- Manage multiple chronic conditions in the context of an acute injury
- Develop a comprehensive geriatric assessment and care plan
- Nursing Students:
- Conduct a thorough falls risk assessment
- Implement pain management strategies for elderly patients
- Develop and implement a plan to prevent pressure ulcers in a patient with limited mobility
- Educate patient and family on proper body mechanics and safe transfers
- Social Work Students:
- Assess family dynamics in a multi-generational household
- Address cultural considerations in care planning
- Evaluate need for home modifications and assistive devices
- Explore community resources for elderly immigrants
- Interdisciplinary Objectives:
- Practice effective communication with patients who have sensory impairments
- Collaborate to develop a culturally sensitive, person-centered care plan
- Recognize and address geriatric syndromes (falls, frailty, sensory impairment)
- Develop strategies for supporting family caregivers in a multi-generational home

Simulation Scenario Progression

- Initial Assessment: Each discipline conducts their respective assessments in the emergency department.
- Team Huddle: Interdisciplinary team meets to discuss findings and develop an initial care plan.
- Family Meeting: Simulate a meeting with Mr. Yamamoto and his son to discuss diagnosis, treatment options, and cultural considerations in care.
- Acute Management: Simulate pain management and initial treatment for compression fracture.
- Discharge Planning: Team develops a comprehensive plan for home care, including family education, home modifications, and follow-up care.
- Cultural Competence: Address language barriers and cultural preferences in care planning.
- Follow-up: Simulate a home visit 2 weeks post-discharge to assess recovery, medication adherence, and effectiveness of home modifications.

This case study provides multiple learning opportunities for students across healthcare disciplines, focusing on the complex care needs of an older adult with multiple chronic conditions, acute injury, sensory impairments, and cultural considerations.
